# Why Is Wnt/β-Catenin Not Yet Targeted in Routine Cancer Care?

**DOI:** 10.3390/ph17070949

**Published:** 2024-07-16

**Authors:** Auriane de Pellegars-Malhortie, Laurence Picque Lasorsa, Thibault Mazard, Fabien Granier, Corinne Prévostel

**Affiliations:** 1 IRCM (Montpellier Cancer Research Institute), University of Montpellier, Inserm, ICM (Montpellier Regional Cancer Institute), 34298 Montpellier, CEDEX 5, France; auriane.de-pellegars@inserm.fr (A.d.P.-M.); laurence.picque@icm.unicancer.fr (L.P.L.); thibault.mazard@icm.unicancer.fr (T.M.); 2 Medical Oncology Department, ICM, University of Montpellier, CEDEX 5, 34298 Montpellier, France; 3 FGHI, 165 Rue Denis Papin, 34570 Montarnaud, France; fabien.granier@fghi.eu

**Keywords:** Wnt/β-catenin, cancer therapy, small molecule inhibitors, drug design, drug profiling, drug combination, ADC, nanovectorization, precision medicine, clinical trials

## Abstract

Despite significant progress in cancer prevention, screening, and treatment, the still limited number of therapeutic options is an obstacle towards increasing the cancer cure rate. In recent years, many efforts were put forth to develop therapeutics that selectively target different components of the oncogenic Wnt/β-catenin signaling pathway. These include small molecule inhibitors, antibodies, and more recently, gene-based approaches. Although some of them showed promising outcomes in clinical trials, the Wnt/β-catenin pathway is still not targeted in routine clinical practice for cancer management. As for most anticancer treatments, a critical limitation to the use of Wnt/β-catenin inhibitors is their therapeutic index, i.e., the difficulty of combining effective anticancer activity with acceptable toxicity. Protecting healthy tissues from the effects of Wnt/β-catenin inhibitors is a major issue due to the vital role of the Wnt/β-catenin signaling pathway in adult tissue homeostasis and regeneration. In this review, we provide an up-to-date summary of clinical trials on Wnt/β-catenin pathway inhibitors, examine their anti-tumor activity and associated adverse events, and explore strategies under development to improve the benefit/risk profile of this therapeutic approach.

## 1. Introduction

In 1984, Nusse and Varmus identified *Wnt-1*, the first gene encoding a Wnt/β-catenin pathway component, as the “first common integration site” of a mouse mammary tumor virus (MMTV) sequence responsible for the milk-transmitted susceptibility to develop mammary gland tumors in some strains of mice [1,2,3,4] (Table S1). This discovery generated great interest in the scientific community by highlighting the importance of Wnt signaling in animal species at all stages of life, from embryogenesis to regeneration and homeostasis of adult tissues [5,6,7,8,9,10,11,12,13,14] (Table S1). Concomitantly, genetic alterations associated with developmental defects in animal models, such as *Drosophila melanogaster*, *Caenorhabditis elegans*, and the mouse, or with human diseases, such as inherited and sporadic cancers, played a significant role in identifying critical components and regulatory mechanisms of this complex signaling network [15,16,17,18,19,20,21,22,23,24,25,26,27]. These studies established that Wnt is an autocrine-secreted glycoprotein that binds to atypical G-protein-coupled seven-pass transmembrane receptors (frizzled or FZD), leading to activation of three distinct and interconnected signaling pathways: the canonical Wnt pathway, also known as the Wnt/β-catenin signaling pathway, and two non-canonical pathways, the Wnt/calcium (Wnt/Ca^2+^) pathway and the planar cell polarity pathway [28,29,30,31]. To date, 19 Wnt ligands and 10 FZD receptors were identified in humans, suggesting multiple combinations of Wnt/FZD pairs to activate one of these three signaling pathways. A systematic mapping of Wnt-FZD interactions revealed distinct functional selectivity depending on the Wnt/FZD pair; however, how a specific Wnt signaling cascade is achieved still remains elusive because some Wnt/FZD combinations display functions in both canonical and non-canonical Wnt pathways [32,33,34]. In general, Wnt1, 2, 3a, 8a, 8b, 10a, 10b and FZD1, 5, 7, 9 are classified as canonical components, whereas Wnt4, 5a, 5b, 6, 7a, 7b, 11 and FZD2, 3, 4, 6 are considered non-canonical components. Wnt2b, 9a, 9b, 16 and FZD8, 10 remain unclassified. Moreover, single transmembrane receptors, such as LDL receptor-related proteins 5/6 (LRP5/6), the receptor tyrosine kinase-like orphan receptors 1/2 (ROR1/2), and the tyrosine kinase-related receptor RYK, contribute to Wnt signaling specification by acting as FZD co-receptors [35,36,37,38,39,40,41,42]. For example, ROR1 and ROR2 were associated with the activation of β-catenin-independent Wnt signaling, notably via the non-canonical ligand Wnt-5a [43]. In addition, the LRP5/6, ROR1/2 and RYK receptors can bind to Wnt ligands and transduce them on their own [44,45,46,47,48,49,50,51,52,53], thus adding another layer of complexity. 

In this review, we focused on strategies for selectively targeting canonical Wnt signals, the transduction of which relies on the intracellular stabilization and co-transcriptional activity of β-catenin. In resting cells, i.e., in the absence of extracellular Wnt signals, the cytoplasmic concentration of β-catenin is maintained constant through the balance between synthesis and proteasomal degradation [54,55,56]. β-catenin degradation is initiated by a multi-protein complex composed of the scaffold proteins axins and Adenomatous Polyposis Coli (APC) associated with the serine/threonine kinases Casein Kinase 1 α (CK1α) and Glycogen Synthase Kinase 3 β (GSK3β) [11,57,58,59,60,61,62,63,64,65,66,67]. Within this complex, GSK3β and CK1α mediate β-catenin phosphorylation, a key recognition signal for the S-phase kinase-associated protein 1 (SKP1)-Cullin1-F-box protein (SCFβ-TrCP)-dependent ubiquitination and subsequent proteasomal degradation of β-catenin [68,69]. Once initiated by the binding of an extracellular Wnt ligand to a transmembrane FZD receptor, the signal transduction is relayed inside the cell through Disheveled (DVL), which recruits the β-catenin degradation complex at the plasma membrane [70,71,72,73,74,75,76]. This prevents SCFβ-TrCP-induced β-catenin ubiquitination and degradation, leading to β-catenin accumulation in the cell, including in the nucleus [68]. There, β-catenin binds to the transcription factors lymphoid enhancer factor-1 and T-cell factor (TCF) and recruits co-transcriptional activators [B-cell lymphoma 9 (BCL9), B-cell lymphoma 9-like, Pygorus], chromatin modifiers, and remodeling factors [CREB binding Protein (CBP), Brg-1], thereby enhancing the expression of many target genes [77,78,79,80,81], including c-*MYC*. Importantly, c-*MYC* is the master gene responsible for the oncogenic activity of the Wnt/β-catenin pathway in the intestine, where APC mutations are sufficient for cancer initiation [82,83,84]. In addition to APC mutations, a significant number of genetic alterations that induce β-catenin stabilization were identified in several components of the Wnt/β-catenin signaling cascade and are considered critical events in the development of different cancer types [85,86] (Table S1). Therefore, this pathway is a major and challenging cancer research topic. Developed strategies include the use of small molecule inhibitors, antibodies, and more recently, gene-based approaches, some of which are showing promising outcomes in clinical trials. Yet, the Wnt/β-catenin pathway is not targeted for cancer management in routine clinical practice. This narrative review provides a comprehensive analysis of current clinical research and perspectives on the topic of inhibiting Wnt/β-catenin pathway activity for future implementation in routine clinical practice. Included are inhibitors of the Wnt/β-catenin pathway tested in clinical trials alone or in combination with other anticancer therapies. Excluded are anticancer agents with unproven Wnt/β-catenin pathway inhibitory activity, Wnt/β-catenin pathway inhibitors still in preclinical development, and Wnt/β-catenin pathway inhibitors whose mechanism of action is unknown.

## 2. Wnt/β-Catenin Inhibitors in Clinical Trials

Several comprehensive reviews described strategies to selectively inhibit the Wnt/β-catenin pathway [87,88,89]. These involve the use of two distinct groups of compounds that target the canonical and non-canonical Wnt/β-catenin pathways. Many of these compounds were tested in monotherapy and/or in combination with conventional chemotherapy agents in patients with cancer to determine their therapeutic index in clinical trials. This section provides an up-to-date overview of clinical trials designed to selectively target the Wnt/β-catenin pathway, and highlights the clinical benefits and adverse side effects of the Wnt/β-catenin inhibitors used as anticancer agents.

### 2.1. Clinical Trials Using Canonical Wnt-Dependent Inhibitors (WDi)

**Scheme 1:**
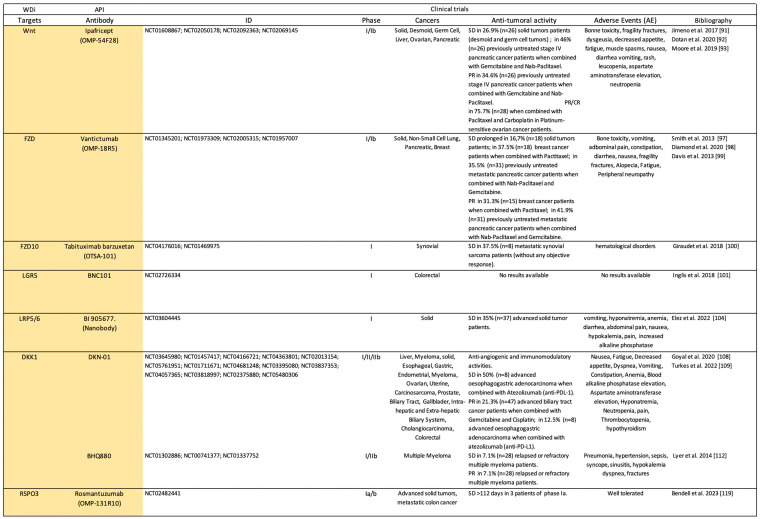
Clinical trials (https://clinicaltrials.gov/) using antibodies as Wnt-dependent inhibitors (WDi). API: active pharmaceutical ingredient; SD: stable disease; PR: partial response; CR: complete response [91,92,93,97,98,99,100,101,104,108,109,112,119].

**Scheme 2:**
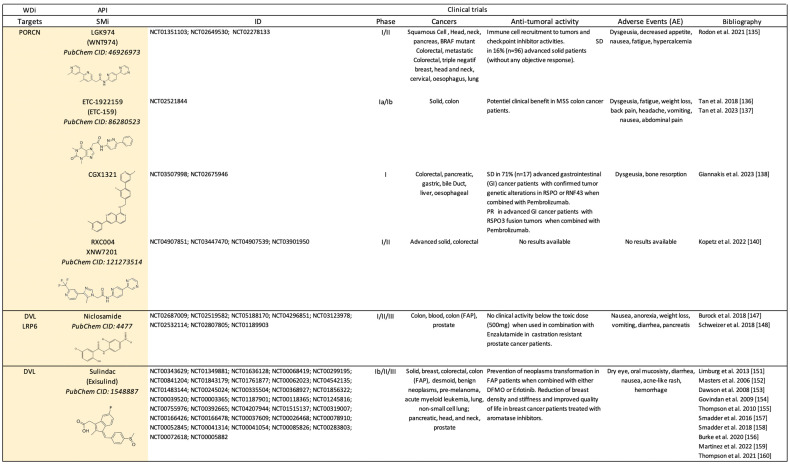
Clinical trials (https://clinicaltrials.gov/) using small molecules inhibitors (SMi) as Wnt-dependent inhibitors (WDi). API: active pharmaceutical ingredient; SD: stable disease; PR: partial response; CR: complete response [135,136,137,138,140,147,148,151,152,153,154,155,156,157,158,159,160].

Canonical WDi are antibodies (Scheme 1) or small molecules (Scheme 2) that selectively target ligands, receptors, or modulators of the canonical Wnt/β-catenin signaling pathway.

#### 2.1.1. Antibody-Based Therapies

Antibody-based strategies selectively target components exposed at the cell surface, thus representing attractive approaches for targeting Wnt/β-catenin signaling in cancer cells [90]. The developed strategies are based on recombinant fusion proteins that can trap Wnt ligands and prevent Wnt-FZD interaction, or on antibodies that can antagonize Wnt binding to FZD receptors and block Wnt signal transduction.

**Anti-Wnt molecules:** the first class of recombinant fusion proteins to trap Wnt ligands is represented by ipafricept (OMP-54F28), developed by OncoMed Pharmaceuticals. Ipafricept includes the Fc fragment of human IgG1 fused to the extracellular portion of the human FZD-8 receptor. Several phase I studies on ipafricept reported encouraging outcomes for cancer treatment. In a first-in-human phase I study (NCT01608867), ipafricept administration led to stable disease in patients with advanced solid tumors, such as desmoid and germ cell tumors, at acceptable tolerated doses [91]. In a phase I study (NCT02050178) in which ipafricept was combined with the anti-metabolic agent gemcitabine and the anti-mitotic agent nab-paclitaxel, a significant number of patients displayed partial response and stable disease and the clinical benefit rate was 81% (i.e., the best response) in patients with previously untreated stage IV pancreatic cancer [92]. A dose escalation study of ipafricept (OMP-54F28) in combination with paclitaxel and the cytotoxic agent carboplatin (NCT02092363) also showed an overall response rate of 75.7% in patients with recurrent platinum-sensitive ovarian cancer. However, bone toxicity at efficacy doses was considered an obstacle for further development of its use as ovarian cancer treatment [93]. The observed bone toxicity is not surprising given the critical role of Wnt/β-catenin signaling in bone homeostasis [94].

**Anti-Wnt receptor molecules:** Strategies to target canonical Wnt receptors mainly involved monoclonal antibodies against FZDs [95]. For instance, vantictumab (OMP-18R5) is a human IgG2 antibody against the FZD extracellular domain developed by OncoMed Pharmaceuticals, in partnership with Bayer [96]. 

The first-in-human, phase I study on OMP-18R5 in 18 patients with advanced solid tumors (NCT01345201) showed stable disease in three patients with manageable and reversible bone toxicity, thus allowing dose escalation to continue [97]. Recently published results of a phase Ib clinical trial on vantictumab combined with paclitaxel (NCT01973309) indicate promising efficacy at well tolerated doses in patients with locally advanced or metastatic HER-2 negative breast cancer. However, the incidence of bone fractures was considered as a limitation to future clinical developments in metastatic breast cancer [98]. Another phase Ib study (NCT02005315) recently reported that the combination of vantictumab with nab-paclitaxel and gemcitabine in patients with previously untreated metastatic pancreatic cancer had to be discontinued due to concerns about bone-related safety [99]. 

Tabituximab barzuxetan (OTSA-101) is a radiolabeled humanized monoclonal antibody against FZD-10 developed by OncoTherapy Science. Currently, ^111^In-radiolabeled OTSA-101 is tested in patients with relapsed or refractory synovial sarcoma (NCT01469975; NCT04176016) because a previous study with ^90^Y-radiolabeled OTSA-101 reported hematological toxicity [100]. 

BNC101, a human monoclonal antibody against the leucin-rich repeat-containing G-protein coupled receptor 5 (LGR5), was developed by Bionomic Limited and evaluated in a phase I, dose escalation study in patients with metastatic colorectal cancer (NCT02726534) [101]. LGR5 is both a Wnt/β-catenin target gene and a potentiator of Wnt/β-catenin activity through binding to its roof plate-specific spondin (R-spondin or RSPO) ligands and co-interaction with FZD [102]. LGR5 is a promising target for cancer therapy because it is overexpressed in various tumor types and stimulates cancer stem cell proliferation and self-renewal, cancer cell mobility, tumor formation, and epithelial-mesenchymal transition [103]. The clinical trial on BCN101 was completed, but the results are yet to be published. 

An emerging approach to target Wnt receptors or co-receptors involves the use of nanobodies, which are single monomeric variable domains of antibodies produced by genetic engineering. To date, BI905677 is the only nanobody targeting Wnt/β-catenin signaling tested in a clinical trial (NCT03604445). This humanized bi-paratopic nanobody developed by Boehringer is composed of two domains that block the LRP5/6 co-receptors. In this phase I open-label study, dose-escalation in patients with advanced solid tumors showed that BI905677 is well tolerated and associated with stable disease in 35% of patients [104]. 

**Anti-extracellular modulator antibodies:** The most prominent examples of antibodies against extra-cellular modulators of Wnt/β-catenin signaling target Dickkopf-1 (DKK1), a secreted Wnt/β-catenin inhibitor that prevents LRP5/6 heterodimerization with FZDs [45,105,106]. Among them, the humanized monoclonal antibody DKN-01, developed by Leap Therapeutics (Nasdaq: LPTX), showed promising outcomes in several phase I clinical trials (NCT01457417; NCT02013154; NCT02375880; and NCT01711671) and was evaluated in phase II studies for many cancer types (NCT03395080; NCT03645980; NCT05761951; NCT04057365; NCT03837353; NCT04166721; NCT03818997; NCT05480306; and NCT04363801) [107]. Recently published results for the clinical trial NCT02375880 indicate that 300 mg of DKN-01, in combination with gemcitabine and cisplatin, is well tolerated in patients with advanced biliary tract cancer. However, this combination did not seem to have additional activity compared with the gemcitabine and cisplatin combination alone [108]. As DKN-01 has potential anti-angiogenic and immunomodulatory activities, it was considered that DKN-01 dose/intensity should be increased. Therefore, a phase II study using 600 mg of DKN-01 in combination with the immune checkpoint inhibitor nivolumab [an anti-Programmed cell Death 1 (PD-1) antibody] was started for patients with advanced biliary tract cancer (NCT04057365). Recent results from a phase IIa clinical trial on DKN-01 in combination with the immune checkpoint inhibitor atezolizumab [an antibody against Programmed cell Death ligand-1 (PD-L1)] in patients with advanced esophagogastric adenocarcinoma (NCT04166721) reported a manageable safety profile without new safety signals due to DKN-01 [109]. Moreover, BHQ880, a phage-derived anti-DKK1 antibody developed by Novartis Pharmaceuticals, was evaluated in multiple myeloma because it can increase osteoblast differentiation and inhibit malignant plasma cell growth and osteolytic lesion development [110,111]. In a phase II clinical trial evaluating disease response in patients with smoldering multiple myeloma (NCT01302886), intravenous administration of BHQ880 was well tolerated and associated with stable disease in most patients. BHQ880 was also assessed in phase I/II clinical trials in combination with the proteasome inhibitor bortezomib and the anti-inflammatory and immunosuppressive agent dexamethasone in patients with untreated multiple myeloma and renal insufficiency (NCT01337752), or with zoledronic acid and standard anti-myeloma chemotherapy in patients with relapsed or refractory myeloma (NCT00741377). The published results of the second trial show stable disease in two patients, and a shift from stable disease to partial response in 2/28 patients, although the use of combination treatments did not allow for assessing BHQ880-specific effects [112]. 

Recently, two additional classes of secreted Wnt/β-catenin inhibitors attracted much interest due to exciting findings in animal cancer models [113,114]. The first targets the RPSO ligands for the LGR 4/5/6 receptors [115]. RPSO behave as potent Wnt signal enhancers and stem cell growth factors by neutralizing zinc and ring finger 3 and ring finger 43 (RNF43), two transmembrane E3 ubiquitin ligases that ubiquitinate FZDs, thereby promoting their endocytosis and degradation [116,117]. RSPO2 and RSPO3 were also identified as oncogenesis drivers in colon cancer subsets and other solid tumor types [24,118]. Rosmantuzumab (OMP-131R10), an anti-RPSO-3 monoclonal antibody developed by OncoMed Pharmaceuticals is tested in an ongoing phase I dose escalation study in patients with advanced solid tumors and metastatic colon cancer (NCT02482441). The initial results indicate that OMP-131R10 is well tolerated and three patients had prolonged stable disease for 112 days as the best objective response [119]. These encouraging observations could at least partly result from the anti-fibrosis activity of OMP-131R10 [120], fibrosis being an important player in malignant transformation, cancer aggressiveness, and response to treatment [121,122,123]. The second inhibitor class targets NOTUM, an acetylase that palmitoylates Wnt ligands, thereby preventing their binding to FZD receptors [124,125]. NOTUM inhibition abrogates the ability of APC-mutated cells to expand and form intestinal adenomas, suggesting a potential application for people at a high risk of developing colorectal cancer [114]. This inhibitor was not tested yet in clinical trials.

#### 2.1.2. Small Molecule-Based Therapies 

**Porcupine inhibitors (PORCNi):** Small molecules acting as canonical WDi in clinical trials are mostly represented by PORCNi that are designed to inhibit Wnt autocrine function by preventing both secretion and binding of Wnt ligands to FZD receptors [126,127]. PORCN is an endoplasmic reticulum-resident membrane-bound *O*-acyltransferase that mediates Wnt palmitoylation on a highly conserved hairpin, a critical post-translational modification for Wnt secretion and autocrine function [128]. Palmitoylation increases Wnt ligand hydrophobicity, trapping them close to neighboring cells and increasing their affinity for FZDs to initiate signal transduction [34]. PORCNi showed promising effects in different tumor types, including colorectal, pancreatic, hepatocellular, and head and neck cancer [25,129,130,131,132,133]. However, long-term exposure of colon cancer cells is associated with the emergence of a resistant population that carries frameshift deletions in the Wnt pathway inhibitor axin1, leading to protein loss [134]. None of the available PORCNi were marketed and only four molecules (LGK974, ETC-159, CGX1321, and RXC004) reached the phase I stage [126]. Three clinical trials on LGK974 were launched by Novartis (NCT01351103), Array bioPharma (NCT02278133), and the University of Michigan Rogel Cancer Center (NCT02649530). This last trial was withdrawn for undisclosed reasons. Recent published results from a single-agent phase I study on the PORCNi WNT974 (NCT01351103) in patients with advanced solid cancer reported an effect on immune cell recruitment to the tumor and checkpoint inhibitor activity with limited anti-tumoral activity [135]. Similarly, a phase I expansion study (NCT02521844) in which the PORCNi ETC-159 was administered with bone protective treatment showed increased immune infiltration in advanced tumors [136]. However, a phase Ib dose escalation study in which ETC-159 was combined with the immune checkpoint inhibitor pembrolizumab (anti-PD-1 antibody) in advanced or metastatic solid tumors was discontinued due to disease progression in 90% of the included patients and significant side effects, despite potential clinical benefit in patients with microsatellite stable (MSS) colon cancer [137]. CGX1321, the PORCNi developed by Curegenix, strongly inhibits the Wnt pathway with manageable side effects when administered alone or in combination with pembrolizumab in patients with advanced gastrointestinal tumors (NCT02675946; NCT03507998). In combination with pembrolizumab, CGX1321 showed promising efficacy results in patients with tumors carrying RSPO fusions. This supports its further development as monotherapy and in combination with anti-PD-1/L1 antibodies for this cancer type that is refractory to standard therapies and to immune checkpoint inhibitors [138]. CGX1321 is currently tested as an oral treatment in patients with relapsed or refractory solid tumors, including colorectal, gastric, pancreatic, bile duct, liver, and esophageal carcinoma [139]. RXC004, the PORCNi developed by RedxPharma, also showed promising results in a phase I study in patients with advanced solid tumors (NCT03447470) and was tested in a multi-arm phase II open-label study (NCT04907539), as monotherapy or in combination with the anti-PD-1 nivolumab, in patients with RNF43- or RSPO-mutated, metastatic, and MSS colorectal cancer following standard treatments. Results are not available yet [140]. Recently, it was reported that VHN-88, a novel PORCNi, limits progression of xenografted Wnt-driven human teratocarcinoma with high autocrine Wnt signaling and pancreatic carcinoma with Wnt-sensitizing RNF43 mutations and inhibits cancer cell stemness [141]

**DVL inhibitors:** Drugs initially used for different purposes were found to behave as FZD inhibitors. For instance, niclosamide, a drug used as an anti-helminthic since the mid-1960s and approved by the Food and Drug Administration (FDA) and the European Medicines Agency (EMA) for treating tapeworm infections, interferes with Wnt/β-catenin signaling by promoting FZD1 internalization, leading to DVL-2 down-regulation, and also by inducing degradation of the LRP6 transmembrane receptor [142,143,144,145,146]. Niclosamide was assessed in a phase II clinical trial (NCT02519582). An ongoing study investigates its safety and efficacy by oral administration in patients with metachronous or synchronous metastases of colorectal cancer that progressed after therapy [147]. However, in a phase I study in patients with castration-resistant prostate cancer treated with the androgen receptor inhibitor enzalutamide, oral administration of niclosamide did not show any clinical activity at safe doses (below 500 mg) [148]. Furthermore, the FDA-approved non-steroidal anti-inflammatory drug sulindac prevents interaction of the PDZ domain of DVL with FZD, thereby suppressing Wnt-induced β-catenin signaling [149,150]. Unfortunately, sulindac did not show any significant clinical benefit in patients with lung or prostate cancer [151,152,153,154]. Conversely, phase IIb/III clinical trials (NCT00005882, NCT00118365) suggested that the combination of sulindac with the cytostatic agent eflornithine (difluoromethylornithine or DFMO) might prevent colorectal adenoma development in predisposed patients [155]. However, in a phase III clinical study in patients with Familial Adenomatous Polyposis (FAP) (NCT01483144), the eflornithine-sulindac combination did not significantly reduce disease progression rate compared with eflornithine alone [156]. A secondary analysis of a randomized clinical trial in patients with FAP (NCT01187901) indicated that sulindac combined with the EGFR inhibitor erlotinib significantly decreased the number of colorectal polyps after 6 months of treatment [157,158]. More recently, reduced breast density, as well as improved stiffness and quality of life were correlated with sulindac treatment in women treated with aromatase inhibitors for breast cancer (NCT00245024; NCT01761877) [159,160].

### 2.2. Clinical Trials on Canonical Wnt-Independent Inhibitors (WIi)

**Scheme 3:**
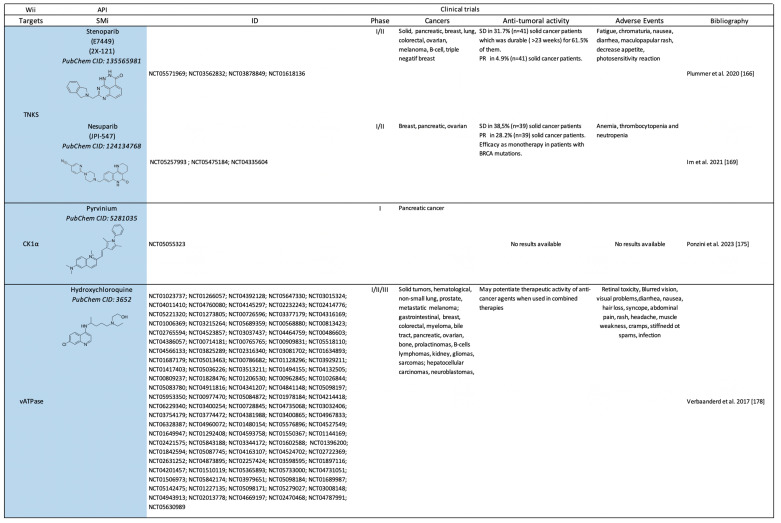

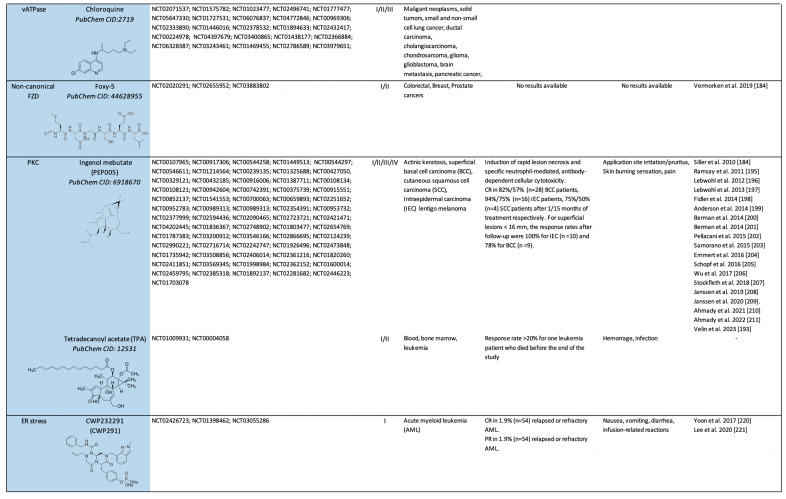
Clinical trials (https://clinicaltrials.gov/) using small molecules inhibitors (SMi) as Wnt-independent inhibitors (WIi) preventing β-catenin stabilization. API: active pharmaceutical ingredient; SD: stable disease; PR: partial response; CR: complete response [166,169,175,178,184,193,195,196,197,198,199,200,201,202,203,204,205,206,207,208,209,210,211,220,221].

**Scheme 4:**
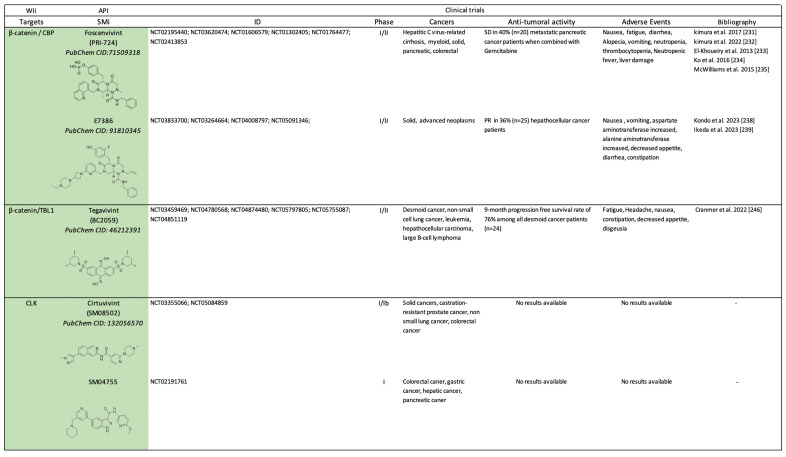
Clinical trials (https://clinicaltrials.gov/) using small molecules inhibitors (SMi) as Wnt-independent inhibitors (WIi) preventing β-catenin transcriptional activity. API: active pharmaceutical ingredient; SD: stable disease; PR: partial response; CR: complete response [231,232,233,234,235,238,239,246].

Canonical WIi include small molecules that target β-catenin and components implicated in the modulation of β-catenin stabilization (Scheme 3) or transcriptional activity (Scheme 4). 

#### 2.2.1. Small Molecule-Based Therapies to Prevent β-catenin Stabilization 

**Tankyrase inhibitors (TNKSi):** TNKSi are usually dual inhibitors of poly(ADP-ribose)polymerase I and tankyrase 1/2 (TNKS1/2). TNKS1/2 stabilize axin, the concentration-limiting component of the β-catenin degradation complex [161,162,163,164]. Interestingly, it was reported that short-form APC mutations are potential biomarkers of TNKSi sensitivity in colorectal cancer [165]. TNKSi are represented by stenoparib (also known as E7449, XAV939, or 2X-121) and nesuparib (JPI-547) [162]. In a clinical trial by the Japanese company Eisai, in patients with advanced solid tumors (NCT01618136), E7449 showed anti-tumor activity. Among the 41 patients with acceptable tolerability, 13 displayed durable stable disease and 2 partial response [166]. This study also identified the 2X-121 drug response predictor as a novel tumor-agnostic molecular biomarker to distinguish responders from non-responders to E7449. The TNKSi JPI-547 developed by Onconic Therapeutics also showed promising anti-tumor activity in Wnt-addicted pancreatic cancer cells and in BRCA-deficient breast and ovary cancer cell xenografts, as a single-agent or in combination with chemotherapy drugs and immune checkpoint inhibitors, thus encouraging the design of clinical trials to assess this drug [167,168]. Preliminary results from a phase I dose escalation and expansion study in patients with advanced solid tumors (NCT04335604) report 11 patients with confirmed partial response and 15 with stable disease among 39 patients with breast or ovarian cancer with germline or somatic BRCA/homologous recombination repair (HRR) mutations. The overall response rate of 28.2% and the disease control rate of 64.1% suggest that JPI-547 monotherapy is effective in patients with BRCA/HRR mutations [169]. Moreover, JPI-547 is currently being evaluated for the treatment of fallopian tube cancers, primary peritoneal, and non-small cell lung cancer [170].

**CK1 inhibitors:** The FDA-approved anti-helminthic drug pyrvinium was first described in 1946 as part of the US patent number 2,515,912 filed by Lare E.V. and Brooker L.G.S, and was then found to inhibit β-catenin degradation by stimulating its CK1α-induced phosphorylation [171,172,173]. Pyrvinium is effective in several cancer types, and particularly in cancer stem cells [174], and was recently assessed in a phase I clinical trial that included patients with early stage pancreatic ductal adenocarcinoma (NCT05055323) [175]. 

**V-ATPase inhibitors:** v-ATPase inhibitors prevent the inhibition of transmembrane protein 9 (TMEM9)-v-ATPase-induced vesicular acidification, thereby protecting APC from lysosomal degradation and enhancing β-catenin degradation [176]. For instance, chloroquine (CQ) and hydroxychloroquine (HCQ) display effects in cancer cells and the tumor microenvironment when used as monotherapy, and enhance the effects of chemotherapy when used as adjuvants in combination therapies [177,178]. HCQ and CQ are anti-malarial drugs chemically related to quinacrine and also the most commonly used drugs for acute and chronic inflammatory diseases, such as rheumatoid arthritis, systemic lupus erythematosus, Sjogren’s syndrome, and sarcoidosis. Several preclinical studies showed that HCQ and CQ sensitize the chemotherapy effects in many tumor types, including central nervous system, lung, breast, pancreas, leukocytes, skin, and colon and/or rectum cancers. Unfortunately, HCQ and CQ showed poor outcomes, particularly due to non-specific biodistribution, low aqueous solubility, low bioavailability at target sites, limited transport across tissue barriers, and multiple adverse events such as retinal toxicity, diarrhea, and hair loss.

**Non-canonical Wnt signaling activators:** Small molecules that act as non-canonical Wnt agonists can indirectly enhance β-catenin degradation. For instance, the Wnt5A-mimicking peptide Foxy-5, developed by WNTResearch AB, activates the non-canonical Wnt/Ca^2+^ pathway and might inhibit Wnt/β-catenin signaling at least partly through activation of the serine/threonine Protein Kinase C alpha (PKCα). Then, PKCα directly phosphorylates β-catenin and the orphan receptor RORα, increasing β-catenin degradation and inhibiting its co-transcriptional activity [179]. Given its promising anti-tumoral activity in preclinical models of colon, breast, and prostate cancer [180,181,182,183], Foxy-5 was evaluated in phase I and II clinical trials in patients with colon, breast, or prostate cancer (NCT02020291; NCT02655952) and in a phase II study in patients with colon cancer (NCT03883802) [184]. Encouraging findings on its efficacy in impairing metastasis formation in patients with cancers with low or absent *WNT5A* expression recently led to the patent application US20210008149 for Foxy-5 involvement in cancer relapse treatment and prevention [185]. 

**PKC activators:** PKCs are relevant targets for cancer therapy [186,187]. Several PKC family members behave both as tumor suppressors and Wnt/β-catenin signaling inhibitors [188,189]. For instance, PKCδ induces apoptosis and promotes β-catenin degradation through a GSK3β and β-TrCP-independent mechanism [190]. Moreover, PKCζ, similar to PKCα, behaves as a tumor suppressor in the intestine and induces β-catenin degradation, but through a phosphorylation-dependent mechanism distinct from that of PKCα [191,192]. PKC activators include the macrolide lactone bryostatin that is slightly more selective for PKCɛ, but that did not show any significant benefit in clinical cancer trials. Conversely, di-terpene esters have a good affinity for PKCα and δ and are more promising candidates in some cancers. For example, the di-terpene ester ingenol mebutate (PEP005), commercialized by Peplin, is a traditional home remedy for warts and corns, and showed anticancer and pro-inflammatory effects in several clinical trials when topically applied on skin for treating pre-malignant and malignant lesions [193,194,195,196,197,198,199,200,201,202,203,204,205,206,207,208,209,210,211]. PEP005 might have a dual mechanism of action: rapid lesion necrosis and specific neutrophil-mediated, antibody-dependent cellular cytotoxicity [212]. Several clinical trials (NCT01325688, NCT00329121, NCT00432185, NCT00108134, NCT00108121, NCT02723721, NCT03546166, NCT02990221, and NCT03569345) assessed its effect as a topical treatment of basal cell carcinoma, squamous cell carcinoma, and intraepidermal carcinoma and a phase I/II clinical study showed good results for the topical treatment of non-melanoma skin cancers [195]. Conversely, the published results of the prospective study NCT03546166 suggest that PEP005 does not bring any added value to the existing therapeutic options for low-risk superficial basal cell carcinoma and cannot be recommended for this indication [193]. Moreover, PEP005 was not considered for the management of deep-seated tumors due to the serious risk of toxicity following systemic administration. Phorbol-12-myristate-13-Acetate (PMA), another diterpene ester, promotes tumor formation when repeatedly applied on mice skin. This potentially discouraged its clinical use; however, upon systemic administration, PMA showed significant clinical benefits in patients with leukemia, and led to temporary remission in patients with myeloid leukemia refractory to conventional therapies [213,214,215]. More recently, two clinical trials were launched to assess the systemic administration of PMA in patients with hematologic malignancies (NCT00004058; NCT01009931). One patient with leukemia treated with PMA, dexamethasone, and the non-steroidal anti-inflammatory drug choline magnesium trisalicylate (Trilisate) experienced severe side effects, including gastrointestinal tract and central nervous system hemorrhages, and died before the study ended (NCT01009931).

**Endoplasmic reticulum stress activators:** WIi can also promote β-catenin degradation, but independently from the degradation complex. For instance, CWP232291 (CWP291) induces apoptosis through endoplasmic reticulum stress activation [216,217,218,219]. In a phase I study conducted by JW Pharmaceuticals in patients with relapsed or refractory acute myeloid leukemia and myelodysplastic syndrome (NCT02426723), CWP232291 monotherapy demonstrated anti-tumor activity with acceptable side effects for further enrollment in a combination therapy arm [220]. More recent results from a phase I study on CWP232291 in 54 patients with relapsed or refractory acute myeloid leukemia and myelodysplastic syndrome (NCT01398462) include one partial and one complete response. The most common adverse events were nausea in almost 50% of patients, vomiting in more than 33% of patients, and diarrhea in more than 25% of them [221].

#### 2.2.2. Small Molecule-Based Therapies to Prevent β-catenin Co-Transcriptional Activity

**Inhibitors of β-catenin-CBP interaction:** Among the WIi that prevent β-catenin co-transcriptional activity, PRI-724 (or the active agents ICG001 and C82) was developed by PRISM Pharma to specifically target the interaction between β-catenin and its transcriptional co-activator CBP [222,223,224,225]. PRI-724 promoted immune cell infiltration in gliomas, enhancing the immunotherapy effects [226]. PRI-724 represents the second generation of specific CBP-β-catenin interaction antagonists that were primarily developed for treating fibrosis-associated diseases [227,228,229,230]. In a dose escalation phase I trial in patients with hepatitis C virus-related cirrhosis (NCT02195440), PRI-724 showed dose-dependent plasma exposure and led to improvement in 3/14 patients. However, it was associated with serious adverse events, including liver damage, nausea, and vomiting [231]. Recent results from a phase I/IIa study (NCT03620474) also reported serious adverse effects, such as nausea and diarrhea, without significant decrease in hepatic fibrosis [232]. However, in a first-in-human phase I study in patients with advanced solid tumors (NCT01302405), PRI-724 showed acceptable toxicity [233]. This safety profile was confirmed in a phase Ib trial in patients with metastatic pancreatic cancer where the PRI-724-gemcitabine combination had modest clinical activity (NCT01764477), warranting next-phase clinical trials [234,235]. E7386, a non-specific CBP-β-catenin interaction antagonist developed by Eisai, also showed anti-tumor activity in pre-clinical tumor models with activated Wnt/β-catenin signaling [236,237]. E7386 is currently clinically tested alone (NCT03833700; NCT03264664) or in combination with chemotherapy drugs (NCT04008797; NCT05091346) in patients with solid tumors. In a dose-escalation study in patients with advanced solid tumors (NCT03833700), oral administration of 120mg of E7386 was well tolerated and was considered the recommended dose for the expansion study [238]. In a phase I study (NCT04008797), the combination of E7386 with the multi-kinase inhibitor lenvatinib showed promising activity in patients with hepatocellular carcinoma. Toxicity could be managed by administering antiemetics [239].

**Inhibitors of the β-catenin-transducin β-like protein 1 (TBL1) interaction:** Tegavivint (BC2059) is a WIi developed by Iterion Therapeutics that selectively disrupts the interaction of nuclear β-catenin with TBL1, a key player in enhancing β-catenin co-transcriptional activity by recruiting it to the promoter of Wnt target genes [240,241,242,243]. TBL1 also protects β-catenin from proteasomal degradation through binding to the SKP1/Cullin-1/F-box protein complex (SCF complex) [244,245]. Given its anti-tumor activity in desmoid cancer cells [241], tegavivint safety was evaluated in patients with desmoid cancer in a phase I, open-label, non-randomized study (NCT03459469). Tegavivint was well tolerated with an overall response of 25%, warranting its continued development for desmoid tumors [246]. Tegavivint is also assessed in other cancer types, including metastatic EGFR-mutated non-small cell lung cancer (NCT04780568), leukemia (NCT04874480), hepatocellular carcinoma (NCT05797805), and relapsed or refractory B-cell lymphoma (NCT05755087; NCT04851119). 

**Inhibitors of CDC-like kinases (CLKs):** Small molecule inhibitors can also behave as indirect inhibitors of β-catenin transcriptional activity by disrupting the gene expression machinery. For instance, cirtuvivint (SM08502), a pan-inhibitor of intranuclear CLK developed by Biosplice Therapeutics [247], inhibits Wnt/β-catenin activity by preventing serine and arginine-rich splicing factor (SRSF) phosphorylation, thereby disrupting spliceosome activity and blocking activation of Wnt/β-catenin target genes [248,249,250]. SM08502 showed anti-tumor activity in gastrointestinal cancer models with reduced Wnt pathway activity [247]. It is currently evaluated alone (NCT03355066) or in combination with hormonal therapies or chemotherapy agents NCT05084859) in patients with advanced solid tumors. Biosplice Therapeutics is currently developing SM04755, a CLK2 and dual-specificity tyrosine phosphorylation-regulated kinase 1A (DYRK1A) inhibitor, as an experimental treatment for tendinopathy [251]. Moreover, SM04755 safety and pharmacokinetic profiles were assessed in a phase I, open-label, dose escalation, dose-finding study (NCT02191761) in patients with advanced gastrointestinal cancer.

In addition to those evaluated in clinical trials, many Wnt/β-catenin signaling inhibitors are used for basic research. Other molecules underwent drug repurposing and are currently clinically tested in several cancer types to inhibit Wnt/β-catenin signaling. These include dietary phytochemicals, such as naringenin, resveratrol, avenanthramides, epigallocatechin, curcumin, quercetin, silibinin, genistein, mangiferin, and many others that are considered serious options for cancer chemoprevention and treatment, given their anti-stem cell, anti-metastasis, and anti-inflammatory activities [252,253,254,255,256,257,258]. Moreover, increasing technological improvements provide opportunities for developing new selective Wnt/β-catenin inhibitors for many diseases, the incidences of which are increasing (e.g., cancer, Alzheimer’s disease, and osteoporosis). Biosplice Therapeutics, Samil Pharmaceutical, Prism Pharmaceutical, Ohara Pharmaceutical, and Eisai are among the leading players in this growing market. However, none of the compounds selectively designed to inhibit the Wnt/β-catenin pathway are approved by FDA or EMA.

## 3. Future Challenges

As for the vast majority of anticancer treatments, a critical limitation of Wnt/β-catenin inhibitors is their therapeutic index (i.e., the difficulty of combining effective anti-cancer activity with acceptable toxicity) due to the crucial role of the Wnt/β-catenin pathway in maintaining the undifferentiated state of stem cells and in making cell fate decisions throughout life to preserve adult tissue homeostasis or for regeneration, for instance after injury. These vital functions explain why mutations in components or regulators of this pathway promote cancer development by increasing cancer cell growth and survival, or by acting on the tumor microenvironment [114,259,260,261], and also why protecting healthy tissues from Wnt/β-catenin inhibitors must be a major concern. Many adverse events were observed during the administration of Wnt/β-catenin inhibitors as monotherapies or in combination with other anticancer drugs (Scheme 1, Scheme 2, Scheme 3 and Scheme 4). The most common are nausea, vomiting, fatigue, diarrhea, neutropenia, headache, bone marrow toxicities, fractures, and hemorrhage. Furthermore, due to the patients’ quality of life deterioration, dose escalation was sometimes interrupted before reaching the effective anti-tumoral concentration, leading to inconclusive data about the anticancer activities of the tested Wnt/β-catenin inhibitors. This section explores current approaches to improve their efficacy while limiting harmful side effects.

### 3.1. Drug Profiling

The molecular mechanisms underlying Wnt/β-catenin signaling regulation are very complex and the homeostasis of healthy tissues is highly dependent on Wnt signaling. Therefore, the design of anticancer drugs that selectively and efficiently decrease the oncogenicity of this pathway in cancer cells is a major challenge. Gene mutations, high-resolution multi-omics data, advances in biotechnologies, and a better understanding of cancer mechanisms provided valuable information that can be used to identify relevant therapeutic targets and design new anticancer drugs, including Wnt/β-catenin inhibitors. However, experimental drug design is costly and time-consuming, without guarantee of success (~2.8 billion dollars and 10 to 17 years are needed to bring a new drug into the clinic, and only 10% of all compounds evaluated in clinical trials will reach the market) [262]. Since the 1980s, computer-aided drug design (or in silico virtual screening) significantly improved the cost-effectiveness and efficiency of screening large libraries of compounds. This led to the identification of many anticancer drug candidates, including some Wnt/β-catenin inhibitors, such as the TNKS1/2 inhibitor LZZ-02 [263] and small molecules to block β-catenin-TCF4 interactions [264,265]. Thanks to the considerable advances in computer hardware and deep neural networks, artificial intelligence is now emerging as a more powerful tool to design anticancer drugs, some of which successfully entered phase II/III clinical trials in recent years [266]. To our knowledge, so far, no artificial intelligence-designed Wnt/β-catenin inhibitor was tested in clinical trials.

### 3.2. Drug Combinations

Numerous studies demonstrated the superiority of combination therapy over monotherapies for cancer management. Combination therapies often display greater effects than the sum of the effects expected with each drug on its own. In addition, multi-target synergy can achieve therapeutic efficacy, while overcoming adverse events thanks to the administration of lower doses of each drug. For example, as niclosamide clinical use in patients with cancer is limited by its water solubility, safety, and resistance, it was combined with chemotherapeutic drugs, targeted drugs, radiotherapy, and immunotherapy to enhance its anti-tumor effects [267]. Drug combinations can also allow the rapid and cost-effective implementation of therapeutic alternatives because the different therapeutic agents may be chosen directly from the existing pharmacopoeia. Optimizing drug combination strategies was a research topic for more than 20 years, leading to the development of high-throughput combination strategies to systematically test combinations of thousands approved drug ingredients, emerging therapeutics, and research probes against different cell phenotypes that represent different diseases [268,269]. Quantitative methods were developed to determine the dose ratios that maximize the intended effect and minimize the toxic effects [270,271]. However, all experimental models still present some limitations and must be wisely chosen in function of the available data and study type (in vitro, in vivo, or clinical trial). As described in Section 2, several therapeutic combinations that include Wnt/β-catenin inhibitors were evaluated in different cancer types; for instance, the anti-Wnt antibody ipafricept (OMP54F28) or the anti-FZD antibody vantictumab (OMP18R5) with gemcitabine and nab-paclitaxel (NCT02050178; NCT02005315) [92,99]. Ipafricept was also combined with paclitaxel alone (NCT019703309) or with the cytotoxic compound carboplatin (NCT02092363) [93]. Despite promising efficacy results at well-tolerated doses, bone toxicity is a major issue with these combinations and is considered a serious limitation for future clinical developments. The combination of the anti-DKK1 antibody DKN-01 with nab-paclitaxel and gemcitabine did not show any benefit compared with nab-paclitaxel and gemcitabine alone in advanced biliary tract cancer [108]. A more promising combination is the administration of Wnt/β-catenin inhibitors with immune checkpoint inhibitors. Overexpression of immune checkpoint molecules in the tumor microenvironment has a critical role in anti-tumor immunity evasion and cancer progression; however, immune checkpoint inhibitors showed clinical benefit only in a subset of patients, suggesting immunosuppressive mechanisms within tumors [272,273]. Interestingly, the Wnt/β-catenin pathway is recognized as an important oncogenic signaling pathway related to immune evasion. Particularly, increased expression or activity of β-catenin was correlated with impaired recruitment of immune cells in tumors, a poor prognostic factor [274]. Therefore, the combination of Wnt/β-catenin inhibitors with immune checkpoint inhibitors should increase immune cell infiltration and the tumor sensitivity to immune checkpoint therapy. Several immune checkpoint inhibitors, including nivolumab (anti-PD-1 antibody), pembrolizumab (anti-PD-1 antibody), and atezolizumab (anti-PD-L1 antibody), were tested in combination with Wnt/β-catenin inhibitors in clinical trials. For example, the DKN-01 antibody was combined with nivolumab (NCT04057365) and atezolizumab (NCT04166721) for the treatment of biliary tract and esophagogastric cancer [109], and the PORCNi ETC-159 and CGX1321 were combined with pembrolizumab for metastatic solid cancer (NCT02521844) and gastrointestinal cancer management (NCT02675946; NCT03507998) [137,138]. The ETC-159-pembrolizumab combination resulted in a significant increase in immune cell infiltration in solid tumors, but with significant adverse events. The CGX1321-pembrolizumab combination showed efficacy in gastrointestinal tumors harboring RPSO fusions. An ongoing study is assessing the RXC004-nivolumab combination (NCT049075539) in patients with RNF43- or RSPO-mutated, metastatic, and MSS colorectal cancer following standard treatments [140].

### 3.3. Drug Targeting

Drug targeting is an attractive strategy to selectively deliver active concentrations of anticancer drugs at the tumor site, while protecting healthy tissues from the drug toxicity. The aim of current studies is to enhance the cytotoxic activity of agents that selectively target Wnt/β-catenin components at the cancer cell surface [for instance, using antibody-drug conjugates (ADCs)] and to preferentially deliver Wnt/β-catenin inhibitors in cancer cells (using targeting systems such as nanovectorization approaches) in order to increase their efficacy and safety. 

#### 3.3.1. ADC-Based Approaches

**Scheme 5:**
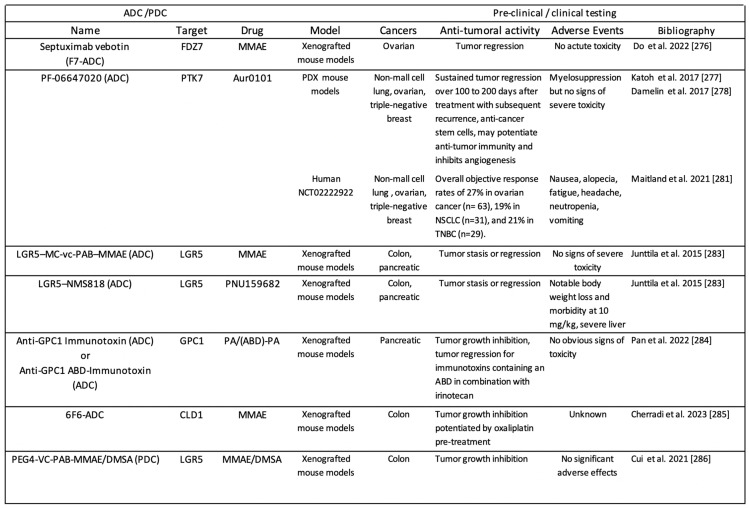
Antibody-drug conjugates (ADC) and peptide-drug conjugates (PDC) targeting the Wnt/β-catenin signaling. MMAE: Monomethyl Auristatin E; Aur0101: Auristatin Microtubule Inhibitor; PNU159682: DNA damaging topoisomerase-inhibiting anthracycline; (ABD)-PA: (Albumin Binding Domain)- Pseudomonas endotoxin A; ABD; DMSA: Streptomyces Duocarmycin [276,277,278,281,283,284,285,286].

To date, thirteen ADCs were approved by the FDA for cancer management [275]. An ADC is made of a cytotoxic molecule chemically linked to a monoclonal antibody, which can selectively target biomolecules expressed at the surface of cancer cells, be internalized through endocytosis routes, and deliver the cytotoxic drug inside the cell. Several ADCs that selectively target Wnt signaling gave promising results in preclinical studies (Scheme 5). In septuximab vedotin (F7-ADC), a human anti-FZD7 antibody is conjugated to the anti-mitotic microtubule inhibitor monomethyl auristatin E (MMAE). In preclinical models, this ADC induced ovarian tumor regression without acute toxicities [276]. PF-06647020, a PTK7-targeted ADC made of a humanized antibody against PTK7 conjugated with the auristatin microtubule inhibitor Aur0101, also induced tumor regression in a subset of patient-derived xenograft models, without significant signs of toxicity [277,278]. PTK7 is an atypical receptor tyrosine kinase family member without intrinsic tyrosine kinase activity, but with key roles during embryogenesis and carcinogenesis. PTK7 can modulate the Wnt and VEGF pathways and has a dual role in Wnt signaling: it can heterodimerize with FZD7 and bind to Wnt-2b to inhibit the canonical Wnt/β-catenin pathway [279], but it can also heterodimerize with ROR2 and bind to Wnt-5a to activate the non-canonical Wnt/planar cell polarity pathway [280]. As PTK7-dependent signaling can be oncogenic or tumor suppressive, PTK7-targeted ADCs were expected to be useful only for patients with cancer in which PTK7 is upregulated. In line with this, a first-in-human study to evaluate PF-06647020 in patients with solid tumors (NCT02222922) showed that in responders, PTK7 was moderately or strongly expressed in the tumor [281]. The most common adverse events related to PF-06647020 administration every 3 weeks were nausea, alopecia, fatigue, headache, neutropenia, and vomiting. Two LGR5-targeting ADCs (LGR5–MC-vc-PAB–MMAE and LGR5–NMS818) were developed by MedChemExpress to target LGR5-positive tumor-initiating cells and cancer stem cells. These ADCs are composed of a specific anti-LGR5 antibody conjugated to two cleavable linker drugs: the antimitotic microtubule inhibitor MMAE or the DNA damaging topoisomerase-inhibiting anthracycline PNU159682. The encouraging preclinical efficacy and safety findings supported their further evaluation in patients with gastrointestinal cancer [282,283]. Recently, it was shown that in a preclinical xenograft model, glypican 1 (GPC1) targeted immunotoxins, derived from a functional domain of Pseudomonas endotoxin A, inhibit pancreatic tumor growth via degradation of internalized GPC1, downregulation of Wnt signaling, and inhibition of protein synthesis [284]. GPC1 is a cell surface heparan sulfate proteoglycan that is overexpressed in different cancer types, including pancreatic cancer. As the 5-year survival rate of patients with pancreatic cancer receiving the standard therapies is poor (9%), GPC1-targeting ADCs might represent attractive therapeutic candidates. A recently published study reported that ADCs targeting the tight junction protein claudin-1 (CLDN1) could be relevant to circumvent acquired resistance to chemotherapy and improve outcome in patients with advanced colon cancer [285]. CLDN1 upregulation after exposure to conventional chemotherapies used in colon cancer is, at least in part, functionally related to activation of the MAPKp38/GSK3β/Wnt/β-catenin pathway. In xenograft mouse tumor models, an MMAE-conjugated anti-CLD1 monoclonal antibody (6F6-ADC) inhibited tumor growth. Moreover, sequentially combining oxaliplatin with an anti-CLDN1-ADC could be beneficial for patients with chemotherapy-resistant cancer. 

A novel approach involves the design of peptide-drug conjugates. For instance, PEG4-VC-PAB-MMAE is made of a mutated RSPO4 peptide sequence fused to the N-terminus of human IgG1-Fc and conjugated with the cytotoxin MMAE or duocarmycin (DMSA) by site-specific conjugation. The resulting peptide-drug conjugate showed potent cytotoxic effects in cancer cell lines that express any LGR in vitro and suppressed tumor growth in vivo without inducing significant adverse effects [286].

#### 3.3.2. Nanovectorization-Based Approaches

The physical–chemical properties of anticancer agents, such as stability and/or solubility, can considerably compromise the drug availability at the tumor site, requiring the administration of high drug concentrations, which can significantly increase the risk of adverse events. Nearly 400 clinical trials are currently investigating nanodelivery systems to improve drug pharmacokinetics and pharmacodynamics. However, very few of these delivery systems were approved by FDA and EMA for cancer treatment since disclosure of the first one, Doxil, in 1995. For more than ten years, strategies were developed to nanovectorize Wnt/β-catenin inhibitors [287,288,289]. Many delivery systems were designed to improve the therapeutic index of poor water-soluble phytochemicals, such as naringenin, curcumin, resveratrol, melatonin, and many others [258,290,291,292,293,294]. For example, the therapeutic index of nimbolide, a neem (*Azadirachta indica*) limonoid with poor pharmacokinetic and bioavailability profiles, is significantly enhanced when encapsulated in poly(lactic-co-glycolic acid) nanoparticles (Nim-NPs) [295]. Nim-NPs inhibit Wnt/β-catenin signaling by downregulating DNA methyltransferases, thus epigenetically restoring the expression of the secreted frizzled-related protein 1 (SFRP1) and resulting in tumor growth and metastasis formation inhibition without systemic toxicity. Similarly, the anti-tumor effects of poorly soluble repurposed drugs, such as the anti-helminthic drug niclosamide, are significantly enhanced when formulated as lipid-based nanoparticles (LNPs) [146,296]. Small molecule disruptors of β-catenin-BCL9 interaction also attracted interest for nanoformulation [297]. These include therapeutic peptides loaded onto gold nanoparticles (AuNPs) to overcome the pharmacological obstacles of peptide-derived therapeutics, such as low nuclease stability and low membrane permeability. AuNPs can successfully deliver β-catenin-BCL9 interaction-disrupting peptides into cancer cells to inhibit Wnt/β-catenin signaling and tumor growth with favorable biosafety and biocompatibility [298,299]. Nanoformulations of short non-coding RNA sequences, such as short interfering RNAs (siRNAs) and microRNAs (miRNAs), also emerged as relevant options for selectively targeting oncogenic pathways in cancer [300,301,302,303,304]. Some use LNPs to deliver mRNAs that can downregulate the expression of oncogenes, such as the MYC transcription factor, or upregulate the expression of tumor suppressor genes, such as the gene encoding alpha CCAAT enhancer-binding protein (CEBPA, also known as C/EBPα). For example, DCR-MYC is a first-in-class Dicer-substrate small interfering double-stranded RNA (DsiRNA) to target MYC. It was developed by Dicerna Pharmaceuticals as a stable LNP suspension for cancer treatment. DCR-MYC is well tolerated and showed promising initial clinical and metabolic responses across various dose levels in several cancer types (NCT02110563) [305]. However, the early efficacy results from clinical trials in patients with hepatocellular carcinoma (NCT02314052) do not meet Dicerna expectations to allow further development. OTX-2002, another LNP mRNA targeting MYC, was developed by Omega Therapeutics. Currently, an open-label phase I/II study evaluates OTX-2002 safety, tolerability, pharmacokinetics, pharmacodynamics, and preliminary anti-tumor activity as a single agent and in combination with the standard of care in patients with hepatocellular carcinoma or other solid cancers related to the MYC oncogene (NCT05497453) [306]. Encouraging safety findings were described in a small cohort of patients with hepatocellular carcinoma [307]. Mina Therapeutics developed MTL-CEBPA, a nanoformulated small double-stranded 2′-O-methylated RNA that can specifically activate the expression of the tumor suppressor CEBPA, preventing activation of Wnt/β-catenin signaling [308,309]. In a first-in-human study in patients with advanced hepatocellular cancer (NCT02716012), MTL-CEBPA displayed a good safety profile and potential anti-tumor activity when followed by treatment with tyrosine kinases inhibitors [310]. This prompted the evaluation of MTL-CEBPA combined with the multi-kinase inhibitor sorafenib in a randomized phase II trial in patients with hepatocellular carcinoma (NCT04710641). MTL-CEBPA is also evaluated in ongoing phase I clinical trials in combination with the checkpoint inhibitors pembrolizumab (NCT04105335) and atezolizumab plus bevacizumab (an anti-VEGF antibody) (NCT05097911). A new generation of drug delivery systems is also currently being developed to selectively target cancer cells/stem cells and/or the tumor microenvironment [289,311,312]. In this system, anticancer drugs are not delivered passively to the tumor thanks to its high vascularity (enhanced permeability and retention effect), but are actively delivered to the tumor by smart nanoparticles (Smart NPs) that, unlike conventional nanoparticles, target cancer biomarkers for precise drug delivery [313]. Smart NP-based nano-formulations include the use of polymer-based nanocarriers (polymeric nanoparticles, dendrimers, and micelles), biomimetic-based nanocarriers (liposomes, protein nanoparticles, and cell membrane nanoparticles), inorganic nanocarriers (mesoporous silica nanoparticles, gold nanoparticles, iron oxide nanoparticles, quantum dots, and carbon nanotubes), and other advanced smart nanocarriers, such as black phosphorus and metal-organic frameworks. All these strategies are promising approaches to improve the therapeutic index of small molecule Wnt/β-catenin inhibitors for cancer management.

### 3.4. Patient Profiling

**Figure 1:**
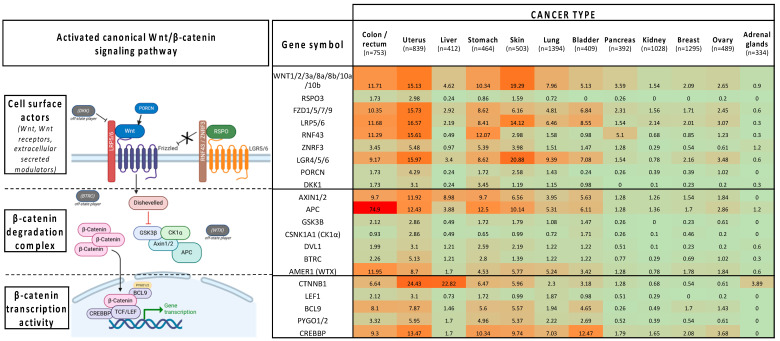
Mutation rates in key players of the canonical Wnt/B-catenin pathway in different cancer types (NIH GDC Data Portal release 40.0-March 2024): green (<20%), orange/red (>20%).

**Figure 2:**
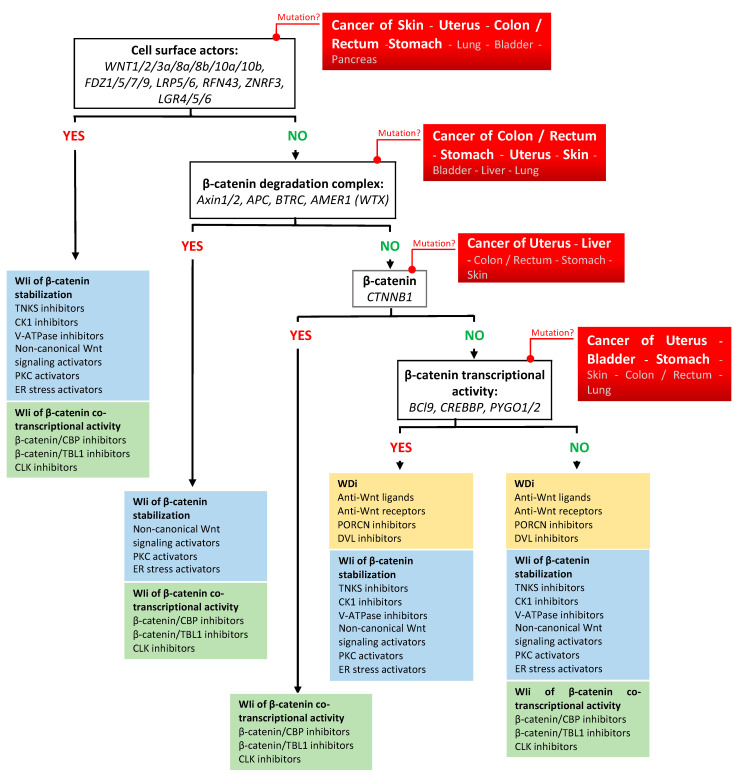
Flowchart for using Wnt/β-catenin inhibitors as anti-cancer treatments on the basis of the data presented in Figure 1 and Scheme 1, Scheme 2, Scheme 3 and Scheme 4.

As described for many anticancer agents, clinical trials on Wnt/β-catenin inhibitors showed limited benefits in terms of response rate, survival, and quality of life. However, they also provided evidence that targeting Wnt/β-catenin signaling is a relevant therapeutic option in several cancer types, highlighting the need of implementing tailored therapies that take into account the cancer type and where genetic mutations act along the Wnt/β-catenin signaling cascade (Figure 1 and Figure 2) [314]. Illustrative examples include the promising overall response rate observed in patients with advanced gastrointestinal cancer harboring *RPSO3* or *RFN43* gene alterations following treatment with the PORCNi CGX1321 combined with the checkpoint inhibitor pembrolizumab. Moreover, preliminary data indicate a potential clinical benefit in patients with MSS colorectal cancer treated with the PORCNi ETC-159 [137,138]. In addition, the DVL inhibitor sulindac prevents malignant transformation in patients with FAP when combined with eflornithine [156]. Some Wnt/β-catenin inhibitors also showed promising anticancer activity when used as monotherapy in specific contexts. Specifically, the TNKSi nesuparid (JPI-547) showed efficacy in patients with breast cancer harboring BRCA/HRR mutations [169]. Moreover, patients who respond to E7449 (another TNKSi) might be identified thanks to the drug response predictor 2X-121 [166]. Similar to other targeted therapies, key challenges for optimizing the chances of success with Wnt/β-catenin inhibitors lie in the implementation of sensitive and robust methods to identify a comprehensive set of biomarkers that will guide clinicians in personalizing cancer management in function of the patient’s cancer profile. For cancer profiling, multiplexing technologies (e.g., high throughput “omics”: genomics, transcriptomics, proteomics, metabolomics, radiomics, and immunomics) could be combined with functional tests and with digital technologies (e.g., machine learning and artificial intelligence) [315,316,317,318,319,320,321,322,323,324,325,326,327]. Given the scientific and technological progress in the era of personalized medicine, genomic profiling should become the standard in clinical practice and cancer biomarkers should be routinely used in clinical trials for patient recruitment and follow-up in the near future [328,329]. This will undoubtedly help to promote the use of Wnt/β-catenin inhibitors as anticancer agents for some clinical indications.

## 4. Conclusions

In the past decades, cancer research generated a plethora of data, methods, and chemical compounds that demonstrate the relevance of targeting Wnt/β-catenin signaling for cancer management. Preclinical and clinical studies highlighted promising anticancer effects of some Wnt/β-catenin inhibitors for specific clinical indications and demonstrated substantial benefit when Wnt/β-catenin inhibitors are combined with other agents, such as anti-PD-1/L1 antibodies [330]. As observed for many anticancer drugs, the current main challenges are to improve the effectiveness and safety of these compounds for routine clinical practice. The Wnt/β-catenin pathway proved difficult to target clinically, and no inhibitors of this signaling pathway were approved by the FDA or EMA, mainly due to the significant risk of serious adverse effects. These limitations are partly linked to the screening strategy of many of these inhibitors, i.e., the use of the TOPFlash luciferase reporter assay, an artificial in vitro system that is far short of reflecting the complexity of the biological mechanisms controlled by Wnt/β-catenin signaling. Advances in drug design and formulation, preclinical and clinical research, patient profiling, and digital technologies should significantly contribute to address these major issues in the near future.

## Supplementary Materials

The following supporting information can be downloaded at: https://www.mdpi.com/article/10.3390/ph17070949/s1, Table S1: Few highlights from Wnt discovery to the design of Wnt/β-catenin inhibitors as anticancer agents.

## References

[1] Van Ooyen A., Nusse R. (1984). Structure and Nucleotide Sequence of the Putative Mammary Oncogene Int-1; Proviral Insertions Leave the Protein-Encoding Domain Intact. Cell.

[2] Bittner J.J. (1936). Some Possible Effects of Nursing on the Mammary Gland Tumor Incidence in Mice. Science.

[3] Lyons M.J., Moore D.H. (1962). Purification of the Mouse Mammary Tumour Virus. Nature.

[4] Nusse R., Varmus H.E. (1982). Many Tumors Induced by the Mouse Mammary Tumor Virus Contain a Provirus Integrated in the Same Region of the Host Genome. Cell.

[5] Basham K.J., Rodriguez S., Turcu A.F., Lerario A.M., Logan C.Y., Rysztak M.R., Gomez-Sanchez C.E., Breault D.T., Koo B.-K., Clevers H. (2019). A ZNRF3-Dependent Wnt/β-Catenin Signaling Gradient Is Required for Adrenal Homeostasis. Genes Dev..

[6] van Ooyen A., Kwee V., Nusse R. (1985). The Nucleotide Sequence of the Human Int-1 Mammary Oncogene; Evolutionary Conservation of Coding and Non-Coding Sequences. EMBO J..

[7] Fung Y.-K.T., Shackleford G.M., Brown A.M.C., Sanders G.S., Varmus H.E. (1985). Nucleotide Sequence and Expression In Vitro of cDNA Derived from mRNA of *int*-1, a Provirally Activated Mouse Mammary Oncogene. Mol. Cell. Biol..

[8] Tsukamoto A.S., Grosschedl R., Guzman R.C., Parslow T., Varmus H.E. (1988). Expression of the Int-1 Gene in Transgenic Mice Is Associated with Mammary Gland Hyperplasia and Adenocarcinomas in Male and Female Mice. Cell.

[9] Thomas K.R., Capecchi M.R. (1990). Targeted Disruption of the Murine Int-1 Proto-Oncogene Resulting in Severe Abnormalities in Midbrain and Cerebellar Development. Nature.

[10] Gavin B.J., McMahon J.A., McMahon A.P. (1990). Expression of Multiple Novel Wnt-1/Int-1-Related Genes during Fetal and Adult Mouse Development. Genes Dev..

[11] Yost C., Torres M., Miller J.R., Huang E., Kimelman D., Moon R.T. (1996). The Axis-Inducing Activity, Stability, and Subcellular Distribution of Beta-Catenin Is Regulated in Xenopus Embryos by Glycogen Synthase Kinase 3. Genes Dev..

[12] Brunner E., Peter O., Schweizer L., Basler K. (1997). Pangolinencodes a Lef-1 Homologue That Acts Downstream of Armadillo to Transduce the Wingless Signal in Drosophila. Nature.

[13] Clevers H., Van De Wetering M. (1997). TCF/LEF Factors Earn Their Wings. Trends Genet..

[14] Hamada F., Tomoyasu Y., Takatsu Y., Nakamura M., Nagai S., Suzuki A., Fujita F., Shibuya H., Toyoshima K., Ueno N. (1999). Negative Regulation of Wingless Signaling by D-Axin, a Drosophila Homolog of Axin. Science.

[15] Otero L., Lacunza E., Vasquez V., Arbelaez V., Cardier F., González F. (2019). Variations in AXIN2 Predict Risk and Prognosis of Colorectal Cancer. BDJ Open.

[16] Groden J., Thliveris A., Samowitz W., Carlson M., Gelbert L., Albertsen H., Joslyn G., Stevens J., Spirio L., Robertson M. (1991). Identification and Characterization of the Familial Adenomatous Polyposis Coli Gene. Cell.

[17] Kinzler K.W., Nilbert M.C., Su L.-K., Vogelstein B., Bryan T.M., Levy D.B., Smith K.J., Preisinger A.C., Hedge P., McKechnie D. (1991). Identification of FAP Locus Genes from Chromosome 5q21. Science.

[18] Miyoshi Y., Ando H., Nagase H., Nishisho I., Horii A., Miki Y., Mori T., Utsunomiya J., Baba S., Petersen G. (1992). Germ-Line Mutations of the APC Gene in 53 Familial Adenomatous Polyposis Patients. Proc. Natl. Acad. Sci. USA.

[19] Miyoshi Y., Iwao K., Nagasawa Y., Aihara T., Sasaki Y., Imaoka S., Murata M., Shimano T., Nakamura Y. (1998). Activation of the Beta-Catenin Gene in Primary Hepatocellular Carcinomas by Somatic Alterations Involving Exon 3. Cancer Res..

[20] Kennerdell J.R., Carthew R.W. (1998). Use of dsRNA-Mediated Genetic Interference to Demonstrate That Frizzled and Frizzled 2 Act in the Wingless Pathway. Cell.

[21] Liu W., Dong X., Mai M., Seelan R.S., Taniguchi K., Krishnadath K.K., Halling K.C., Cunningham J.M., Boardman L.A., Qian C. (2000). Mutations in AXIN2 Cause Colorectal Cancer with Defective Mismatch Repair by Activating Beta-Catenin/TCF Signalling. Nat. Genet..

[22] Lammi L., Arte S., Somer M., Järvinen H., Lahermo P., Thesleff I., Pirinen S., Nieminen P. (2004). Mutations in AXIN2 Cause Familial Tooth Agenesis and Predispose to Colorectal Cancer. Am. J. Human. Genet..

[23] Park J.Y., Park W.S., Nam S.W., Kim S.Y., Lee S.H., Yoo N.J., Lee J.Y., Park C.K. (2005). Mutations of Beta-Catenin and AXIN I Genes Are a Late Event in Human Hepatocellular Carcinogenesis. Liver Int..

[24] Seshagiri S., Stawiski E.W., Durinck S., Modrusan Z., Storm E.E., Conboy C.B., Chaudhuri S., Guan Y., Janakiraman V., Jaiswal B.S. (2012). Recurrent R-Spondin Fusions in Colon Cancer. Nature.

[25] Jiang X., Hao H.-X., Growney J.D., Woolfenden S., Bottiglio C., Ng N., Lu B., Hsieh M.H., Bagdasarian L., Meyer R. (2013). Inactivating Mutations of RNF43 Confer Wnt Dependency in Pancreatic Ductal Adenocarcinoma. Proc. Natl. Acad. Sci. USA.

[26] Sekine S., Yamashita S., Tanabe T., Hashimoto T., Yoshida H., Taniguchi H., Kojima M., Shinmura K., Saito Y., Hiraoka N. (2016). Frequent *PTPRK-RSPO3* Fusions and *RNF43* Mutations in Colorectal Traditional Serrated Adenoma: *RSPO3* Fusions and *RNF43* Mutations in Colorectal TSA. J. Pathol..

[27] Neumeyer V., Grandl M., Dietl A., Brutau-Abia A., Allgäuer M., Kalali B., Zhang Y., Pan K.-F., Steiger K., Vieth M. (2019). Loss of Endogenous RNF43 Function Enhances Proliferation and Tumour Growth of Intestinal and Gastric Cells. Carcinogenesis.

[28] Qin K., Yu M., Fan J., Wang H., Zhao P., Zhao G., Zeng W., Chen C., Wang Y., Wang A. (2024). Canonical and Noncanonical Wnt Signaling: Multilayered Mediators, Signaling Mechanisms and Major Signaling Crosstalk. Genes Dis..

[29] Pandur P., Maurus D., Kühl M. (2002). Increasingly Complex: New Players Enter the Wnt Signaling Network. BioEssays.

[30] Park W.-J., Liu J., Adler P.N. (1994). The Frizzled Gene of Drosophila Encodes a Membrane Protein with an Odd Number of Transmembrane Domains. Mech. Dev..

[31] Vinson C.R., Conover S., Adler P.N. (1989). A Drosophila Tissue Polarity Locus Encodes a Protein Containing Seven Potential Transmembrane Domains. Nature.

[32] Dijksterhuis J.P., Baljinnyam B., Stanger K., Sercan H.O., Ji Y., Andres O., Rubin J.S., Hannoush R.N., Schulte G. (2015). Systematic Mapping of WNT-FZD Protein Interactions Reveals Functional Selectivity by Distinct WNT-FZD Pairs. J. Biol. Chem..

[33] Rulifson E.J., Wu C.H., Nusse R. (2000). Pathway Specificity by the Bifunctional Receptor Frizzled Is Determined by Affinity for Wingless. Mol. Cell.

[34] Janda C.Y., Waghray D., Levin A.M., Thomas C., Garcia K.C. (2012). Structural Basis of Wnt Recognition by Frizzled. Science.

[35] Tsutsumi N., Hwang S., Waghray D., Hansen S., Jude K.M., Wang N., Miao Y., Glassman C.R., Caveney N.A., Janda C.Y. (2023). Structure of the Wnt-Frizzled-LRP6 Initiation Complex Reveals the Basis for Coreceptor Discrimination. Proc. Natl. Acad. Sci. USA.

[36] Van Neerven S.M., De Groot N.E., Nijman L.E., Scicluna B.P., Van Driel M.S., Lecca M.C., Warmerdam D.O., Kakkar V., Moreno L.F., Vieira Braga F.A. (2021). Apc-Mutant Cells Act as Supercompetitors in Intestinal Tumour Initiation. Nature.

[37] Verkaar F., Zaman G.J.R. (2010). A Model for Signaling Specificity of Wnt/Frizzled Combinations through Co-receptor Recruitment. FEBS Lett..

[38] Kikuchi A., Yamamoto H., Kishida S. (2007). Multiplicity of the Interactions of Wnt Proteins and Their Receptors. Cell. Signal..

[39] Mao J., Wang J., Liu B., Pan W., Farr G.H., Flynn C., Yuan H., Takada S., Kimelman D., Li L. (2001). Low-Density Lipoprotein Receptor-Related Protein-5 Binds to Axin and Regulates the Canonical Wnt Signaling Pathway. Mol. Cell.

[40] Cadigan K.M., Liu Y.I. (2006). Wnt Signaling: Complexity at the Surface. J. Cell Sci..

[41] Billiard J., Way D.S., Seestaller-Wehr L.M., Moran R.A., Mangine A., Bodine P.V.N. (2005). The Orphan Receptor Tyrosine Kinase Ror2 Modulates Canonical Wnt Signaling in Osteoblastic Cells. Mol. Endocrinol..

[42] Patthy L. (2000). The WIF Module. Trends Biochem. Sci..

[43] Menck K., Heinrichs S., Baden C., Bleckmann A. (2021). The WNT/ROR Pathway in Cancer: From Signaling to Therapeutic Intervention. Cells.

[44] Shi F., Mendrola J.M., Sheetz J.B., Wu N., Sommer A., Speer K.F., Noordermeer J.N., Kan Z.-Y., Perry K., Englander S.W. (2021). ROR and RYK Extracellular Region Structures Suggest That Receptor Tyrosine Kinases Have Distinct WNT-Recognition Modes. Cell Rep..

[45] Ren Q., Chen J., Liu Y. (2021). LRP5 and LRP6 in Wnt Signaling: Similarity and Divergence. Front. Cell Dev. Biol..

[46] Green J., Nusse R., Van Amerongen R. (2014). The Role of Ryk and Ror Receptor Tyrosine Kinases in Wnt Signal Transduction. Cold Spring Harb. Perspect. Biol..

[47] Bafico A., Liu G., Yaniv A., Gazit A., Aaronson S.A. (2001). Novel Mechanism of Wnt Signalling Inhibition Mediated by Dickkopf-1 Interaction with LRP6/Arrow. Nat. Cell Biol..

[48] Mao B., Wu W., Li Y., Hoppe D., Stannek P., Glinka A., Niehrs C. (2001). LDL-Receptor-Related Protein 6 Is a Receptor for Dickkopf Proteins. Nature.

[49] Semënov M.V., Tamai K., Brott B.K., Kühl M., Sokol S., He X. (2001). Head Inducer Dickkopf-1 Is a Ligand for Wnt Coreceptor LRP6. Curr. Biol..

[50] Oishi I., Suzuki H., Onishi N., Takada R., Kani S., Ohkawara B., Koshida I., Suzuki K., Yamada G., Schwabe G.C. (2003). The Receptor Tyrosine Kinase Ror2 Is Involved in Non-Canonical Wnt5a/JNK Signalling Pathway. Genes Cells.

[51] Yoda A., Oishi I., Minami Y. (2003). Expression and Function of the Ror-Family Receptor Tyrosine Kinases during Development: Lessons from Genetic Analyses of Nematodes, Mice, and Humans. J. Recept. Signal Transduct. Res..

[52] Yoshikawa S., McKinnon R.D., Kokel M., Thomas J.B. (2003). Wnt-Mediated Axon Guidance via the Drosophila Derailed Receptor. Nature.

[53] Zeng X., Tamai K., Doble B., Li S., Huang H., Habas R., Okamura H., Woodgett J., He X. (2005). A Dual-Kinase Mechanism for Wnt Co-Receptor Phosphorylation and Activation. Nature.

[54] Li V.S.W., Ng S.S., Boersema P.J., Low T.Y., Karthaus W.R., Gerlach J.P., Mohammed S., Heck A.J.R., Maurice M.M., Mahmoudi T. (2012). Wnt Signaling through Inhibition of β-Catenin Degradation in an Intact Axin1 Complex. Cell.

[55] Aberle H., Bauer A., Stappert J., Kispert A., Kemler R. (1997). β-Catenin Is a Target for the Ubiquitin–Proteasome Pathway. EMBO J..

[56] Orford K., Crockett C., Jensen J.P., Weissman A.M., Byers S.W. (1997). Serine Phosphorylation-Regulated Ubiquitination and Degradation of β-Catenin. J. Biol. Chem..

[57] Schaefer K.N., Bonello T.T., Zhang S., Williams C.E., Roberts D.M., McKay D.J., Peifer M. (2018). Supramolecular Assembly of the Beta-Catenin Destruction Complex and the Effect of Wnt Signaling on Its Localization, Molecular Size, and Activity in Vivo. PLoS Genet..

[58] Munemitsu S., Albert I., Souza B., Rubinfeld B., Polakis P. (1995). Regulation of Intracellular Beta-Catenin Levels by the Adenomatous Polyposis Coli (APC) Tumor-Suppressor Protein. Proc. Natl. Acad. Sci. USA.

[59] Behrens J., Jerchow B.-A., Würtele M., Grimm J., Asbrand C., Wirtz R., Kühl M., Wedlich D., Birchmeier W. (1998). Functional Interaction of an Axin Homolog, Conductin, with β-Catenin, APC, and GSK3β. Science.

[60] Hart M.J., De Los Santos R., Albert I.N., Rubinfeld B., Polakis P. (1998). Downregulation of β-Catenin by Human Axin and Its Association with the APC Tumor Suppressor, β-Catenin and GSK3β. Curr. Biol..

[61] Kishida S., Yamamoto H., Ikeda S., Kishida M., Sakamoto I., Koyama S., Kikuchi A. (1998). Axin, a Negative Regulator of the Wnt Signaling Pathway, Directly Interacts with Adenomatous Polyposis Coli and Regulates the Stabilization of β-Catenin. J. Biol. Chem..

[62] Nakamura T., Hamada F., Ishidate T., Anai K., Kawahara K., Toyoshima K., Akiyama T. (1998). Axin, an Inhibitor of the Wnt Signalling Pathway, Interacts with Β-catenin, GSK-3β and APC and Reduces the Β-catenin Level. Genes Cells.

[63] Spink K.E., Polakis P., Weis W.I. (2000). Structural Basis of the Axin–Adenomatous Polyposis Coli Interaction. EMBO J..

[64] Amit S., Hatzubai A., Birman Y., Andersen J.S., Ben-Shushan E., Mann M., Ben-Neriah Y., Alkalay I. (2002). Axin-Mediated CKI Phosphorylation of β-Catenin at Ser 45: A Molecular Switch for the Wnt Pathway. Genes Dev..

[65] Liu C., Li Y., Semenov M., Han C., Baeg G.H., Tan Y., Zhang Z., Lin X., He X. (2002). Control of Beta-Catenin Phosphorylation/Degradation by a Dual-Kinase Mechanism. Cell.

[66] Lee E., Salic A., Krüger R., Heinrich R., Kirschner M.W. (2003). The Roles of APC and Axin Derived from Experimental and Theoretical Analysis of the Wnt Pathway. PLoS Biol..

[67] Roberts D.M., Pronobis M.I., Poulton J.S., Waldmann J.D., Stephenson E.M., Hanna S., Peifer M. (2011). Deconstructing the SScatenin Destruction Complex: Mechanistic Roles for the Tumor Suppressor APC in Regulating Wnt Signaling. Mol. Biol. Cell.

[68] Su Y., Fu C., Ishikawa S., Stella A., Kojima M., Shitoh K., Schreiber E.M., Day B.W., Liu B. (2008). APC Is Essential for Targeting Phosphorylated β-Catenin to the SCFβ-TrCP Ubiquitin Ligase. Mol. Cell.

[69] Ha N.-C., Tonozuka T., Stamos J.L., Choi H.-J., Weis W.I. (2004). Mechanism of Phosphorylation-Dependent Binding of APC to β-Catenin and Its Role in β-Catenin Degradation. Mol. Cell.

[70] Tauriello D.V.F., Jordens I., Kirchner K., Slootstra J.W., Kruitwagen T., Bouwman B.A.M., Noutsou M., Rüdiger S.G.D., Schwamborn K., Schambony A. (2012). Wnt/β-Catenin Signaling Requires Interaction of the Dishevelled DEP Domain and C Terminus with a Discontinuous Motif in Frizzled. Proc. Natl. Acad. Sci. USA.

[71] Finch P.W., He X., Kelley M.J., Uren A., Schaudies R.P., Popescu N.C., Rudikoff S., Aaronson S.A., Varmus H.E., Rubin J.S. (1997). Purification and Molecular Cloning of a Secreted, Frizzled-Related Antagonist of Wnt Action. Proc. Natl. Acad. Sci. USA.

[72] Leyns L., Bouwmeester T., Kim S.H., Piccolo S., De Robertis E.M. (1997). Frzb-1 Is a Secreted Antagonist of Wnt Signaling Expressed in the Spemann Organizer. Cell.

[73] Rattner A., Hsieh J.C., Smallwood P.M., Gilbert D.J., Copeland N.G., Jenkins N.A., Nathans J. (1997). A Family of Secreted Proteins Contains Homology to the Cysteine-Rich Ligand-Binding Domain of Frizzled Receptors. Proc. Natl. Acad. Sci. USA.

[74] Cliffe A., Hamada F., Bienz M. (2003). A Role of Dishevelled in Relocating Axin to the Plasma Membrane during Wingless Signaling. Curr. Biol..

[75] Schwarz-Romond T., Metcalfe C., Bienz M. (2007). Dynamic Recruitment of Axin by Dishevelled Protein Assemblies. J. Cell Sci..

[76] MacDonald B.T., Tamai K., He X. (2009). Wnt/Beta-Catenin Signaling: Components, Mechanisms, and Diseases. Dev. Cell.

[77] The Wnt Home Page. https://web.stanford.edu/group/nusselab/cgi-bin/wnt/target_genes.

[78] Van Tienen L.M., Mieszczanek J., Fiedler M., Rutherford T.J., Bienz M. (2017). Constitutive Scaffolding of Multiple Wnt Enhanceosome Components by Legless/BCL9. eLife.

[79] Barker N. (2001). The Chromatin Remodelling Factor Brg-1 Interacts with Beta-Catenin to Promote Target Gene Activation. EMBO J..

[80] Townsley F.M., Thompson B., Bienz M. (2004). Pygopus Residues Required for Its Binding to Legless Are Critical for Transcription and Development. J. Biol. Chem..

[81] Hecht A., Vleminckx K., Stemmler M.P., van Roy F., Kemler R. (2000). The P300/CBP Acetyltransferases Function as Transcriptional Coactivators of Beta-Catenin in Vertebrates. EMBO J..

[82] Sansom O.J., Meniel V.S., Muncan V., Phesse T.J., Wilkins J.A., Reed K.R., Vass J.K., Athineos D., Clevers H., Clarke A.R. (2007). Myc Deletion Rescues Apc Deficiency in the Small Intestine. Nature.

[83] Ramakrishnan A.-B., Cadigan K.M. (2017). Wnt Target Genes and Where to Find Them. F1000Research.

[84] He T.C., Sparks A.B., Rago C., Hermeking H., Zawel L., da Costa L.T., Morin P.J., Vogelstein B., Kinzler K.W. (1998). Identification of C-MYC as a Target of the APC Pathway. Science.

[85] Jackstadt R., Hodder M.C., Sansom O.J. (2020). WNT and β-Catenin in Cancer: Genes and Therapy. Annu. Rev. Cancer Biol..

[86] Nusse R., Clevers H. (2017). Wnt/β-Catenin Signaling, Disease, and Emerging Therapeutic Modalities. Cell.

[87] Yu F., Yu C., Li F., Zuo Y., Wang Y., Yao L., Wu C., Wang C., Ye L. (2021). Wnt/β-Catenin Signaling in Cancers and Targeted Therapies. Sig. Transduct. Target. Ther..

[88] Krishnamurthy N., Kurzrock R. (2018). Targeting the Wnt/Beta-Catenin Pathway in Cancer: Update on Effectors and Inhibitors. Cancer Treat. Rev..

[89] Zhang Y., Wang X. (2020). Targeting the Wnt/β-Catenin Signaling Pathway in Cancer. J. Hematol. Oncol..

[90] Nie S., Wang Z., Moscoso-Castro M., D’Souza P., Lei C., Xu J., Gu J. (2020). Biology Drives the Discovery of Bispecific Antibodies as Innovative Therapeutics. Antib. Ther..

[91] Jimeno A., Gordon M., Chugh R., Messersmith W., Mendelson D., Dupont J., Stagg R., Kapoun A.M., Xu L., Uttamsingh S. (2017). A First-in-Human Phase I Study of the Anticancer Stem Cell Agent Ipafricept (OMP-54F28), a Decoy Receptor for Wnt Ligands, in Patients with Advanced Solid Tumors. Clin. Cancer Res..

[92] Dotan E., Cardin D.B., Lenz H.-J., Messersmith W., O’Neil B., Cohen S.J., Denlinger C.S., Shahda S., Astsaturov I., Kapoun A.M. (2020). Phase Ib Study of Wnt Inhibitor Ipafricept with Gemcitabine and Nab-Paclitaxel in Patients with Previously Untreated Stage IV Pancreatic Cancer. Clin. Cancer Res..

[93] Moore K.N., Gunderson C.C., Sabbatini P., McMeekin D.S., Mantia-Smaldone G., Burger R.A., Morgan M.A., Kapoun A.M., Brachmann R.K., Stagg R. (2019). A Phase 1b Dose Escalation Study of Ipafricept (OMP 54F28) in Combination with Paclitaxel and Carboplatin in Patients with Recurrent Platinum-Sensitive Ovarian Cancer. Gynecol. Oncol..

[94] Vlashi R., Zhang X., Wu M., Chen G. (2023). Wnt Signaling: Essential Roles in Osteoblast Differentiation, Bone Metabolism and Therapeutic Implications for Bone and Skeletal Disorders. Genes Dis..

[95] Fischer M.M., Cancilla B., Yeung V.P., Cattaruzza F., Chartier C., Murriel C.L., Cain J., Tam R., Cheng C.-Y., Evans J.W. (2017). WNT Antagonists Exhibit Unique Combinatorial Antitumor Activity with Taxanes by Potentiating Mitotic Cell Death. Sci. Adv..

[96] OncoMed P.I. OncoMed Pre-Announces 2014 Year-End Cash Balance and Provides 2015 Guidance. https://www.globenewswire.com/news-release/2015/01/12/696685/10115034/en/oncomed-pre-announces-2014-year-end-cash-balance-and-provides-2015-guidance.html.

[97] Smith D.C., Rosen L.S., Chugh R., Goldman J.W., Xu L., Kapoun A., Brachmann R.K., Dupont J., Stagg R.J., Tolcher A.W. (2013). First-in-Human Evaluation of the Human Monoclonal Antibody Vantictumab (OMP-18R5; Anti-Frizzled) Targeting the WNT Pathway in a Phase I Study for Patients with Advanced Solid Tumors. J. Clin. Oncol..

[98] Diamond J.R., Becerra C., Richards D., Mita A., Osborne C., O’Shaughnessy J., Zhang C., Henner R., Kapoun A.M., Xu L. (2020). Phase Ib Clinical Trial of the Anti-Frizzled Antibody Vantictumab (OMP-18R5) plus Paclitaxel in Patients with Locally Advanced or Metastatic HER2-Negative Breast Cancer. Breast Cancer Res. Treat..

[99] Davis S.L., Cardin D.B., Shahda S., Lenz H.-J., Dotan E., O’Neil B.H., Kapoun A.M., Stagg R.J., Berlin J., Messersmith W.A. (2020). A Phase 1b Dose Escalation Study of Wnt Pathway Inhibitor Vantictumab in Combination with Nab-Paclitaxel and Gemcitabine in Patients with Previously Untreated Metastatic Pancreatic Cancer. Investig. New Drugs.

[100] Giraudet A.-L., Cassier P.A., Iwao-Fukukawa C., Garin G., Badel J.-N., Kryza D., Chabaud S., Gilles-Afchain L., Clapisson G., Desuzinges C. (2018). A First-in-Human Study Investigating Biodistribution, Safety and Recommended Dose of a New Radiolabeled MAb Targeting FZD10 in Metastatic Synovial Sarcoma Patients. BMC Cancer.

[101] Inglis D.J., Licari J., Georgiou K.R., Wittwer N.L., Hamilton R.W., Beaumont D.M., Scherer M.A., Lavranos T.C. (2018). Abstract 3910: Characterization of BNC101 a Human Specific Monoclonal Antibody Targeting the GPCR LGR5: First-in-Human Evidence of Target Engagement. Cancer Res..

[102] Carmon K.S., Lin Q., Gong X., Thomas A., Liu Q. (2012). LGR5 Interacts and Cointernalizes with Wnt Receptors To Modulate Wnt/β-Catenin Signaling. Mol. Cell. Biol..

[103] Xu L., Lin W., Wen L., Li G. (2019). Lgr5 in Cancer Biology: Functional Identification of Lgr5 in Cancer Progression and Potential Opportunities for Novel Therapy. Stem Cell Res. Ther..

[104] Élez E., Lenz H.-J., De Jonge M., Yaeger R., Doi T., Pronk L., Teufel M., Marzin K., Tabernero J. (2022). Abstract CT514: A Phase I, Open-Label, Dose-Escalation Study Investigating a Low-Density Lipoprotein Receptor-Related Protein (LRP) 5/6 Inhibitor, BI 905677, in Patients with Advanced Solid Tumors. Cancer Res..

[105] Jiang H., Zhang Z., Yu Y., Chu H.Y., Yu S., Yao S., Zhang G., Zhang B.-T. (2022). Drug Discovery of DKK1 Inhibitors. Front. Pharmacol..

[106] Ahn V.E., Chu M.L.-H., Choi H.-J., Tran D., Abo A., Weis W.I. (2011). Structural Basis of Wnt Signaling Inhibition by Dickkopf Binding to LRP5/6. Dev. Cell.

[107] Wall J.A., Klempner S.J., Arend R.C. (2020). The Anti-DKK1 Antibody DKN-01 as an Immunomodulatory Combination Partner for the Treatment of Cancer. Expert. Opin. Investig. Drugs.

[108] Goyal L., Sirard C., Schrag M., Kagey M.H., Eads J.R., Stein S., El-Khoueiry A.B., Manji G.A., Abrams T.A., Khorana A.A. (2020). Phase I and Biomarker Study of the Wnt Pathway Modulator DKN-01 in Combination with Gemcitabine/Cisplatin in Advanced Biliary Tract Cancer. Clin. Cancer Res..

[109] Turkes F.S., Crux R., Tran A., Cartwright E., Rana I., Johnston E., Dunlop A., Thomas J., Smith A., Smyth E. (2022). 1253P Safety and Efficacy of Wnt Inhibition with a DKK1 Inhibitor, DKN-01, in Combination with Atezolizumab in Patients with Advanced Oesophagogastric Adenocarcinoma: Phase IIa Results of the WAKING Trial. Ann. Oncol..

[110] Heath D.J., Chantry A.D., Buckle C.H., Coulton L., Shaughnessy J.D., Evans H.R., Snowden J.A., Stover D.R., Vanderkerken K., Croucher P.I. (2009). Inhibiting Dickkopf-1 (Dkk1) Removes Suppression of Bone Formation and Prevents the Development of Osteolytic Bone Disease in Multiple Myeloma. J. Bone Miner. Res..

[111] Fulciniti M., Tassone P., Hideshima T., Vallet S., Nanjappa P., Ettenberg S.A., Shen Z., Patel N., Tai Y., Chauhan D. (2009). Anti-DKK1 mAb (BHQ880) as a Potential Therapeutic Agent for Multiple Myeloma. Blood.

[112] Iyer S.P., Beck J.T., Stewart A.K., Shah J., Kelly K.R., Isaacs R., Bilic S., Sen S., Munshi N.C. (2014). A Phase IB Multicentre Dose-determination Study of BHQ 880 in Combination with Anti-myeloma Therapy and Zoledronic Acid in Patients with Relapsed or Refractory Multiple Myeloma and Prior Skeletal-related Events. Br. J. Haematol..

[113] Fischer M.M., Yeung V.P., Cattaruzza F., Hussein R., Yen W.-C., Murriel C., Evans J.W., O’Young G., Brunner A.L., Wang M. (2017). RSPO3 Antagonism Inhibits Growth and Tumorigenicity in Colorectal Tumors Harboring Common Wnt Pathway Mutations. Sci. Rep..

[114] Flanagan D.J., Pentinmikko N., Luopajärvi K., Willis N.J., Gilroy K., Raven A.P., Mcgarry L., Englund J.I., Webb A.T., Scharaw S. (2021). NOTUM from Apc-Mutant Cells Biases Clonal Competition to Initiate Cancer. Nature.

[115] Hao H.-X., Jiang X., Cong F. (2016). Control of Wnt Receptor Turnover by R-Spondin-ZNRF3/RNF43 Signaling Module and Its Dysregulation in Cancer. Cancers.

[116] Park H.-B., Kim J.-W., Baek K.-H. (2020). Regulation of Wnt Signaling through Ubiquitination and Deubiquitination in Cancers. Int. J. Mol. Sci..

[117] Yan K.S., Janda C.Y., Chang J., Zheng G.X.Y., Larkin K.A., Luca V.C., Chia L.A., Mah A.T., Han A., Terry J.M. (2017). Non-Equivalence of Wnt and R-Spondin Ligands during Lgr5+ Intestinal Stem-Cell Self-Renewal. Nature.

[118] Gong X., Yi J., Carmon K.S., Crumbley C.A., Xiong W., Thomas A., Fan X., Guo S., An Z., Chang J.T. (2015). Aberrant RSPO3-LGR4 Signaling in Keap1-Deficient Lung Adenocarcinomas Promotes Tumor Aggressiveness. Oncogene.

[119] Bendell J., Eckhardt G.S., Hochster H.S., Morris V.K., Strickler J., Kapoun A.M., Wang M., Xu L., McGuire K., Dupont J. (2016). Initial Results from a Phase 1a/b Study of OMP-131R10, a First-in-Class Anti-RSPO3 Antibody, in Advanced Solid Tumors and Previously Treated Metastatic Colorectal Cancer (CRC). Eur. J. Cancer.

[120] Zhang M., Haughey M., Wang N.-Y., Blease K., Kapoun A.M., Couto S., Belka I., Hoey T., Groza M., Hartke J. (2020). Targeting the Wnt Signaling Pathway through R-Spondin 3 Identifies an Anti-Fibrosis Treatment Strategy for Multiple Organs. PLoS ONE.

[121] Herzog B.H., Baer J.M., Borcherding N., Kingston N.L., Belle J.I., Knolhoff B.L., Hogg G.D., Ahmad F., Kang L.-I., Petrone J. (2023). Tumor-Associated Fibrosis Impairs Immune Surveillance and Response to Immune Checkpoint Blockade in Non–Small Cell Lung Cancer. Sci. Transl. Med..

[122] Piersma B., Hayward M.-K., Weaver V.M. (2020). Fibrosis and Cancer: A Strained Relationship. Biochim. Biophys. Acta (BBA)—Rev. Cancer.

[123] Wu B., Sodji Q.H., Oyelere A.K. (2022). Inflammation, Fibrosis and Cancer: Mechanisms, Therapeutic Options and Challenges. Cancers.

[124] Bayle E.D., Svensson F., Atkinson B.N., Steadman D., Willis N.J., Woodward H.L., Whiting P., Vincent J.-P., Fish P.V. (2021). Carboxylesterase Notum Is a Druggable Target to Modulate Wnt Signaling. J. Med. Chem..

[125] Kakugawa S., Langton P.F., Zebisch M., Howell S.A., Chang T.-H., Liu Y., Feizi T., Bineva G., O’Reilly N., Snijders A.P. (2015). Notum Deacylates Wnt Proteins to Suppress Signalling Activity. Nature.

[126] Shah K., Panchal S., Patel B. (2021). Porcupine Inhibitors: Novel and Emerging Anti-Cancer Therapeutics Targeting the Wnt Signaling Pathway. Pharmacol. Res..

[127] Kadowaki T., Wilder E., Klingensmith J., Zachary K., Perrimon N. (1996). The Segment Polarity Gene Porcupine Encodes a Putative Multitransmembrane Protein Involved in Wingless Processing. Genes Dev..

[128] Van Den Heuvel M., Harryman-Samos C., Klingensmith J., Perrimon N., Nusse R. (1993). Mutations in the Segment Polarity Genes Wingless and Porcupine Impair Secretion of the Wingless Protein. EMBO J..

[129] Li C., Cao J., Zhang N., Tu M., Xu F., Wei S., Chen X., Xu Y. (2018). Identification of RSPO2 Fusion Mutations and Target Therapy Using a Porcupine Inhibitor. Sci. Rep..

[130] Liu J., Pan S., Hsieh M.H., Ng N., Sun F., Wang T., Kasibhatla S., Schuller A.G., Li A.G., Cheng D. (2013). Targeting Wnt-Driven Cancer through the Inhibition of Porcupine by LGK974. Proc. Natl. Acad. Sci. USA.

[131] Koo B.-K., van Es J.H., van den Born M., Clevers H. (2015). Porcupine Inhibitor Suppresses Paracrine Wnt-Driven Growth of Rnf43;Znrf3-Mutant Neoplasia. Proc. Natl. Acad. Sci. USA.

[132] Madan B., Ke Z., Harmston N., Ho S.Y., Frois A.O., Alam J., Jeyaraj D.A., Pendharkar V., Ghosh K., Virshup I.H. (2016). Wnt Addiction of Genetically Defined Cancers Reversed by PORCN Inhibition. Oncogene.

[133] Bhamra I., Adams N., Armer R., Bingham M., McKeever H., Phillips C., Thompson B., Woodcock S. (2017). Novel Porcupine (PORCN) Inhibitor RXC004: Evaluation in Models of RNF43 Loss of Function Cancers. J. Clin. Oncol..

[134] Picco G., Petti C., Centonze A., Torchiaro E., Crisafulli G., Novara L., Acquaviva A., Bardelli A., Medico E. (2017). Loss of AXIN1 Drives Acquired Resistance to WNT Pathway Blockade in Colorectal Cancer Cells Carrying RSPO 3 Fusions. EMBO Mol. Med..

[135] Rodon J., Argilés G., Connolly R.M., Vaishampayan U., De Jonge M., Garralda E., Giannakis M., Smith D.C., Dobson J.R., McLaughlin M.E. (2021). Phase 1 Study of Single-Agent WNT974, a First-in-Class Porcupine Inhibitor, in Patients with Advanced Solid Tumours. Br. J. Cancer.

[136] Tan D., Ng M., Subbiah V., Messersmith W., Teneggi V., Diermayr V., Ethirajulu K., Yeo P., Gan B.H., Lee L.H. (2018). Phase I Extension Study of ETC-159 an Oral PORCN Inhibitor Administered with Bone Protective Treatment, in Patients with Advanced Solid Tumours. Ann. Oncol..

[137] Tan D.S.P., Ng M.C.H., Subbiah V., Messersmith W.A., Strickler J.H., Diermayr V., Cometa J., Blanchard S., Nellore R., Pendharkar V. (2023). A Phase 1B Dose Escalation Study of ETC-159 in Combination with Pembrolizumab in Advanced or Metastatic Solid Tumours. J. Clin. Oncol..

[138] Giannakis M., Le D.T., Pishvaian M.J., Weinberg B.A., Papadopoulos K.P., Shen L., Gong J., Li J., Strickler J.H., Zhou A. (2023). Phase 1 Study of WNT Pathway Porcupine Inhibitor CGX1321 and Phase 1b Study of CGX1321 + Pembrolizumab (Pembro) in Patients (Pts) with Advanced Gastrointestinal (GI) Tumors. J. Clin. Oncol..

[139] Pharmaceutical Technology CGX-1321 by Curegenix for Pancreatic Cancer: Likelihood of Approval. https://www.pharmaceutical-technology.com/data-insights/cgx-1321-curegenix-pancreatic-cancer-likelihood-of-approval/.

[140] Kopetz S., Morris V.K., O’Neil B., Bridgewater J.A., Graham J., Parkes E.E., Saunders M.P., Asken E., Goodwin L., Phillips C. (2022). A Multi-Arm, Phase 2, Open-Label Study to Assess the Efficacy of RXC004 as Monotherapy and in Combination with Nivolumab in Patients with Ring Finger Protein 43 (RNF43) or R-Spondin (RSPO) Aberrated, Metastatic, Microsatellite Stable Colorectal Cancer Following Standard Treatments. J. Clin. Oncol..

[141] Yang Q., Qin T., An T., Wu H., Xu G., Xiang J., Lei K., Zhang S., Xia J., Su G. (2023). Novel PORCN Inhibitor WHN-88 Targets Wnt/β-Catenin Pathway and Prevents the Growth of Wnt-Driven Cancers. Eur. J. Pharmacol..

[142] Chen W., Chen M., Barak L.S. (2010). Development of Small Molecules Targeting the Wnt Pathway for the Treatment of Colon Cancer: A High-Throughput Screening Approach. Am. J. Physiol. -Gastrointest. Liver Physiol..

[143] Chen M., Wang J., Lu J., Bond M.C., Ren X.-R., Lyerly H.K., Barak L.S., Chen W. (2009). The Anti-Helminthic Niclosamide Inhibits Wnt/Frizzled1 Signaling. Biochemistry.

[144] Osada T., Chen M., Yang X.Y., Spasojevic I., Vandeusen J.B., Hsu D., Clary B.M., Clay T.M., Chen W., Morse M.A. (2011). Antihelminth Compound Niclosamide Downregulates Wnt Signaling and Elicits Antitumor Responses in Tumors with Activating APC Mutations. Cancer Res..

[145] Huang S., Chen J., Tian R., Wang J., Xie C., Gao H., Shan Y., Hong J., Zhang Z., Xu M. (2018). Down-regulation of Dishevelled-2 Inhibits Cell Proliferation and Invasion in Hepatoblastoma. Pediatr. Blood Cancer.

[146] Zeyada M.S., Abdel-Rahman N., El-Karef A., Yahia S., El-Sherbiny I.M., Eissa L.A. (2020). Niclosamide-Loaded Polymeric Micelles Ameliorate Hepatocellular Carcinoma in Vivo through Targeting Wnt and Notch Pathways. Life Sci..

[147] Burock S., Daum S., Keilholz U., Neumann K., Walther W., Stein U. (2018). Phase II Trial to Investigate the Safety and Efficacy of Orally Applied Niclosamide in Patients with Metachronous or Sychronous Metastases of a Colorectal Cancer Progressing after Therapy: The NIKOLO Trial. BMC Cancer.

[148] Schweizer M.T., Haugk K., McKiernan J.S., Gulati R., Cheng H.H., Maes J.L., Dumpit R.F., Nelson P.S., Montgomery B., McCune J.S. (2018). A Phase I Study of Niclosamide in Combination with Enzalutamide in Men with Castration-Resistant Prostate Cancer. PLoS ONE.

[149] Lee H., Wang N.X., Shi D., Zheng J.J. (2009). Sulindac Inhibits Canonical Wnt Signaling by Blocking the PDZ Domain of the Protein Dishevelled. Angew. Chem. Int. Ed..

[150] Tutter A.V., Fryer C.J., Jones K.A. (2001). Chromatin-Specific Regulation of LEF-1–β-Catenin Transcription Activation and Inhibition in Vitro. Genes Dev..

[151] Limburg P.J., Mandrekar S.J., Aubry M.C., Ziegler K.L.A., Zhang J., Yi J.E., Henry M., Tazelaar H.D., Lam S., McWilliams A. (2013). Randomized Phase II Trial of Sulindac for Lung Cancer Chemoprevention. Lung Cancer.

[152] Masters G.A., Li S., Dowlati A., Madajewicz S., Langer C., Schiller J., Johnson D. (2006). A Phase II Trial of Carboplatin and Gemcitabine with Exisulind (IND #65,056) in Patients with Advanced Non-Small Cell Lung Cancer: An Eastern Cooperative Oncology Group Study (E1501). J. Thorac. Oncol..

[153] Dawson N.A., Halabi S., Ou S.-S., Biggs D.D., Kessinger A., Vogelzang N., Clamon G.H., Nanus D.M., Kelly W.K., Small E.J. (2008). A Phase II Study of Estramustine, Docetaxel, and Exisulind in Patients with Hormone-Refractory Prostate Cancer: Results of Cancer and Leukemia Group B Trial 90004. Clin. Genitourin. Cancer.

[154] Govindan R., Wang X., Baggstrom M.Q., Burdette-Radoux S., Hodgson L., Vokes E.E., Green M.R. (2009). A Phase II Study of Carboplatin, Etoposide, and Exisulind in Patients with Extensive Small Cell Lung Cancer: CALGB 30104. J. Thorac. Oncol..

[155] Thompson P.A., Wertheim B.C., Zell J.A., Chen W.-P., McLaren C.E., LaFleur B.J., Meyskens F.L., Gerner E.W. (2010). Levels of Rectal Mucosal Polyamines and Prostaglandin E2 Predict Ability of DFMO and Sulindac to Prevent Colorectal Adenoma. Gastroenterology.

[156] Burke C.A., Dekker E., Lynch P., Samadder N.J., Balaguer F., Hüneburg R., Burn J., Castells A., Gallinger S., Lim R. (2020). Eflornithine plus Sulindac for Prevention of Progression in Familial Adenomatous Polyposis. N. Engl. J. Med..

[157] Samadder N.J., Kuwada S.K., Boucher K.M., Byrne K., Kanth P., Samowitz W., Jones D., Tavtigian S.V., Westover M., Berry T. (2018). Association of Sulindac and Erlotinib vs Placebo With Colorectal Neoplasia in Familial Adenomatous Polyposis: Secondary Analysis of a Randomized Clinical Trial. JAMA Oncol..

[158] Samadder N.J., Neklason D.W., Boucher K.M., Byrne K.R., Kanth P., Samowitz W., Jones D., Tavtigian S.V., Done M.W., Berry T. (2016). Effect of Sulindac and Erlotinib vs Placebo on Duodenal Neoplasia in Familial Adenomatous Polyposis: A Randomized Clinical Trial. J. Am. Med. Assoc..

[159] Martinez J.A., Wertheim B.C., Roe D.J., Chalasani P., Cohen J., Baer L., Chow H.-H.S., Stopeck A.T., Thompson P.A. (2022). Sulindac Improves Stiffness and Quality of Life in Women Taking Aromatase Inhibitors for Breast Cancer. Breast Cancer Res. Treat..

[160] Thompson P.A., Huang C., Yang J., Wertheim B.C., Roe D., Zhang X., Ding J., Chalasani P., Preece C., Martinez J. (2021). Sulindac, a Nonselective NSAID, Reduces Breast Density in Postmenopausal Women with Breast Cancer Treated with Aromatase Inhibitors. Clin. Cancer Res..

[161] Croy H.E., Fuller C.N., Giannotti J., Robinson P., Foley A.V.A., Yamulla R.J., Cosgriff S., Greaves B.D., Von Kleeck R.A., An H.H. (2016). The Poly(ADP-Ribose) Polymerase Enzyme Tankyrase Antagonizes Activity of the β-Catenin Destruction Complex through ADP-Ribosylation of Axin and APC2. J. Biol. Chem..

[162] McGonigle S., Chen Z., Wu J., Chang P., Kolber-Simonds D., Ackermann K., Twine N.C., Shie J.-L., Miu J.T., Huang K.-C. (2015). E7449: A Dual Inhibitor of PARP1/2 and Tankyrase1/2 Inhibits Growth of DNA Repair Deficient Tumors and Antagonizes Wnt Signaling. Oncotarget.

[163] Huang S.-M.A., Mishina Y.M., Liu S., Cheung A., Stegmeier F., Michaud G.A., Charlat O., Wiellette E., Zhang Y., Wiessner S. (2009). Tankyrase Inhibition Stabilizes Axin and Antagonizes Wnt Signalling. Nature.

[164] Mariotti L., Templeton C.M., Ranes M., Paracuellos P., Cronin N., Beuron F., Morris E., Guettler S. (2016). Tankyrase Requires SAM Domain-Dependent Polymerization to Support Wnt-β-Catenin Signaling. Mol. Cell.

[165] Tanaka N., Mashima T., Mizutani A., Sato A., Aoyama A., Gong B., Yoshida H., Muramatsu Y., Nakata K., Matsuura M. (2017). *APC* Mutations as a Potential Biomarker for Sensitivity to Tankyrase Inhibitors in Colorectal Cancer. Mol. Cancer Ther..

[166] Plummer R., Dua D., Cresti N., Drew Y., Stephens P., Foegh M., Knudsen S., Sachdev P., Mistry B.M., Dixit V. (2020). First-in-Human Study of the PARP/Tankyrase Inhibitor E7449 in Patients with Advanced Solid Tumours and Evaluation of a Novel Drug-Response Predictor. Br. J. Cancer.

[167] Kang M.S., Katuwal N., Ghosh M., Jeong Y.K., Deok Hong S., Park S.M., Kim J., Cha H., Cheon B., Kim S.-G. (2023). Abstract 4485: JPI-547, a Novel Dual Inhibitor of PARP 1/2 and Tankyrase 1/2 Overcomes Olaparib Resistance in BRCA 1/2 Mutant Ovary and Breast Cancer Preclinical Model. Cancer Res..

[168] Oh K.-S., Nam A.-R., Bang J.-H., Jeong Y., Choo S.Y., Kim H.J., Lee S.I., Kim J.-M., Yoon J., Kim T.-Y. (2023). Abstract 4496: JPI-547, a Dual Inhibitor of PARP/Tankyrase, Shows Promising Antitumor Activity against Pancreatic Cancers with Homologous Recombination Repair Deficiency or Wnt-Addiction. Cancer Res..

[169] Im S.-A., Lee S., Lee K.W., Lee Y., Sohn J., Kim J.H., Im Y.-H., Park K.H., Oh D.-Y., Kim M.H. (2021). A Phase I Dose-Escalation and Expansion Study of JPI-547, a Dual Inhibitor of PARP/Tankyrase in Patients with Advanced Solid Tumors. J. Clin. Oncol..

[170] Pharmaceutical Technology Nesuparib by Onconic Therapeutics for Epithelial Ovarian Cancer: Likelihood of Approval. https://www.pharmaceutical-technology.com/data-insights/nesuparib-onconic-therapeutics-epithelial-ovarian-cancer-likelihood-of-approval/.

[171] Shen C., Li B., Astudillo L., Deutscher M.P., Cobb M.H., Capobianco A.J., Lee E., Robbins D.J. (2019). The CK1α Activator Pyrvinium Enhances the Catalytic Efficiency (*k*_cat_/*K*_m_) of CK1α. Biochemistry.

[172] Saraswati S., Alfaro M.P., Thorne C.A., Atkinson J., Lee E., Young P.P. (2010). Pyrvinium, a Potent Small Molecule Wnt Inhibitor, Promotes Wound Repair and Post-MI Cardiac Remodeling. PLoS ONE.

[173] Thorne C.A., Hanson A.J., Schneider J., Tahinci E., Orton D., Cselenyi C.S., Jernigan K.K., Meyers K.C., Hang B.I., Waterson A.G. (2010). Small-Molecule Inhibition of Wnt Signaling through Activation of Casein Kinase 1α. Nat. Chem. Biol..

[174] Schultz C.W., Nevler A. (2022). Pyrvinium Pamoate: Past, Present, and Future as an Anti-Cancer Drug. Biomedicines.

[175] Ponzini F.M., Schultz C.W., Leiby B.E., Cannaday S., Yeo T., Posey J., Bowne W.B., Yeo C., Brody J.R., Lavu H. (2023). Repurposing the FDA-Approved Anthelmintic Pyrvinium Pamoate for Pancreatic Cancer Treatment: Study Protocol for a Phase I Clinical Trial in Early-Stage Pancreatic Ductal Adenocarcinoma. BMJ Open.

[176] Jung Y., Stratton S.A., Lee S.H., Kim M., Jun S., Zhang J., Zheng B., Cervantes C.L., Cha J., Barton M.C. (2021). TMEM9-v-ATPase Activates Wnt/β-Catenin Signaling Via APC Lysosomal Degradation for Liver Regeneration and Tumorigenesis. Hepatology.

[177] Datta S., Choudhury D., Das A., Mukherjee D.D., Dasgupta M., Bandopadhyay S., Chakrabarti G. (2019). Autophagy Inhibition with Chloroquine Reverts Paclitaxel Resistance and Attenuates Metastatic Potential in Human Nonsmall Lung Adenocarcinoma A549 Cells via ROS Mediated Modulation of β-Catenin Pathway. Apoptosis.

[178] Verbaanderd C., Maes H., Schaaf M.B., Sukhatme V.P., Pantziarka P., Sukhatme V., Agostinis P., Bouche G. (2017). Repurposing Drugs in Oncology (ReDO)—Chloroquine and Hydroxychloroquine as Anti-Cancer Agents. Ecancermedicalscience.

[179] Lee J.M., Kim I.S., Kim H., Lee J.S., Kim K., Yim H.Y., Jeong J., Kim J.H., Kim J.-Y., Lee H. (2010). RORα Attenuates Wnt/β-Catenin Signaling by PKCα-Dependent Phosphorylation in Colon Cancer. Mol. Cell.

[180] Osman J., Bellamkonda K., Liu Q., Andersson T., Sjölander A. (2019). The WNT5A Agonist Foxy5 Reduces the Number of Colonic Cancer Stem Cells in a Xenograft Mouse Model of Human Colonic Cancer. Anticancer. Res..

[181] Prasad C.P., Manchanda M., Mohapatra P., Andersson T. (2018). WNT5A as a Therapeutic Target in Breast Cancer. Cancer Metastasis Rev..

[182] Canesin G., Evans-Axelsson S., Hellsten R., Krzyzanowska A., Prasad C.P., Bjartell A., Andersson T. (2017). Treatment with the WNT5A-Mimicking Peptide Foxy-5 Effectively Reduces the Metastatic Spread of WNT5A-Low Prostate Cancer Cells in an Orthotopic Mouse Model. PLoS ONE.

[183] Säfholm A., Tuomela J., Rosenkvist J., Dejmek J., Härkönen P., Andersson T. (2008). The Wnt-5a–Derived Hexapeptide Foxy-5 Inhibits Breast Cancer Metastasis In Vivo by Targeting Cell Motility. Clin. Cancer Res..

[184] Vermorken J., Cervantes A., Morsing P., Johansson K., Andersson T., Roest N.L., Gullbo J., Salazar R. (2019). A Randomized, Multicenter, Open-Label Controlled Phase 2 Trial of Foxy-5 as Neoadjuvant Therapy in Patients with WNT5A Negative Colon Cancer. Ann. Oncol..

[185] Yadav V., Islam R., Tuli H.S. (2023). Patent Landscape Highlighting Double-Edged Scaffold of a WNT5A-Agonizing Peptide, Foxy5. Pharm. Pat. Anal..

[186] Kawano T., Inokuchi J., Eto M., Murata M., Kang J.-H. (2021). Activators and Inhibitors of Protein Kinase C (PKC): Their Applications in Clinical Trials. Pharmaceutics.

[187] Antal C.E., Hudson A.M., Kang E., Zanca C., Wirth C., Stephenson N.L., Trotter E.W., Gallegos L.L., Miller C.J., Furnari F.B. (2015). Cancer-Associated Protein Kinase C Mutations Reveal Kinase’s Role as Tumor Suppressor. Cell.

[188] Shah K., Kazi J.U. (2022). Phosphorylation-Dependent Regulation of WNT/Beta-Catenin Signaling. Front. Oncol..

[189] Dupasquier S., Blache P., Picque Lasorsa L., Zhao H., Abraham J.-D., Haigh J.J., Ychou M., Prévostel C. (2019). Modulating PKCα Activity to Target Wnt/β-Catenin Signaling in Colon Cancer. Cancers.

[190] Hernández-Maqueda J.G., Luna-Ulloa L.B., Santoyo-Ramos P., Castañeda-Patlán M.C., Robles-Flores M. (2013). Protein Kinase C Delta Negatively Modulates Canonical Wnt Pathway and Cell Proliferation in Colon Tumor Cell Lines. PLoS ONE.

[191] Gwak J., Cho M., Gong S.-J., Won J., Kim D.-E., Kim E.-Y., Lee S.S., Kim M., Kim T.K., Shin J.-G. (2006). Protein-Kinase-C-Mediated β-Catenin Phosphorylation Negatively Regulates the Wnt/β-Catenin Pathway. J. Cell Sci..

[192] Llado V., Nakanishi Y., Duran A., Reina-Campos M., Shelton P.M., Linares J.F., Yajima T., Campos A., Aza-Blanc P., Leitges M. (2015). Repression of Intestinal Stem Cell Function and Tumorigenesis through Direct Phosphorylation of β-Catenin and Yap by PKCζ. Cell Rep..

[193] Velin M., Cardot-Leccia N., Cathelineau A., Duteil L., Queille-Roussel C., Passeron T., Bahadoran P. (2023). Efficacy and Safety of 0.05% Ingenol Mebutate in the Treatment of Basal Cell Carcinoma: A Prospective Study. Skin. Health Dis..

[194] Siller G., Rosen R., Freeman M., Welburn P., Katsamas J., Ogbourne S.M. (2010). PEP005 (Ingenol Mebutate) Gel for the Topical Treatment of Superficial Basal Cell Carcinoma: Results of a Randomized Phase IIa Trial. Aust. J. Dermatol..

[195] Ramsay J.R., Suhrbier A., Aylward J.H., Ogbourne S., Cozzi S.-J., Poulsen M.G., Baumann K.C., Welburn P., Redlich G.L., Parsons P.G. (2011). The Sap from Euphorbia Peplus Is Effective against Human Nonmelanoma Skin Cancers: Euphorbia Peplus Sap Is Effective against Skin Cancers. Br. J. Dermatol..

[196] Lebwohl M., Swanson N., Anderson L.L., Melgaard A., Xu Z., Berman B. (2012). Ingenol Mebutate Gel for Actinic Keratosis. N. Engl. J. Med..

[197] Lebwohl M., Shumack S., Gold L.S., Melgaard A., Larsson T., Tyring S.K. (2013). Long-Term Follow-up Study of Ingenol Mebutate Gel for the Treatment of Actinic Keratoses. JAMA Dermatol..

[198] Fidler B., Goldberg T. (2014). Ingenol Mebutate Gel (Picato): A Novel Agent for the Treatment of Actinic Keratoses. Pharm. Ther..

[199] Anderson L., Jarratt M., Schmieder G., Shumack S., Katsamas J., Welburn P. (2014). Tolerability and Pharmacokinetics of Ingenol Mebutate 0.05% Gel Applied to Treatment Areas up to 100cm(2) on the Forearm(s) of Patients with Actinic Keratosis. J. Clin. Aesthet. Dermatol..

[200] Berman B., Goldenberg G., Hanke C.W., Tyring S.K., Werschler W.P., Knudsen K.M., Goncalves J., Larsson T., Skov T., Swanson N. (2014). Efficacy and Safety of Ingenol Mebutate 0.015% Gel 3 Weeks after Cryosurgery of Actinic Keratosis: 11-Week Results. J. Drugs Dermatol..

[201] Berman B., Goldenberg G., Hanke C.W., Tyring S.K., Werschler W.P., Knudsen K.M., Larsson T., Swanson N. (2014). Efficacy and Safety of Ingenol Mebutate 0.015% Gel after Cryosurgery of Actinic Keratosis: 12-Month Results. J. Drugs Dermatol..

[202] Pellacani G., Peris K., Guillen C., Clonier F., Larsson T., Venkata R., Puig S. (2015). A Randomized Trial Comparing Simultaneous vs. Sequential Field Treatment of Actinic Keratosis with Ingenol Mebutate on Two Separate Areas of the Head and Body. J. Eur. Acad. Dermatol. Venereol..

[203] Samorano L.P., Torezan L.A., Sanches J.A. (2015). Evaluation of the Tolerability and Safety of a 0.015% Ingenol Mebutate Gel Compared to 5% 5-fluorouracil Cream for the Treatment of Facial Actinic Keratosis: A Prospective Randomized Trial. Acad. Dermatol. Venereol..

[204] Emmert S., Haenssle H.A., Zibert J.R., Schön M., Hald A., Hansen M.H., Litman T., Schön M.P. (2016). Tumor-Preferential Induction of Immune Responses and Epidermal Cell Death in Actinic Keratoses by Ingenol Mebutate. PLoS ONE.

[205] Schopf R.E. (2016). Ingenol Mebutate Gel Is Effective against Anogenital Warts—A Case Series in 17 Patients. Acad. Dermatol. Venereol..

[206] Wu D.C., Guiha I., Goldman M.P. (2017). A Prospective Pilot Clinical Trial to Evaluate the Efficacy and Safety of Topical Therapy with Ingenol Mebutate Gel 0.015% for Actinic Keratosis on an Expanded Area of the Chest. J. Clin. Aesthet. Dermatol..

[207] Stockfleth E., Harwood C.A., Serra-Guillén C., Larsson T., Østerdal M.L., Skov T. (2018). Phase IV Head-to-Head Randomized Controlled Trial Comparing Ingenol Mebutate 0·015% Gel with Diclofenac Sodium 3% Gel for the Treatment of Actinic Keratosis on the Face or Scalp. Br. J. Dermatol..

[208] Jansen M.H.E., Kessels J.P.H.M., Nelemans P.J., Kouloubis N., Arits A.H.M.M., Van Pelt H.P.A., Quaedvlieg P.J.F., Essers B.A.B., Steijlen P.M., Kelleners-Smeets N.W.J. (2019). Randomized Trial of Four Treatment Approaches for Actinic Keratosis. N. Engl. J. Med..

[209] Jansen M.H.E., Kessels J.P.H.M., Merks I., Nelemans P.J., Kelleners-Smeets N.W.J., Mosterd K., Essers B.A.B. (2020). A Trial-based Cost-effectiveness Analysis of Topical 5-fluorouracil vs. Imiquimod vs. Ingenol Mebutate vs. Methyl Aminolaevulinate Conventional Photodynamic Therapy for the Treatment of Actinic Keratosis in the Head and Neck Area Performed in the Netherlands. Br. J. Dermatol..

[210] Ahmady S., Jansen M.H.E., Nelemans P.J., Essers B.A.B., Kessels J.P.H.M., Kelleners-Smeets N.W.J., Mosterd K. (2021). The Effect of Four Approaches to Treat Actinic Keratosis on the Health-Related QOL, as Assessed by the Skindex-29 and Actinic Keratosis QOL. J. Investig. Dermatol..

[211] Ahmady S., Jansen M.H.E., Nelemans P.J., Kessels J.P.H.M., Arits A.H.M.M., De Rooij M.J.M., Essers B.A.B., Quaedvlieg P.J.F., Kelleners-Smeets N.W.J., Mosterd K. (2022). Risk of Invasive Cutaneous Squamous Cell Carcinoma After Different Treatments for Actinic Keratosis: A Secondary Analysis of a Randomized Clinical Trial. JAMA Dermatol..

[212] Rosen R.H., Gupta A.K., Tyring S.K. (2012). Dual Mechanism of Action of Ingenol Mebutate Gel for Topical Treatment of Actinic Keratoses: Rapid Lesion Necrosis Followed by Lesion-Specific Immune Response. J. Am. Acad. Dermatol..

[213] Han Z.T., Zhu X.X., Yang R.Y., Sun J.Z., Tian G.F., Liu X.J., Cao G.S., Newmark H.L., Conney A.H., Chang R.L. (1998). Effect of Intravenous Infusions of 12-O-Tetradecanoylphorbol-13-Acetate (TPA) in Patients with Myelocytic Leukemia: Preliminary Studies on Therapeutic Efficacy and Toxicity. Proc. Natl. Acad. Sci. USA.

[214] Fang B., Song Y., Han Z., Wei X., Lin Q., Zhu X., Yang R., Sun J., Tian G., Liu X. (2007). Synergistic Interactions between 12-0-Tetradecanoylphorbol-13-Acetate (TPA) and Imatinib in Patients with Chronic Myeloid Leukemia in Blastic Phase That Is Resistant to Standard-Dose Imatinib. Leuk. Res..

[215] Fürstenberger G., Berry D.L., Sorg B., Marks F. (1981). Skin Tumor Promotion by Phorbol Esters Is a Two-Stage Process. Proc. Natl. Acad. Sci. USA.

[216] Wang W., Cho U., Yoo A., Jung C.-L., Kim B., Kim H., Lee J., Jo H., Han Y., Song M.-H. (2022). Wnt/β-Catenin Inhibition by CWP232291 as a Novel Therapeutic Strategy in Ovarian Cancer. Front. Oncol..

[217] Pak S., Park S., Kim Y., Park J.-H., Park C.-H., Lee K.-J., Kim C., Ahn H. (2019). The Small Molecule WNT/β-Catenin Inhibitor CWP232291 Blocks the Growth of Castration-Resistant Prostate Cancer by Activating the Endoplasmic Reticulum Stress Pathway. J. Exp. Clin. Cancer Res..

[218] Park J.W., Um H., Yang H., Cha J.Y., Lee K.-J., Kim H.K. (2017). CWP232291, a Wnt/β-Catenin Inhibitor, to Suppress the Growth and Development of Gastrointestinal Cancers. J. Clin. Oncol..

[219] Cha J.Y., Jung J.-E., Lee K.-H., Briaud I., Tenzin F., Jung H.K., Pyon Y., Lee D., Chung J.U., Lee J.H. (2010). Anti-Tumor Activity of Novel Small Molecule Wnt Signaling Inhibitor, CWP232291, In Multiple Myeloma. Blood.

[220] Yoon S.-S., Manasanch E.E., Min C.K., Kim J.S., Hauptschein R.S., Choi J., Chun J.K. (2017). Novel Phase 1a/1b Dose-Finding Study Design of CWP232291 (CWP291) in Relapsed or Refractory Myeloma (MM). J. Clin. Oncol..

[221] Lee J.-H., Faderl S., Pagel J.M., Jung C.W., Yoon S.-S., Pardanani A.D., Becker P.S., Lee H., Choi J., Lee K. (2020). Phase 1 Study of CWP232291 in Patients with Relapsed or Refractory Acute Myeloid Leukemia and Myelodysplastic Syndrome. Blood Advances.

[222] Teo J.-L., Ma H., Nguyen C., Lam C., Kahn M. (2005). Specific Inhibition of CBP/Beta-Catenin Interaction Rescues Defects in Neuronal Differentiation Caused by a Presenilin-1 Mutation. Proc. Natl. Acad. Sci. USA.

[223] McMillan M., Kahn M. (2005). Investigating Wnt Signaling: A Chemogenomic Safari. Drug Discov. Today.

[224] Eguchi M., Nguyen C., Lee S.C., Kahn M. (2005). ICG-001, a Novel Small Molecule Regulator of TCF/Beta-Catenin Transcription. Med. Chem..

[225] Ma H., Nguyen C., Lee K.-S., Kahn M. (2005). Differential Roles for the Coactivators CBP and P300 on TCF/Beta-Catenin-Mediated Survivin Gene Expression. Oncogene.

[226] Gutova M., Hibbard J.C., Ma E., Natri H.M., Adhikarla V., Chimge N.-O., Qiu R., Nguyen C., Melendez E., Aguilar B. (2024). Targeting Wnt Signaling for Improved Glioma Immunotherapy. Front. Immunol..

[227] Islam M.S., Parish M., Brennan J.T., Winer B.L., Segars J.H. (2023). Targeting Fibrotic Signaling Pathways by EGCG as a Therapeutic Strategy for Uterine Fibroids. Sci. Rep..

[228] Okazaki H., Sato S., Koyama K., Morizumi S., Abe S., Azuma M., Chen Y., Goto H., Aono Y., Ogawa H. (2019). The Novel Inhibitor PRI-724 for Wnt/β-Catenin/CBP Signaling Ameliorates Bleomycin-Induced Pulmonary Fibrosis in Mice. Exp. Lung Res..

[229] Bae J.-S., Ryu G., Kim J.H., Kim E.H., Rhee Y.H., Chung Y.-J., Kim D.W., Lim S., Chung P.-S., Shin H.-W. (2020). Effects of Wnt Signaling on Epithelial to Mesenchymal Transition in Chronic Rhinosinusitis with Nasal Polyp. Thorax.

[230] Nishikawa K., Osawa Y., Kimura K. (2018). Wnt/β-Catenin Signaling as a Potential Target for the Treatment of Liver Cirrhosis Using Antifibrotic Drugs. Int. J. Mol. Sci..

[231] Kimura K., Ikoma A., Shibakawa M., Shimoda S., Harada K., Saio M., Imamura J., Osawa Y., Kimura M., Nishikawa K. (2017). Safety, Tolerability, and Preliminary Efficacy of the Anti-Fibrotic Small Molecule PRI-724, a CBP/β-Catenin Inhibitor, in Patients with Hepatitis C Virus-Related Cirrhosis: A Single-Center, Open-Label, Dose Escalation Phase 1 Trial. EBioMedicine.

[232] Kimura K., Kanto T., Shimoda S., Harada K., Kimura M., Nishikawa K., Imamura J., Ogawa E., Saio M., Ikura Y. (2022). Safety, Tolerability, and Anti-Fibrotic Efficacy of the CBP/β-Catenin Inhibitor PRI-724 in Patients with Hepatitis C and B Virus-Induced Liver Cirrhosis: An Investigator-Initiated, Open-Label, Non-Randomised, Multicentre, Phase 1/2a Study. EBioMedicine.

[233] El-Khoueiry A.B., Ning Y., Yang D., Cole S., Kahn M., Zoghbi M., Berg J., Fujimori M., Inada T., Kouji H. (2013). A Phase I First-in-Human Study of PRI-724 in Patients (Pts) with Advanced Solid Tumors. J. Clin. Oncol..

[234] Ko A.H., Chiorean E.G., Kwak E.L., Lenz H.-J., Nadler P.I., Wood D.L., Fujimori M., Inada T., Kouji H., McWilliams R.R. (2016). Final Results of a Phase Ib Dose-Escalation Study of PRI-724, a CBP/Beta-Catenin Modulator, plus Gemcitabine (GEM) in Patients with Advanced Pancreatic Adenocarcinoma (APC) as Second-Line Therapy after FOLFIRINOX or FOLFOX. J. Clin. Oncol..

[235] McWilliams R.R., Ko A.H., Chiorean E.G., Kwak E.L., Lenz H.-J., Nadler P.I., Wood D.L., Fujimori M., Morita K., Inada T. (2015). A Phase Ib Dose-Escalation Study of PRI-724, a CBP/Beta-Catenin Modulator, plus Gemcitabine (GEM) in Patients with Advanced Pancreatic Adenocarcinoma (APC) as Second-Line Therapy after FOLFIRINOX or FOLFOX. J. Clin. Oncol..

[236] Yamada K., Hori Y., Inoue S., Yamamoto Y., Iso K., Kamiyama H., Yamaguchi A., Kimura T., Uesugi M., Ito J. (2021). E7386, a Selective Inhibitor of the Interaction between β-Catenin and CBP, Exerts Antitumor Activity in Tumor Models with Activated Canonical Wnt Signaling. Cancer Res..

[237] Higuchi Y., Nguyen C., Chimge N.-O., Ouyang C., Teo J.-L., Kahn M. (2023). E7386 Is Not a Specific CBP/β-Catenin Antagonist. Curr. Mol. Pharmacol..

[238] Kondo S., Kawazoe A., Iwasa S., Yamamoto N., Ueda Y., Nagao S., Kimura T., Suzuki I., Hayata N., Tamai T. (2023). A Phase 1 Study of E7386, a CREB-Binding Protein (CBP)/β-Catenin Interaction Inhibitor, in Patients (Pts) with Advanced Solid Tumors Including Colorectal Cancer: Updated Dose-Escalation Part. J. Clin. Oncol..

[239] Ikeda M., Kato N., Kondo S., Inaba Y., Ueshima K., Sasaki M., Kanzaki H., Ida H., Imaoka H., Minami Y. (2023). A Phase 1b Study of E7386, a CREB-Binding Protein (CBP)/β-Catenin Interaction Inhibitor, in Combination with Lenvatinib in Patients with Advanced Hepatocellular Carcinoma. J. Clin. Oncol..

[240] Li J., Wang C.-Y. (2008). TBL1–TBLR1 and β-Catenin Recruit Each Other to Wnt Target-Gene Promoter for Transcription Activation and Oncogenesis. Nat. Cell Biol..

[241] Braggio D.A., de Faria F.C.C., Koller D., Jin F., Zewdu A., Lopez G., Batte K., Casadei L., Welliver M., Horrigan S.K. (2022). Preclinical Efficacy of the Wnt/β-Catenin Pathway Inhibitor BC2059 for the Treatment of Desmoid Tumors. PLoS ONE.

[242] Perissi V., Aggarwal A., Glass C.K., Rose D.W., Rosenfeld M.G. (2004). A Corepressor/Coactivator Exchange Complex Required for Transcriptional Activation by Nuclear Receptors and Other Regulated Transcription Factors. Cell.

[243] Nomura M., Rainusso N.C., Han R., Larson J., Shuck R.L., Kurenbekova L., Yustein J.T. (2018). Abstract 3186: Tegavivint Suppresses Progression and Metastasis of Osteosarcoma via Blockade of Wnt Signaling/ALDH1 Axis: Preclinical Study of a Novel Wnt/β-Catenin Pathway Inhibitor. Cancer Res..

[244] Dimitrova Y.N., Li J., Lee Y.-T., Rios-Esteves J., Friedman D.B., Choi H.-J., Weis W.I., Wang C.-Y., Chazin W.J. (2010). Direct Ubiquitination of β-Catenin by Siah-1 and Regulation by the Exchange Factor TBL1. J. Biol. Chem..

[245] Liu J., Stevens J., Rote C.A., Yost H.J., Hu Y., Neufeld K.L., White R.L., Matsunami N. (2001). Siah-1 Mediates a Novel β-Catenin Degradation Pathway Linking P53 to the Adenomatous Polyposis Coli Protein. Mol. Cell.

[246] Cranmer L.D., Abdul Razak A.R., Ratan R., Choy E., George S., Liebner D.A., Stenehjem D.D., Gounder M.M. (2022). Results of a Phase I Dose Escalation and Expansion Study of Tegavivint (BC2059), a First-in-Class TBL1 Inhibitor for Patients with Progressive, Unresectable Desmoid Tumor. J. Clin. Oncol..

[247] Tam B.Y., Chiu K., Chung H., Bossard C., Nguyen J.D., Creger E., Eastman B.W., Mak C.C., Ibanez M., Ghias A. (2020). The CLK Inhibitor SM08502 Induces Anti-Tumor Activity and Reduces Wnt Pathway Gene Expression in Gastrointestinal Cancer Models. Cancer Lett..

[248] Corr B.R., Moroney M.R., Woodruff E., Watson Z.L., Jordan K.R., Danhorn T., Bailey C., Wolsky R.J., Bitler B.G. (2023). Combination CDC-like Kinase Inhibition (CLK)/Dual-Specificity Tyrosine-Regulated Kinase (DYRK) and Taxane Therapy in CTNNB1 -Mutated Endometrial Cancer. bioRxiv.

[249] Deshmukh V., O’Green A.L., Bossard C., Seo T., Lamangan L., Ibanez M., Ghias A., Lai C., Do L., Cho S. (2019). Modulation of the Wnt Pathway through Inhibition of CLK2 and DYRK1A by Lorecivivint as a Novel, Potentially Disease-Modifying Approach for Knee Osteoarthritis Treatment. Osteoarthr. Cartil..

[250] Bossard C., Chiu K., Chung H., Nguyen J.D., Creger E., Eastman B., Mak C.C., Do L., Cho S., Kc S. (2019). Effects of SM08502, a Novel, Oral Small-Molecule Inhibitor of Wnt Pathway Signaling, on Gene Expression and Antitumor Activity in Colorectal Cancer (CRC) Models. J. Clin. Oncol..

[251] Deshmukh V., Seo T., O’Green A.L., Ibanez M., Hofilena B., Kc S., Stewart J., Dellamary L., Chiu K., Ghias A. (2021). SM04755, a Small-molecule Inhibitor of the Wnt Pathway, as a Potential Topical Treatment for Tendinopathy. J. Orthop. Res..

[252] Jantan I., Haque M.A., Arshad L., Harikrishnan H., Septama A.W., Mohamed-Hussein Z.-A. (2021). Dietary Polyphenols Suppress Chronic Inflammation by Modulation of Multiple Inflammation-Associated Cell Signaling Pathways. J. Nutr. Biochem..

[253] Scarpa E.-S., Ninfali P. (2015). Phytochemicals as Innovative Therapeutic Tools against Cancer Stem Cells. IJMS.

[254] Naujokat C., McKee D.L. (2021). The “Big Five” Phytochemicals Targeting Cancer Stem Cells: Curcumin, EGCG, Sulforaphane, Resveratrol and Genistein. Curr. Med. Chem..

[255] Liao W., Zhang L., Chen X., Xiang J., Zheng Q., Chen N., Zhao M., Zhang G., Xiao X., Zhou G. (2023). Targeting Cancer Stem Cells and Signalling Pathways through Phytochemicals: A Promising Approach against Colorectal Cancer. Phytomedicine.

[256] Tafrihi M., Nakhaei Sistani R. (2017). E-Cadherin/β-Catenin Complex: A Target for Anticancer and Antimetastasis Plants/Plant-Derived Compounds. Nutr. Cancer.

[257] Avila-Carrasco L., Majano P., Sánchez-Toméro J.A., Selgas R., López-Cabrera M., Aguilera A., González Mateo G. (2019). Natural Plants Compounds as Modulators of Epithelial-to-Mesenchymal Transition. Front. Pharmacol..

[258] Motallebi M., Bhia M., Rajani H.F., Bhia I., Tabarraei H., Mohammadkhani N., Pereira-Silva M., Kasaii M.S., Nouri-Majd S., Mueller A.-L. (2022). Naringenin: A Potential Flavonoid Phytochemical for Cancer Therapy. Life Sci..

[259] Morin P.J., Sparks A.B., Korinek V., Barker N., Clevers H., Vogelstein B., Kinzler K.W. (1997). Activation of Beta-Catenin-Tcf Signaling in Colon Cancer by Mutations in Beta-Catenin or APC. Science.

[260] Koo B.-K., Spit M., Jordens I., Low T.Y., Stange D.E., van de Wetering M., van Es J.H., Mohammed S., Heck A.J.R., Maurice M.M. (2012). Tumour Suppressor RNF43 Is a Stem-Cell E3 Ligase That Induces Endocytosis of Wnt Receptors. Nature.

[261] Preisler L., Ben-Yosef D., Mayshar Y. (2019). Adenomatous Polyposis Coli as a Major Regulator of Human Embryonic Stem Cells Self-Renewal. Stem Cells.

[262] Akhondzadeh S. (2016). The Importance of Clinical Trials in Drug Development. Avicenna J. Med. Biotechnol..

[263] Li B., Liang J., Lu F., Zeng G., Zhang J., Ma Y., Liu P., Wang Q., Zhou Q., Chen L. (2020). Discovery of Novel Inhibitor for WNT/β-Catenin Pathway by Tankyrase 1/2 Structure-Based Virtual Screening. Molecules.

[264] Low J.-L., Du W., Gocha T., Oguz G., Zhang X., Chen M.W., Masirevic S., Yim D.G.R., Tan I.B.H., Ramasamy A. (2021). Molecular Docking-Aided Identification of Small Molecule Inhibitors Targeting β-Catenin-TCF4 Interaction. iScience.

[265] Yan M., Li G., An J. (2017). Discovery of Small Molecule Inhibitors of the Wnt/β-Catenin Signaling Pathway by Targeting β-Catenin/Tcf4 Interactions. Exp. Biol. Med..

[266] Wang L., Song Y., Wang H., Zhang X., Wang M., He J., Li S., Zhang L., Li K., Cao L. (2023). Advances of Artificial Intelligence in Anti-Cancer Drug Design: A Review of the Past Decade. Pharmaceuticals.

[267] Ren J., Wang B., Wu Q., Wang G. (2022). Combination of Niclosamide and Current Therapies to Overcome Resistance for Cancer: New Frontiers for an Old Drug. Biomed. Pharmacother..

[268] Lehár J., Krueger A.S., Avery W., Heilbut A.M., Johansen L.M., Price E.R., Rickles R.J., Short Iii G.F., Staunton J.E., Jin X. (2009). Synergistic Drug Combinations Tend to Improve Therapeutically Relevant Selectivity. Nat. Biotechnol..

[269] Borisy A.A., Elliott P.J., Hurst N.W., Lee M.S., Lehár J., Price E.R., Serbedzija G., Zimmermann G.R., Foley M.A., Stockwell B.R. (2003). Systematic Discovery of Multicomponent Therapeutics. Proc. Natl. Acad. Sci. USA.

[270] Duarte D., Vale N. (2022). Evaluation of Synergism in Drug Combinations and Reference Models for Future Orientations in Oncology. Curr. Res. Pharmacol. Drug Discov..

[271] Tallarida R.J. (2011). Quantitative Methods for Assessing Drug Synergism. Genes Cancer.

[272] Sharma P. (2016). Immune Checkpoint Therapy and the Search for Predictive Biomarkers. Cancer J..

[273] Pardoll D.M. (2012). The Blockade of Immune Checkpoints in Cancer Immunotherapy. Nat. Rev. Cancer.

[274] Spranger S., Gajewski T.F. (2015). A New Paradigm for Tumor Immune Escape: β-Catenin-Driven Immune Exclusion. J. Immunother. Cancer.

[275] Biopahrma PEG FDA Approved Antibody-Drug Conjugates (ADCs) by 2024. https://www.biochempeg.com/article/74.html.

[276] Do M., Wu C.C.N., Sonavane P.R., Juarez E.F., Adams S.R., Ross J., Rodriguez Y Baena A., Patel C., Mesirov J.P., Carson D.A. (2022). A FZD7-Specific Antibody–Drug Conjugate Induces Ovarian Tumor Regression in Preclinical Models. Mol. Cancer Ther..

[277] Katoh M. (2017). Antibody-Drug Conjugate Targeting Protein Tyrosine Kinase 7, a Receptor Tyrosine Kinase-like Molecule Involved in WNT and Vascular Endothelial Growth Factor Signaling: Effects on Cancer Stem Cells, Tumor Microenvironment and Whole-Body Homeostasis. Ann. Transl. Med..

[278] Damelin M., Bankovich A., Bernstein J., Lucas J., Chen L., Williams S., Park A., Aguilar J., Ernstoff E., Charati M. (2017). A PTK7-Targeted Antibody-Drug Conjugate Reduces Tumor-Initiating Cells and Induces Sustained Tumor Regressions. Sci. Transl. Med..

[279] Berger H., Breuer M., Peradziryi H., Podleschny M., Jacob R., Borchers A. (2017). PTK7 Localization and Protein Stability Is Affected by Canonical Wnt Ligands. J. Cell Sci..

[280] Martinez S., Scerbo P., Giordano M., Daulat A.M., Lhoumeau A.-C., Thomé V., Kodjabachian L., Borg J.-P. (2015). The PTK7 and ROR2 Protein Receptors Interact in the Vertebrate WNT/Planar Cell Polarity (PCP) Pathway. J. Biol. Chem..

[281] Maitland M.L., Sachdev J.C., Sharma M.R., Moreno V., Boni V., Kummar S., Stringer-Reasor E., Lakhani N., Moreau A.R., Xuan D. (2021). First-in-Human Study of PF-06647020 (Cofetuzumab Pelidotin), an Antibody–Drug Conjugate Targeting Protein Tyrosine Kinase 7, in Advanced Solid Tumors. Clin. Cancer Res..

[282] Hirsch D., Ried T. (2016). Targeting Colorectal Cancer (Stem-like) Cells Using LGR5 Directed Antibody Drug Conjugates. Ann. Transl. Med..

[283] Junttila M.R., Mao W., Wang X., Wang B.-E., Pham T., Flygare J., Yu S.-F., Yee S., Goldenberg D., Fields C. (2015). Targeting LGR5 ^+^ Cells with an Antibody-Drug Conjugate for the Treatment of Colon Cancer. Sci. Transl. Med..

[284] Pan J., Li N., Renn A., Zhu H., Chen L., Shen M., Hall M.D., Qian M., Pastan I., Ho M. (2022). GPC1-Targeted Immunotoxins Inhibit Pancreatic Tumor Growth in Mice via Depletion of Short-Lived GPC1 and Downregulation of Wnt Signaling. Mol. Cancer Ther..

[285] Cherradi S., Garambois V., Marines J., Andrade A.F., Fauvre A., Morand O., Fargal M., Mancouri F., Ayrolles-Torro A., Vezzo-Vié N. (2023). Improving the Response to Oxaliplatin by Targeting Chemotherapy-Induced CLDN1 in Resistant Metastatic Colorectal Cancer Cells. Cell Biosci..

[286] Cui J., Park S., Yu W., Carmon K., Liu Q.J. (2021). Drug Conjugates of Antagonistic RSPO4 Mutant For Simultaneous Targeting of LGR4/5/6 for Cancer Treatment. J. Med. Chem..

[287] Rajabi A., Nejati M., Homayoonfal M., Arj A., Razavi Z.S., Ostadian A., Mohammadzadeh B., Vosough M., Karimi M., Rahimian N. (2024). Doxorubicin-Loaded Zymosan Nanoparticles: Synergistic Cytotoxicity and Modulation of Apoptosis and Wnt/β-Catenin Signaling Pathway in C26 Colorectal Cancer Cells. Int. J. Biol. Macromol..

[288] Shah D.A., Kwon S.-J., Bale S.S., Banerjee A., Dordick J.S., Kane R.S. (2011). Regulation of Stem Cell Signaling by Nanoparticle-Mediated Intracellular Protein Delivery. Biomaterials.

[289] Hong I.-S., Jang G.-B., Lee H.-Y., Nam J.-S., An S.S. (2015). Targeting Cancer Stem Cells by Using the Nanoparticles. Int. J. Nanomed..

[290] Yi Y., Tang H., Pi P., Zhang H., Du S., Ge W., Dai Q., Zhao Z., Li J., Sun Z. (2024). Melatonin in Cancer Biology: Pathways, Derivatives, and the Promise of Targeted Delivery. Drug Metab. Rev..

[291] Patel S.S., Acharya A., Ray R.S., Agrawal R., Raghuwanshi R., Jain P. (2020). Cellular and Molecular Mechanisms of Curcumin in Prevention and Treatment of Disease. Crit. Rev. Food Sci. Nutr..

[292] Shelash Al-Hawary S.I., Abdalkareem Jasim S., Kadhim M.M., Jaafar Saadoon S., Ahmad I., Romero Parra R.M., Hasan Hammoodi S., Abulkassim R., Hameed N.M., Alkhafaje W.K. (2023). Curcumin in the Treatment of Liver Cancer: From Mechanisms of Action to Nanoformulations. Phytother. Res..

[293] Khan S.H., Alhumaydhi F.A., Khan M.A., Younus H. (2021). Therapeutic Potential of Polyphenols and Their Nanoformulations in the Treatment of Colorectal Cancer. Anti-Cancer Agents Med. Chem..

[294] Najafiyan B., Bokaii Hosseini Z., Esmaelian S., Firuzpour F., Rahimipour Anaraki S., Kalantari L., Hheidari A., Mesgari H., Nabi-Afjadi M. (2024). Unveiling the Potential Effects of Resveratrol in Lung Cancer Treatment: Mechanisms and Nanoparticle-Based Drug Delivery Strategies. Biomed. Pharmacother..

[295] Mohapatra P., Madhulika S., Behera S., Singh P., Sa P., Prasad P., Swain R.K., Sahoo S.K. (2023). Nimbolide-Based Nanomedicine Inhibits Breast Cancer Stem-like Cells by Epigenetic Reprogramming of DNMTs-SFRP1-Wnt/β-Catenin Signaling Axis. Mol. Ther.—Nucleic Acids.

[296] Liu Y., Guerrero D., Lechuga-Ballesteros D., Tan M., Ahmad F., Aleiwi B., Ellsworth E., Chen B., Chua M.-S., So S. (2024). Lipid-Based Self-Microemulsion of Niclosamide Achieved Enhanced Oral Delivery and Anti-Tumor Efficacy in Orthotopic Patient-Derived Xenograft of Hepatocellular Carcinoma in Mice. Int. J. Nanomed..

[297] Wang Z., Zhang M., Luo W., Zhang Y., Ji H. (2021). Discovery of 2-(3-(3-Carbamoylpiperidin-1-Yl)Phenoxy)Acetic Acid Derivatives as Novel Small-Molecule Inhibitors of the β-Catenin/B-Cell Lymphoma 9 Protein–Protein Interaction. J. Med. Chem..

[298] You W., Ma F., Zhang Z., Yan J. (2022). Turning a Targeting β-Catenin/Bcl9 Peptide Inhibitor into a GdOF@Au Core/Shell Nanoflower for Enhancing Immune Response to Cancer Therapy in Combination with Immune Checkpoint Inhibitors. Pharmaceutics.

[299] Yang G., Zhang J., You W., Zhao X., Hou P., He W., Yan J., Guo H. (2020). Targeted Disruption of the BCL9/β-Catenin Interaction by Endosomal-Escapable Nanoparticles Functionalized with an E-Cadherin-Derived Peptide. Nanotechnology.

[300] Sokolov D., Sharda N., Banerjee A., Denisenko K., Basalious E.B., Shukla H., Waddell J., Hamdy N.M., Banerjee A. (2024). Differential Signaling Pathways in Medulloblastoma: Nano-Biomedicine TargetingNon-Coding Epigenetics to Improve Current and Future Therapeutics. Curr. Pharm. Des..

[301] Yousefnia S., Seyed Forootan F., Seyed Forootan S., Nasr Esfahani M.H., Gure A.O., Ghaedi K. (2020). Mechanistic Pathways of Malignancy in Breast Cancer Stem Cells. Front. Oncol..

[302] Li Q.-Y., Gong T., Huang Y.-K., Kang L., Warner C.A., Xie H., Chen L.-M., Duan X.-Q. (2023). Role of Noncoding RNAs in Liver Fibrosis. World J. Gastroenterol..

[303] Chandramohan K., Balan D.J., Devi K.P., Nabavi S.F., Reshadat S., Khayatkashani M., Mahmoodifar S., Filosa R., Amirkhalili N., Pishvaei S. (2023). Short Interfering RNA in Colorectal Cancer: Is It Wise to Shoot the Messenger?. Eur. J. Pharmacol..

[304] Singh S., Saxena S., Sharma H., Paudel K.R., Chakraborty A., MacLoughlin R., Oliver B.G., Gupta G., Negi P., Singh S.K. (2024). Emerging Role of Tumor Suppressing microRNAs as Therapeutics in Managing Non-Small Cell Lung Cancer. Pathology—Res. Pract..

[305] Tolcher A.W., Papadopoulos K.P., Patnaik A., Rasco D.W., Martinez D., Wood D.L., Fielman B., Sharma M., Janisch L.A., Brown B.D. (2015). Safety and Activity of DCR-MYC, a First-in-Class Dicer-Substrate Small Interfering RNA (DsiRNA) Targeting MYC, in a Phase I Study in Patients with Advanced Solid Tumors. J. Clin. Oncol..

[306] Rodriguez-Rivera I.I., Wu T.-H., Ciotti R., Senapedis W., Sullivan K., Gao J.Z., Palakurthi S., McCauley T., Moore Y. (2023). A Phase 1/2 Open-Label Study to Evaluate the Safety, Tolerability, Pharmacokinetics, Pharmacodynamics, and Preliminary Antitumor Activity of OTX-2002 as a Single Agent and in Combination with Standard of Care in Patients with Hepatocellular Carcinoma and Other Solid Tumor Types Known for Association with the MYC Oncogene (MYCHELANGELO I). J. Clin. Oncol..

[307] CancerNetwork OTX-2002 Shows Encouraging Safety in Small Hepatocellular Carcinoma Cohort. https://www.cancernetwork.com/view/otx-2002-shows-encouraging-safety-in-small-hepatocellular-carcinoma-cohort.

[308] Zhang L.-M., Li M., Tian C.-C., Wang T.-T., Mi S.-F. (2021). CCAAT Enhancer Binding Protein α Suppresses Proliferation, Metastasis, and Epithelial-Mesenchymal Transition of Ovarian Cancer Cells via Suppressing the Wnt/β-Catenin Signaling. Neoplasma.

[309] Setten R.L., Lightfoot H.L., Habib N.A., Rossi J.J. (2018). Development of MTL-CEBPA: Small Activating RNA Drug for Hepatocellular Carcinoma. Curr. Pharm. Biotechnol..

[310] Sarker D., Plummer R., Meyer T., Sodergren M.H., Basu B., Chee C.E., Huang K.-W., Palmer D.H., Ma Y.T., Evans T.R.J. (2020). MTL-CEBPA, a Small Activating RNA Therapeutic Upregulating C/EBP-α, in Patients with Advanced Liver Cancer: A First-in-Human, Multicenter, Open-Label, Phase I Trial. Clin. Cancer Res..

[311] Fakhri S., Moradi S.Z., Faraji F., Farhadi T., Hesami O., Iranpanah A., Webber K., Bishayee A. (2023). Current Advances in Nanoformulations of Therapeutic Agents Targeting Tumor Microenvironment to Overcome Drug Resistance. Cancer Metastasis Rev..

[312] Ansari M.A., Thiruvengadam M., Venkidasamy B., Alomary M.N., Salawi A., Chung I.-M., Shariati M.A., Rebezov M. (2022). Exosome-Based Nanomedicine for Cancer Treatment by Targeting Inflammatory Pathways: Current Status and Future Perspectives. Semin. Cancer Biol..

[313] Sun L., Liu H., Ye Y., Lei Y., Islam R., Tan S., Tong R., Miao Y.-B., Cai L. (2023). Smart Nanoparticles for Cancer Therapy. Sig Transduct. Target. Ther..

[314] Gaspar C., Franken P., Molenaar L., Breukel C., Van Der Valk M., Smits R., Fodde R. (2009). A Targeted Constitutive Mutation in the Apc Tumor Suppressor Gene Underlies Mammary But Not Intestinal Tumorigenesis. PLoS Genet..

[315] Letai A., Bhola P., Welm A.L. (2022). Functional Precision Oncology: Testing Tumors with Drugs to Identify Vulnerabilities and Novel Combinations. Cancer Cell.

[316] Rodríguez N., Viñal D., Rodríguez-Cobos J., De Castro J., Domínguez G. (2020). Genomic Profiling in Oncology Clinical Practice. Clin. Transl. Oncol..

[317] Cook D.P., Vanderhyden B.C. (2019). Ovarian Cancer and the Evolution of Subtype Classifications Using Transcriptional Profiling†. Biol. Reprod..

[318] Mokhtari K., Peymani M., Rashidi M., Hushmandi K., Ghaedi K., Taheriazam A., Hashemi M. (2023). Colon Cancer Transcriptome. Prog. Biophys. Mol. Biol..

[319] Huang P., Gao W., Fu C., Tian R. (2023). Functional and Clinical Proteomic Exploration of Pancreatic Cancer. Mol. Cell. Proteom..

[320] Wong G.Y.M., Diakos C., Hugh T.J., Molloy M.P. (2022). Proteomic Profiling and Biomarker Discovery in Colorectal Liver Metastases. Int. J. Mol. Sci..

[321] Madama D., Martins R., Pires A.S., Botelho M.F., Alves M.G., Abrantes A.M., Cordeiro C.R. (2021). Metabolomic Profiling in Lung Cancer: A Systematic Review. Metabolites.

[322] Aurilio G., Santoni M., Massari F., Cimadamore A., Rizzo A., Mollica V., Verri E., Battelli N., Montironi R. (2021). Metabolomic Profiling in Renal Cell Carcinoma Patients: News and Views. Cancers.

[323] Wojas-Krawczyk K., Paśnik I., Kucharczyk T., Wieleba I., Krzyżanowska N., Gil M., Krawczyk P., Milanowski J. (2021). Immunoprofiling: An Encouraging Method for Predictive Factors Examination in Lung Cancer Patients Treated with Immunotherapy. Int. J. Mol. Sci..

[324] Koelzer V.H., Sirinukunwattana K., Rittscher J., Mertz K.D. (2019). Precision Immunoprofiling by Image Analysis and Artificial Intelligence. Virchows Arch..

[325] Chakravarty D., Solit D.B. (2021). Clinical Cancer Genomic Profiling. Nat. Rev. Genet..

[326] Borhani A.A., Catania R., Velichko Y.S., Hectors S., Taouli B., Lewis S. (2021). Radiomics of Hepatocellular Carcinoma: Promising Roles in Patient Selection, Prediction, and Assessment of Treatment Response. Abdom. Radiol..

[327] Babu M., Snyder M. (2023). Multi-Omics Profiling for Health. Mol. Cell. Proteom..

[328] Fountzilas E., Tsimberidou A.M., Vo H.H., Kurzrock R. (2022). Clinical Trial Design in the Era of Precision Medicine. Genome Med..

[329] Perez E.A., Ramirez A.G., Trapido E.J. (2020). Biomarkers and Precision Medicine in Oncology Practice and Clinical Trials. Advancing the Science of Cancer in Latinos.

[330] Mortezaee K. (2024). WNT/β-Catenin Regulatory Roles on PD-(L)1 and Immunotherapy Responses. Clin. Exp. Med..

