# Supplementary material for: Why Is Wnt/β-Catenin Not Yet Targeted in Routine Cancer Care?
Source: Pharmaceuticals (Basel). 2024 Jul 16;17(7):949. doi: 10.3390/ph17070949 (PMC11279613; doi:10.3390/ph17070949)
Supplement: Supplementary file 1 [file pharmaceuticals-17-00949-s001.zip › pharmaceuticals-3072914-supplementary.pdf]

Table S1. A few highlights from Wnt discovery to the design of Wnt/β-catenin inhibitors as anti-cancer agents

Years	Highlights
1936	Discovery of a milk-transmitted susceptibility to develop mammary gland tumors in mice (Bittner 1936 [1])
1962	Purification of the mouse mammary tumor virus (MMTV) responsible for mammary tumor development in mice (Lyons and Moore 1962 [2])
1982	Identification of the MMTV Int1 gene responsible for mammary tumor development in mice (Nusse et Varmus 1982 [3])
1984	Elucidation of the structure and sequence of the Int1 gene (Van Ooyen et Nusse 1984 [4])
1985	Int1 is conserved among species (Van Ooyen et al. 1985 [5])
	The Int1 gene cDNA sequence is elucidated and expressed *in vitro* (Fung et al. 1985 [6])
1988	Mice carrying an Int1 encoding transgene under the control of a MMTV transcriptional regulator spontaneously develop breast tumors (Tsukamoto et al. 1988 [7])
1989	The Frizzled gene encodes a seven-pass transmembrane protein (Vinson et al. 1989 [8])
1990	Disrupting Int1 in mice results in severe abnormalities in midbrain and cerebellar development (Thomas and Capecchi 1990 [9])
	Multiple novel Wnt-1/int-1-related genes are expressed during fetal and adult mouse development (Gavin et al. 1990 [10])
**1991**	**Start in of the first Wnt meeting**
	Cloning of the human APC gene which is mutated in Familial Adenomatous Polyposis (FAP) and colon cancers (Groden et al. 1991; Kinzler et al. 1991 [11,12])
1992	Int1 is renamed as Wnt-1, the founding member of the Wnt family (Nusse and Varmus 1992 [13])
	GSK3β is a negative regulator of the Wnt pathway (Siegfried et al. 1992 [14])
	Somatic and germlines mutations are clustered within the APC gene in inherited and sporadic colorectal cancer patients (Miyoshi et al. 1992 [15,16])
1993	ApcMin, a mutation in the murine Apc gene, predisposes to mammary carcinomas and focal alveolar hyperplasias (Moser et al. 1993 [17])
1994	A targeted chain-termination mutation in the mouse Apc gene results in multiple intestinal tumors (Fodde et al. 1994 [18])
1995	β-catenin (Armadillo in Drosophila) is present in the cell cytoplasm, plasma membrane and nucleus (Funayama et al. 1995 [19])
	APC associates with β-catenin and regulates its intracellular levels (Munemitsu et al. 1995 [20])
1996	The axis-inducing activity, stability, and subcellular distribution of β-catenin is regulated in Xenopus embryos by Glycogen Synthase Kinase 3 (Yost et al., 1996 [21])
	The HMG-box containing transcription factor TCF/ Lef1 interacts with β-catenin (Molenaar et al. 1996; Behrens et al. 1996; Huber et al. 1996 [22-24])
	Porcupin encodes an acyltransferase involved in Wnt post-translational modification required for Wnt secretion (Kadowaki et al. 1996 [25])
**1997**	**Design of the Website "Wnt homepage"** *(http://web.stanford.edu/group/nusselab/cgi-bin/wnt/)*
	The protein Frzb-1 which shares homology with Dishevelled secreted molecules is purified and cloned (Finch et al. 1997; Leyns et al. 1997; Rattner et al. 1997 [26-28])
	Phosphorylated β-catenin is targeted for degradation by the ubiquitination/proteasome pathway (Aberle et al. 1997; Orford et al. 1997 [29,30])
	Wingless signaling is mediated by the transcription factor TCF/Lef1 (Pangolin in *Drosophila*) (Brunner et al. 1997, Clevers et Van De Wetering 1997 [31,32])
	APC is mutated in about 85% of sporadic colon cancers, 5–10% of sporadic colon cancers and mutations alter the phosphorylation sites that target β-catenin for degradation (Korinek et al. 1997; Morin et al. 1997; Rubinfeld et al. 1997 [33-35])
1998	Axin is a component of the β-catenin degradation complex, together with APC and GSK3β (Behrens et al. 1998; Hart et al. 1998; Kishida et al. 1998; Nakamura et al. 1998 [36-39])
	TCF/Lef1 represses the activity of Wingless signaling when bound to Groucho (Cavallo et al. 1998 [40])
	Dickkopf-1 is a member of a new family of secreted proteins capable of inhibiting Wnt signaling (Glinka et al. 1998 [41])
	Frizzled genes are often redundant and display phenotypes only when mutated in combination with other family members (Kennerdell et Carthew. 1998 [42])
	The Myc oncogene is a target of APC/Wnt signalling (He et al. 1998 [43])
	Somatic mutations of β-catenin are frequent in mouse and human hepatocellular carcinomas (de La Coste et al. 1998; Miyoshi et al. 1998 [44,45])
1999	WIF-1 is a new secreted protein which binds to Wnt proteins and inhibit their activity (Hsieh et al. 1999 [46])
	A TOPGal Wnt reporter is generated to study how cell fate and differenciation take place during hair follicule development (DasGupta et Fuchs. 1999 [47])
	Wingless signaling is negatively regulated by d-Axin, a Drosophila homolog of Axin (Hamada et al. 1999 [48])
	Apc1638T is a mouse model delineating APC domains critical for tumorigenesis and development (Smits et al. 1999 [49])
	TCF4 mutations are found in gastrointestinal cancers (Duval et al. 1999 [50,51])
2000	Arrow encodes an LDL-receptor-related protein (LRP) essential for Wingless signalling which is a co-receptor for Wnts, closely located to Frizzleds in the cell membrane (Wehrli et al. 2000; Pinson et al. 2000; Tamai et al. 2000 [52-54])
	Frizzled proteins have distinct affinities for different Wnts (Rulifson et al. 2000 [55])
	The Derailed/RYK extracellular domain shares similarities with the WIF protein (Patthy. 2000 [56])
	Characterization of the structural basis of the Axin–APC interaction (Spink et al. 2000 [57])
	The p300/CBP acetyltransferases function as transcriptional co-activators of β-catenin in vertebrates (Hecht et al. 2000 [58])
	Axin mutations are found in colorectal and other cancers (Liu et al. 2000; Satoh et al. 2000 [59,60])
2001	Arrow/LRP is the target of several Wnt antagonists including Dickkopf (Bafico et al. 2001; Mao et al. 2001; Semenov et al. 2001 [61-63])
	The nonsteroidal anti-inflammatory compound **Sulindac** inhibits the Wnt/β-catenin signaling (Tutter ert al. 2001 [64])
2002	Axin mediates β-catenin phosphorylation by CK1 and inhibits the Wnt/β-catenin signaling (Amit et al. 2002; Liu et al. 2002 [65,66])
	Wnt/β-catenin/Tcf signaling induces the transcription of Axin2, a negative regulator of the signaling pathway (Jho et al. 2002 [67])
	The Axin- like protein PRY-1 is a negative regulator of a canonical Wnt pathway in C. elegans (Korswagen et al. 2002 [68])
2003	Identification of RORs and Derailed/RYK as new Wnt receptors (Oishi et al, 2003; Yoda et al. 2003; Yoshikawa et al. 2003 [69-70])
	Dishevelled relocates Axin to the plasma membrane during Wingless signalling (Cliffe et al. 2003 [71])
	Axin is the rate-limiting of the β-catenin degradation complex (Lee et al, 2003 [72])
2004	Characterization of the phosphorylation-dependent binding of APC to β-catenin and its role in β-catenin degradation (Ha et al. 2004 [73])
	The essential requirement for Wnt signaling in proliferation of adult small intestine and colon is revealed by adenoviral expression of Dickkopf-1 (Kuhnert et al. 2004 [74])
	APC dosage effects explain how a single pathway is involved in different tissue development, homeostasis, and in tumorigenesis (Gaspar et Fodde. 2004 [75])
	Mutations in AXIN2 cause familial tooth agenesis and predispose to colorectal cancer (Lammi et al. 2004 [76])
	Arrow/LRP cytoplasmic tail is phosphorylated as a consequence of Wnt binding and interacts directly with GSK3 and Axin (Tamai et al. 2004 [77])

2005	Identification of a selective inhibitor (**ICG001**) of a subset of Wnt/β-catenin-driven gene expression (McMillan et Kahn 2005 [78])
	Characterization of a dual kinase-kinase mechanism for Wnt-co-receptor phosphorylation and activation (Zeng et al. 2005 [79])
	Mutations of β-catenin and AXIN I genes are a late event in human hepatocellular carcinogenesis (Park et al. 2005 [80])
2006	The Dickkopf-Wnt antagonism is conserved across many animal species including hydra (Guder et al. 2006 [81])
	Identification of Wntless/Evi, which shuttles between the ER and the plasma membrane for Wnt secretion (Banziger et al. 2006; Ching et Nusse 2006; Bartscherer et al. 2006 [82-84])
	Stem cells are Wnt dependent (Shackleton et al. 2006 [85])
2007	Axin is recruted at the plasma membrane via dynamic Dishevelled-dependent assemblies (Schwarz-Romond et al. 2007 [86])
	Identification of Myc as the master gene responsible for the oncogenicity of the Wnt/β-catenin signalling pathway (Sansom et al. 2007 [87])
2008	APC is essential for targeting phosphorylated β-catenin to the SCFβ-TrCP ubiquitin ligase (Su et al. 2008 [88])
	The Wnt-5a-derived hexapeptide **Foxy-5** inhibits breast cancer metastasis *in vivo* by targeting cell motility (Säfholm et al. 2008 [89])
2009	Frizzled's intracellular role in signalling is limited to binding Dishevelled (Macdonald et al. 2009 [90])
	The Apc1322T mouse develops severe polyposis associated with submaximal nuclear β-catenin expression (Pollard et al. 2009 [91])
	APC truncations underlies mammary but not intestinal tumorigenesis in mice (Gaspar et al. 2009 [92])
	Tankyrase inhibition by the small molecule **XAV939** stabilizes axin and antagonizes Wnt signaling (Huang et al. 2009 [93])
	The anti-DKK1 monoclonal antibody **BHQ880** is a potential therapeutic agent for multiple myeloma (Heath et al. 2009; Fulciniti et al. 2009; [94,95])
2010	Wnt proteins or Wnt agonists can be used to expand stem cells in culture (Zeng et Nusse, 2010 [96])
	Severe polyposis in Apc(1322T) mice is associated with submaximal Wnt signalling and increased expression of the stem cell marker Lgr5 (Lewis et al. 2010 [97)
	Development of a high-throughput screening of small molecules targeting the Wnt pathway : **Niclosamide** is identified as a Wnt/β-catenin inhibitor (Chen et al. 2010 [98])
	**Pyrvinium** is a potent small molecule Wnt inhibitor (Saraswati et al; 2010; Thorne et al. 2010 [99,100])
	Anti-tumor activity of a novel small molecule inhibitor of Wnt signaling (**CWP232291**) in multiple myeloma (Cha et al. 2010 [101])
2011	Structural and functional studies of LRP6 ectodomain reveal a platform for Wnt signaling (Chen et al. 2011 [102])
	Paneth cells constitute the niche for Lgr5 stem cells in intestinal crypts (Sato et al, 2011 [103])
	A new model for the β-catenin destruction complex in regulating Wnt signaling (Roberts et al. 2011 [104])
	Functional proteins can be delivered intracellularly in vitro using **nanoparticles** in order to target the Wnt/β-catenin signaling pathway (Shah et al. 2011 [105])
2012	Characterization of the structural basis of Wnt recognition by frizzled (Janda et al. 2012 [106])
	Wnt/β-catenin signaling requires interaction of the Dishevelled DEP domain and C terminus with a discontinuous motif in Frizzled (Tauriello et al. 2012 [107])
	Tumour suppressor RNF43 is a stem-cell E3 ligase that induces endocytosis of Wnt receptors (Koo et al. 2012 [108])
	R-spondin gene fusions are recurrent in colon cancer (Seshagiri et al. 2012 [109])
	Conditional disruption of Axin1 leads to development of liver tumors in mice (Feng et al. 2012 [110])
2013	Characterization of the structural basis of R-spondin recognition by LGR5 and RNF43 (Chen et al. 2013 [111])
	Abnormal hypermethylation and clinicopathological significance of Axin gene in lung cancer (Yang et al. 2013 [112])
	Wnt secretion is required to maintain high levels of Wnt activity in colon cancer cells (Voloshanenko et al. 2013 [113])
	A basal gradient of Wnt and stem-cell number influences regional tumour distribution in human and mouse intestinal tracts (Leedham et al. 2013 [114])
	Inactivating mutations of RNF43 confer Wnt dependency in pancreatic ductal adenocarcinoma (Jiang et al. 2013 [115])
	Wnt-driven cancers can be targeted through inhibition of Porcupine by **LGK974** (Liu et al. 2013 [116])
	First-in-human evaluation of the human monoclonal antibody **Vantictumab** (**OMP-18R5**) in patients with advanced solid tumors (Smith et al. 2013 [117])
2014	The ubiquitin ligase RNF220 enhances canonical Wnt signaling through USP7-mediated de-ubiquitination of β-catenin (Ma et al. 2014 [118])
	RNF43 is frequently mutated in colorectal and endometrial cancers (Giannakis et al. 2014 [119])
	Overexpression of RNF146 in non-small cell lung cancer enhances proliferation and invasion of tumors through the wnt/β-catenin signaling pathway (Gao et al. 2014 [120])
	AXIN2 germline variant are associated with attenuated FAP (Rivera et al. 2014 [121])
2015	Dishevelled promotes Wnt receptor degradation through recruitment of ZNRF3/RNF43 E3 ubiquitin ligases (Jiang et al. 2015 [122])
	Apc Restoration Promotes Cellular Differentiation and Re-establishes Crypt Homeostasis in Colorectal Cancer (Dow et al. 2015 [123)
	**E7449** is a dual inhibitor of PARP1/2 and tankyrase1/2 which inhibits growth of DNA repair deficient tumors and antagonizes Wnt signaling (Mc Gonigle et al. 2015 [124])
	Porcupine inhibitor **C59** suppresses paracrine Wnt-driven growth of ZNRF3/RNF43-mutant neoplasia (Koo et al. 2015 [125])
2016	Tankyrase requires SAM domain-dependent polymerization to support Wnt–β-catenin signaling (Mariotti et al. 2016 [126])
	The poly(ADP-ribose) polymerase enzyme tankyrase antagonizes activity of the β-catenin destruction complex through ADP-ribosylation of Axin and APC2 (Croy et al. 2016 [127])
	Frequent PTPRK–RSPO3 fusions and RNF43 mutations are found in colorectal traditional serrated adenoma (Sekine et al. 2016 [128])
	AXIN2 polymorphisms are associated with colorectal cancer in Mexican patients (Rosales- Reynoso et al. 2016 [129])
	Wnt addiction of genetically defined cancers can be reversed by the PORCN inhibitor **ETC-1922159** (Madan et al. 2016 [130])
2017	R-Spondin chromosome rearrangements drive Wnt- dependent tumour initiation and maintenance in the intestine (Han et al. 2017 [131])
	Functional redundancy between Apc and Apc2 regulates tissue homeostasis and prevents tumorigenesis in murine mammary epithelium (Daly et al. 2017 [132])
	AXIN1 loss is a mutational route for resistance to treatment with PORCN inhibitors (such as **LGK974**) (Picco et al. 2017 [133])
	Genetic variations in Axin2 gene are associated with lung cancer risk in North Indian population (Bahl et al. 2017 [134])
	A first-in-human phase I study of the anti-cancer stem cell agent **Ipafricept** (**OMP-54F28**), a decoy receptor for Wnts, in Patients with Advanced Solid Tumors (Jimeno et al. 2017 [135])
	APC mutations may be used as potential biomarker for sensitivity to tankyrase inhibitors in colorectal cancer (Tanaka et al. 2017 [136])
	The novel porcupine (PORCN) inhibitor **RXC004** is evaluated in xenograft and PDX mouse models of RNF43 loss of function cancers (Bhamra et al. 2017 [137])
2018	R-spondins can potentiate WNT signaling through distinct mechanisms (Lebensohn et al. 2018; Park et al. 2018 [138,139])
	Supramolecular assembly of the β-catenin destruction complex and the effect of Wnt signaling on its localization, molecular size, and activity in vivo (Schaefer et al. 2018 [140])
	Hypermethylation of APC2 is a predictive epigenetic biomarker for Chinese colorectal cancer (He et al. 2018 [141])
	The E3 ubiquitin ligase RNF146 promotes colorectal cancer by activating the Wnt/β-catenin pathway via ubiquitination of Axin1 (Shen et al. 2018 [142])
	Wnt signalling activation by RNF43 mutations enhances tumour growth and promotes a high recurrence rate in colorectal cancer patients (Eto et al. 2018 [143])
	Germline RNF43 mutations are a rare cause of serrated polyposis (Quintana et al. 2018 [144])
	A first-in-human study with a new radiolabeled MAb targeting FZD10 (**OTSA-101**) in metastatic synovial sarcoma patients (Giraudet et al. 2018 [145])
	Characterization of a human specific monoclonal antibody (**BNC101**) targeting the GPCR LGR5 (Inglis et al. 2018 [146])
	Preclinical study of **Tegavivint**, a novel Wnt/β-catenin pathway inhibitor (Nomura et al. 2018 [147])

2019	A ZNRF3-dependent Wnt/ β-catenin signaling gradient is required for adrenal homeostasis (Basham et al. 2019 [148])
	USP7 inhibits Wnt/β-catenin signaling through promoting stabilization of Axin (Ji et al. 2019 [149])
	Loss of endogenous RNF43 function enhances proliferation and tumour growth of intestinal and gastric cells (Neumeyer et al. 2019 [150])
	Variations in AXIN2 predict risk and prognosis of colorectal cancer (Otero et al. 2019 [151])
	**Chloroquine** reverts Paclitaxel resistance and attenuates metastatic potential in human nonsmall lung adenocarcinoma (Datta et al. 2019 [152])
2020	R-Spondins engage heparan sulfate proteoglycans to potentiate Wnt signaling (Dubey et al. 2020 [153])
	RNF43 truncations trap CK1 to drive niche-independent self-renewal in cancer (Spit et al. 2020 [154])
	Frequent RNF43 mutation contributes to moderate activation of Wnt signaling in colorectal signet-ring cell carcinoma (Li et al. 2020 [155])
	Biology drives the discovery of **bispecific antibodies** as innovative therapeutics (Nie et al. 2020 [156])
	**DKN-01** is a promising immunomodulatory combination partner for cancer treatment (Wall et al. 2020 [157])
	Targeting the Wnt signaling pathway through the anti-R-spondin 3 antibody **Rosmantuzumab** (**OMP-131R10**) identifies an anti-fibrosis treatment strategy (Zhang et al. 2020 [158])
	The CLK inhibitor **SM08502** (**Cirtuvivint**) induces anti-tumor activity and reduces Wnt pathway gene expression in gastrointestinal cancer models (Tam et al. 2020 [159])
2021	A phase I dose-escalation and expansion study evaluates the a dual inhibitor of PARP/tankyrase **JPI-547** (**Nesuparib**) in patients with advanced solid tumors (Im et al. 2021 [160])
	**E7386**, a selective inhibitor of the interaction between β-catenin and CBP, exerts antitumor activity in tumor models with activated canonical Wnt signaling (Yamada et al. 2021 [161])
2022	A phase I, open-label, dose-escalation study investigates a LRP 5/6 inhibitor (**BI 905677**) in patients with advanced solid tumors (Elez et al. 2022 [162])
2023	Structure of the Wnt-Frizzled-LRP6 initiation complex reveals the basis for co-receptor discrimination (Tsutsumi et al. 2023 [163])
	Wnt/β-catenin signaling is required for pole-specific chromatin remodeling during planarian regeneration (Pascual-Carreras et al. 2023 [164])
	**WHN-88** is a novel PORCN inhibitor that prevents the growth of Wnt-driven cancers (Yang et al. 2023 [165])
2024	MiR-5590-3p inhibits the proliferation and invasion of ovarian cancer cells through mediating the Wnt/beta-catenin signaling pathway by targeting TNIK (Wu et al. 2024 [166])
	Synergistic cytotoxicity and modulation of apoptosis and Wnt/β-catenin signaling pathway by Doxorubicin-loaded zymosan **nanoparticles** in colorectal cancer cells (Rajabi et al. 2024 [167])
	Targeting Wnt signaling with the Wnt/CBP/β-catenin inhibitor **ICG-001** improves glioma immunotherapy (Gutova et al. 2024 [168])
	WNT/β-catenin signaling palys regulatory roles on PD-(L)1 and immunotherapy responses (Mortezaee. 2024 [169])

Findings in development and tissue homeostasis
Findings in cancers
Antibodies used as Wnt/β-catenin dependent inhibitors (WDi)
Small molecules used as Wnt/β-catenin dependent inhibitors (WDi)
Small molecules used as Wnt/β-catenin independent inhibitors (WIi) preventing β-catenin stabilization
Small molecules used as Wnt/β-catenin independent inhibitors (WIi) preventing β-catenin co-transcriptional activity

1. Bittner, J.J. Some Possible Effects of Nursing on the Mammary Gland Tumor Incidence in Mice. *Science* **1936**, *84*, 162–162, doi:10.1126/science.84.2172.162.a.
2. Lyons, M.J.; Moore, D.H. Purification of the Mouse Mammary Tumour Virus. *Nature* **1962**, *194*, 1141–1142, doi:10.1038/1941141a0.
3. Nusse, R.; Varmus, H.E. Many Tumors Induced by the Mouse Mammary Tumor Virus Contain a Provirus Integrated in the Same Region of the Host Genome. *Cell* **1982**, *31*, 99–109, doi:10.1016/0092-8674(82)90409-3.
4. Van Ooyen, A.; Nusse, R. Structure and Nucleotide Sequence of the Putative Mammary Oncogene Int-1; Proviral Insertions Leave the Protein-Encoding Domain Intact. *Cell* **1984**, *39*, 233–240, doi:10.1016/0092-8674(84)90209-5.
5. van Ooyen, A.; Kwee, V.; Nusse, R. The Nucleotide Sequence of the Human Int-1 Mammary Oncogene; Evolutionary Conservation of Coding and Non-Coding Sequences. *EMBO J* **1985**, *4*, 2905–2909, doi:10.1002/j.1460-2075.1985.tb04021.x.
6. Fung, Y.-K.T.; Shackleford, G.M.; Brown, A.M.C.; Sanders, G.S.; Varmus, H.E. Nucleotide Sequence and Expression In Vitro of cDNA Derived from mRNA of *Int* -1, a Provirally Activated Mouse Mammary Oncogene. *Molecular and Cellular Biology* **1985**, *5*, 3337–3344, doi:10.1128/mcb.5.12.3337-3344.1985.
7. Tsukamoto, A.S.; Grosschedl, R.; Guzman, R.C.; Parslow, T.; Varmus, H.E. Expression of the Int-1 Gene in Transgenic Mice Is Associated with Mammary Gland Hyperplasia and Adenocarcinomas in Male and Female Mice. *Cell* **1988**, *55*, 619–625, doi:10.1016/0092-8674(88)90220-6.
8. Vinson, C.R.; Conover, S.; Adler, P.N. A Drosophila Tissue Polarity Locus Encodes a Protein Containing Seven Potential Transmembrane Domains. *Nature* **1989**, *338*, 263–264, doi:10.1038/338263a0.
9. Thomas, K.R.; Capecchi, M.R. Targeted Disruption of the Murine Int-1 Proto-Oncogene Resulting in Severe Abnormalities in Midbrain and Cerebellar Development. *Nature* **1990**, *346*, 847–850, doi:10.1038/346847a0.

10. Gavin, B.J.; McMahon, J.A.; McMahon, A.P. Expression of Multiple Novel Wnt-1/Int-1-Related Genes during Fetal and Adult Mouse Development. *Genes & Development* **1990**, *4*, 2319–2332, doi:10.1101/gad.4.12b.2319.

11. Groden, J.; Thliveris, A.; Samowitz, W.; Carlson, M.; Gelbert, L.; Albertsen, H.; Joslyn, G.; Stevens, J.; Spirio, L.; Robertson, M.; et al. Identification and Characterization of the Familial Adenomatous Polyposis Coli Gene. *Cell* **1991**, *66*, 589–600, doi:10.1016/0092-8674(81)90021-0.

12. Kinzler, K.W.; Nilbert, M.C.; Su, L.-K.; Vogelstein, B.; Bryan, T.M.; Levy, D.B.; Smith, K.J.; Preisinger, A.C.; Hedge, P.; McKechnie, D.; et al. Identification of FAP Locus Genes from Chromosome 5q21. *Science* **1991**, *253*, 661–665, doi:10.1126/science.1651562.

13. Nusse, R.; Varmus, H.E. Wnt Genes. *Cell* **1992**, *69*, 1073–1087, doi:10.1016/0092-8674(92)90630-U.

14. Siegfried, E.; Chou, T.-B.; Perrimon, N. Wingless Signaling Acts through Zeste-White 3, the Drosophila Homolog of Glycogen Synthase Kinase-3, to Regulate Engrailed and Establish Cell Fate. *Cell* **1992**, *71*, 1167–1179, doi:10.1016/S0092-8674(05)80065-0.

15. Miyoshi, Y.; Ando, H.; Nagase, H.; Nishisho, I.; Horii, A.; Miki, Y.; Mori, T.; Utsunomiya, J.; Baba, S.; Petersen, G. Germ-Line Mutations of the APC Gene in 53 Familial Adenomatous Polyposis Patients. *Proc. Natl. Acad. Sci. U.S.A.* **1992**, *89*, 4452–4456, doi:10.1073/pnas.89.10.4452.

16. Miyoshi, Y.; Nagase, H.; Ando, H.; Horii, A.; Ichii, S.; Nakatsuru, S.; Aoki, T.; Miki, Y.; Mori, T.; Nakamura, Y. Somatic Mutations of the APC Gene in Colorectal Tumors: Mutation Cluster Region in the APC Gene. *Hum Mol Genet* **1992**, *1*, 229–233, doi:10.1093/hmg/1.4.229.

17. Moser, A.R.; Mattes, E.M.; Dove, W.F.; Lindstrom, M.J.; Haag, J.D.; Gould, M.N. ApcMin, a Mutation in the Murine Apc Gene, Predisposes to Mammary Carcinomas and Focal Alveolar Hyperplasias. *Proc Natl Acad Sci U S A* **1993**, *90*, 8977–8981, doi:10.1073/pnas.90.19.8977.

18. Fodde, R.; Edelmann, W.; Yang, K.; van Leeuwen, C.; Carlson, C.; Renault, B.; Breukel, C.; Alt, E.; Lipkin, M.; Khan, P.M. A Targeted Chain-Termination Mutation in the Mouse Apc Gene Results in Multiple Intestinal Tumors. *Proc Natl Acad Sci U S A* **1994**, *91*, 8969–8973, doi:10.1073/pnas.91.19.8969.

19. Funayama, N.; Fagotto, F.; McCrea, P.; Gumbiner, B.M. Embryonic Axis Induction by the Armadillo Repeat Domain of Beta-Catenin: Evidence for Intracellular Signaling. *The Journal of cell biology* **1995**, *128*, 959–968, doi:10.1083/jcb.128.5.959.

20. Munemitsu, S.; Albert, I.; Souza, B.; Rubinfeld, B.; Polakis, P. Regulation of Intracellular Beta-Catenin Levels by the Adenomatous Polyposis Coli (APC) Tumor-Suppressor Protein. *Proc. Natl. Acad. Sci. U.S.A.* **1995**, *92*, 3046–3050, doi:10.1073/pnas.92.7.3046.

21. Yost, C.; Torres, M.; Miller, J.R.; Huang, E.; Kimelman, D.; Moon, R.T. The Axis-Inducing Activity, Stability, and Subcellular Distribution of Beta-Catenin Is Regulated in Xenopus Embryos by Glycogen Synthase Kinase 3. *Genes Dev.* **1996**, *10*, 1443–1454, doi:10.1101/gad.10.12.1443.

22. Molenaar, M.; Van De Wetering, M.; Oosterwegel, M.; Peterson-Maduro, J.; Godsave, S.; Korinek, V.; Roose, J.; Destrée, O.; Clevers, H. XTcf-3 Transcription Factor Mediates β-Catenin-Induced Axis Formation in Xenopus Embryos. *Cell* **1996**, *86*, 391–399, doi:10.1016/S0092-8674(00)80112-9.

23. Behrens, J.; Von Kries, J.P.; Kühl, M.; Bruhn, L.; Wedlich, D.; Grosschedl, R.; Birchmeier, W. Functional Interaction of β-Catenin with the Transcription Factor LEF-1. *Nature* **1996**, *382*, 638–642, doi:10.1038/382638a0.

24. Huber, O.; Korn, R.; McLaughlin, J.; Ohsugi, M.; Herrmann, B.G.; Kemler, R. Nuclear Localization of β-Catenin by Interaction with Transcription Factor LEF-1. *Mechanisms of Development* **1996**, *59*, 3–10, doi:10.1016/0925-4773(96)00597-7.

25. Kadowaki, T.; Wilder, E.; Klingensmith, J.; Zachary, K.; Perrimon, N. The Segment Polarity Gene Porcupine Encodes a Putative Multitransmembrane Protein Involved in Wingless Processing. *Genes Dev.* **1996**, *10*, 3116–3128, doi:10.1101/gad.10.24.3116.

26. Finch, P.W.; He, X.; Kelley, M.J.; Uren, A.; Schaudies, R.P.; Popescu, N.C.; Rudikoff, S.; Aaronson, S.A.; Varmus, H.E.; Rubin, J.S. Purification and Molecular Cloning of a Secreted, Frizzled-Related Antagonist of Wnt Action. *Proc*

Natl Acad Sci U S A **1997**, *94*, 6770–6775, doi:10.1073/pnas.94.13.6770.

27. Leyns, L.; Bouwmeester, T.; Kim, S.H.; Piccolo, S.; De Robertis, E.M. Frzb-1 Is a Secreted Antagonist of Wnt Signaling Expressed in the Spemann Organizer. *Cell* **1997**, *88*, 747–756, doi:10.1016/s0092-8674(00)81921-2.

28. Rattner, A.; Hsieh, J.C.; Smallwood, P.M.; Gilbert, D.J.; Copeland, N.G.; Jenkins, N.A.; Nathans, J. A Family of Secreted Proteins Contains Homology to the Cysteine-Rich Ligand-Binding Domain of Frizzled Receptors. *Proc Natl Acad Sci U S A* **1997**, *94*, 2859–2863, doi:10.1073/pnas.94.7.2859.

29. Aberle, H.; Bauer, A.; Stappert, J.; Kispert, A.; Kemler, R. β-Catenin Is a Target for the Ubiquitin–Proteasome Pathway. *EMBO J* **1997**, *16*, 3797–3804, doi:10.1093/emboj/16.13.3797.

30. Orford, K.; Crockett, C.; Jensen, J.P.; Weissman, A.M.; Byers, S.W. Serine Phosphorylation-Regulated Ubiquitination and Degradation of β-Catenin. *Journal of Biological Chemistry* **1997**, *272*, 24735–24738, doi:10.1074/jbc.272.40.24735.

31. Brunner, E.; Peter, O.; Schweizer, L.; Basler, K. Pangolinencodes a Lef-1 Homologue That Acts Downstream of Armadillo to Transduce the Wingless Signal in Drosophila. *Nature* **1997**, *385*, 829–833, doi:10.1038/385829a0.

32. Clevers, H.; Van De Wetering, M. TCF/LEF Factors Earn Their Wings. *Trends in Genetics* **1997**, *13*, 485–489, doi:10.1016/S0168-9525(97)01305-X.

33. Korinek, V.; Barker, N.; Morin, P.J.; van Wichen, D.; de Weger, R.; Kinzler, K.W.; Vogelstein, B.; Clevers, H. Constitutive Transcriptional Activation by a Beta-Catenin-Tcf Complex in APC-/- Colon Carcinoma. *Science* **1997**, *275*, 1784–1787, doi:10.1126/science.275.5307.1784.

34. Morin, P.J.; Sparks, A.B.; Korinek, V.; Barker, N.; Clevers, H.; Vogelstein, B.; Kinzler, K.W. Activation of Beta-Catenin-Tcf Signaling in Colon Cancer by Mutations in Beta-Catenin or APC. *Science* **1997**, *275*, 1787–1790, doi:10.1126/science.275.5307.1787.

35. Rubinfeld, B.; Albert, I.; Porfiri, E.; Munemitsu, S.; Polakis, P. Loss of Beta-Catenin Regulation by the APC Tumor Suppressor Protein Correlates with Loss of Structure Due to Common Somatic Mutations of the Gene. *Cancer Res* **1997**, *57*, 4624–4630.

36. Behrens, J.; Jerchow, B.-A.; Würtele, M.; Grimm, J.; Asbrand, C.; Wirtz, R.; Kühl, M.; Wedlich, D.; Birchmeier, W. Functional Interaction of an Axin Homolog, Conductin, with β-Catenin, APC, and GSK3β. *Science* **1998**, *280*, 596–599, doi:10.1126/science.280.5363.596.

37. Hart, M.J.; De Los Santos, R.; Albert, I.N.; Rubinfeld, B.; Polakis, P. Downregulation of β-Catenin by Human Axin and Its Association with the APC Tumor Suppressor, β-Catenin and GSK3β. *Current Biology* **1998**, *8*, 573–581, doi:10.1016/S0960-9822(98)70226-X.

38. Kishida, S.; Yamamoto, H.; Ikeda, S.; Kishida, M.; Sakamoto, I.; Koyama, S.; Kikuchi, A. Axin, a Negative Regulator of the Wnt Signaling Pathway, Directly Interacts with Adenomatous Polyposis Coli and Regulates the Stabilization of β-Catenin. *Journal of Biological Chemistry* **1998**, *273*, 10823–10826, doi:10.1074/jbc.273.18.10823.

39. Nakamura, T.; Hamada, F.; Ishidate, T.; Anai, K.; Kawahara, K.; Toyoshima, K.; Akiyama, T. Axin, an Inhibitor of the Wnt Signalling Pathway, Interacts with Β-catenin, GSK-3β and APC and Reduces the Β-catenin Level. *Genes to Cells* **1998**, *3*, 395–403, doi:10.1046/j.1365-2443.1998.00198.x.

40. Cavallo, R.A.; Cox, R.T.; Moline, M.M.; Roose, J.; Polevoy, G.A.; Clevers, H.; Peifer, M.; Bejsovec, A. Drosophila Tcf and Groucho Interact to Repress Wingless Signalling Activity. *Nature* **1998**, *395*, 604–608, doi:10.1038/26982.

41. Glinka, A.; Wu, W.; Delius, H.; Monaghan, A.P.; Blumenstock, C.; Niehrs, C. Dickkopf-1 Is a Member of a New Family of Secreted Proteins and Functions in Head Induction. *Nature* **1998**, *391*, 357–362, doi:10.1038/34848.

42. Kennerdell, J.R.; Carthew, R.W. Use of dsRNA-Mediated Genetic Interference to Demonstrate That Frizzled and Frizzled 2 Act in the Wingless Pathway. *Cell* **1998**, *95*, 1017–1026, doi:10.1016/S0092-8674(00)81725-0.

43. He, T.C.; Sparks, A.B.; Rago, C.; Hermeking, H.; Zawel, L.; da Costa, L.T.; Morin, P.J.; Vogelstein, B.; Kinzler, K.W. Identification of C-MYC as a Target of the APC Pathway. *Science* **1998**, *281*, 1509–1512, doi:10.1126/science.281.5382.1509.

44. de La Coste, A.; Romagnolo, B.; Billuart, P.; Renard, C.A.; Buendia, M.A.; Soubrane, O.; Fabre, M.; Chelly, J.; Beldjord, C.; Kahn, A.; et al. Somatic Mutations of the Beta-Catenin Gene Are Frequent in Mouse and Human Hepatocellular Carcinomas. *Proc Natl Acad Sci U S A* **1998**, *95*, 8847–8851, doi:10.1073/pnas.95.15.8847.

45. Miyoshi, Y.; Iwao, K.; Nagasawa, Y.; Aihara, T.; Sasaki, Y.; Imaoka, S.; Murata, M.; Shimano, T.; Nakamura, Y. Activation of the Beta-Catenin Gene in Primary Hepatocellular Carcinomas by Somatic Alterations Involving Exon 3. *Cancer Res* **1998**, *58*, 2524–2527.

46. Hsieh, J.C.; Kodjabachian, L.; Rebbert, M.L.; Rattner, A.; Smallwood, P.M.; Samos, C.H.; Nusse, R.; Dawid, I.B.; Nathans, J. A New Secreted Protein That Binds to Wnt Proteins and Inhibits Their Activities. *Nature* **1999**, *398*, 431–436, doi:10.1038/18899.

47. DasGupta, R.; Fuchs, E. Multiple Roles for Activated LEF/TCF Transcription Complexes during Hair Follicle Development and Differentiation. *Development* **1999**, *126*, 4557–4568, doi:10.1242/dev.126.20.4557.

48. Hamada, F.; Tomoyasu, Y.; Takatsu, Y.; Nakamura, M.; Nagai, S.; Suzuki, A.; Fujita, F.; Shibuya, H.; Toyoshima, K.; Ueno, N.; et al. Negative Regulation of Wingless Signaling by D-Axin, a Drosophila Homolog of Axin. *Science* **1999**, *283*, 1739–1742, doi:10.1126/science.283.5408.1739.

49. Smits, R.; Kielman, M.F.; Breukel, C.; Zurcher, C.; Neufeld, K.; Jagmohan-Changur, S.; Hofland, N.; Van Dijk, J.; White, R.; Edelmann, W.; et al. Apc1638T: A Mouse Model Delineating Critical Domains of the Adenomatous Polyposis Coli Protein Involved in Tumorigenesis and Development. *Genes & Development* **1999**, *13*, 1309–1321, doi:10.1101/gad.13.10.1309.

50. Duval, A.; Gayet, J.; Zhou, X.P.; Iacopetta, B.; Thomas, G.; Hamelin, R. Frequent Frameshift Mutations of the TCF-4 Gene in Colorectal Cancers with Microsatellite Instability. *Cancer Res* **1999**, *59*, 4213–4215.

51. Duval, A.; Iacopetta, B.; Ranzani, G.N.; Lothe, R.A.; Thomas, G.; Hamelin, R. Variable Mutation Frequencies in Coding Repeats of TCF-4 and Other Target Genes in Colon, Gastric and Endometrial Carcinoma Showing Microsatellite Instability. *Oncogene* **1999**, *18*, 6806–6809, doi:10.1038/sj.onc.1203287.

52. Wehrli, M.; Dougan, S.T.; Caldwell, K.; O'Keefe, L.; Schwartz, S.; Vaizel-Ohayon, D.; Schejter, E.; Tomlinson, A.; DiNardo, S. Arrow Encodes an LDL-Receptor-Related Protein Essential for Wingless Signalling. *Nature* **2000**, *407*, 527–530, doi:10.1038/35035110.

53. Pinson, K.I.; Brennan, J.; Monkley, S.; Avery, B.J.; Skarnes, W.C. An LDL-Receptor-Related Protein Mediates Wnt Signalling in Mice. *Nature* **2000**, *407*, 535–538, doi:10.1038/35035124.

54. Tamai, K.; Semenov, M.; Kato, Y.; Spokony, R.; Liu, C.; Katsuyama, Y.; Hess, F.; Saint-Jeannet, J.P.; He, X. LDL-Receptor-Related Proteins in Wnt Signal Transduction. *Nature* **2000**, *407*, 530–535, doi:10.1038/35035117.

55. Rulifson, E.J.; Wu, C.H.; Nusse, R. Pathway Specificity by the Bifunctional Receptor Frizzled Is Determined by Affinity for Wingless. *Mol Cell* **2000**, *6*, 117–126.

56. Patthy, L. The WIF Module. *Trends in Biochemical Sciences* **2000**, *25*, 12–13, doi:10.1016/S0968-0004(99)01504-2.

57. Spink, K.E.; Polakis, P.; Weis, W.I. Structural Basis of the Axin–Adenomatous Polyposis Coli Interaction. *EMBO J* **2000**, *19*, 2270–2279, doi:10.1093/emboj/19.10.2270.

58. Hecht, A.; Vleminckx, K.; Stemmler, M.P.; van Roy, F.; Kemler, R. The P300/CBP Acetyltransferases Function as Transcriptional Coactivators of Beta-Catenin in Vertebrates. *EMBO J* **2000**, *19*, 1839–1850, doi:10.1093/emboj/19.8.1839.

59. Liu, W.; Dong, X.; Mai, M.; Seelan, R.S.; Taniguchi, K.; Krishnadath, K.K.; Halling, K.C.; Cunningham, J.M.; Boardman, L.A.; Qian, C.; et al. Mutations in AXIN2 Cause Colorectal Cancer with Defective Mismatch Repair by Activating Beta-Catenin/TCF Signalling. *Nat Genet* **2000**, *26*, 146–147, doi:10.1038/79859.

60. Satoh, S.; Daigo, Y.; Furukawa, Y.; Kato, T.; Miwa, N.; Nishiwaki, T.; Kawasoe, T.; Ishiguro, H.; Fujita, M.; Tokino, T.; et al. AXIN1 Mutations in Hepatocellular Carcinomas, and Growth Suppression in Cancer Cells by Virus-Mediated Transfer of AXIN1. *Nat Genet* **2000**, *24*, 245–250, doi:10.1038/73448.

61. Bafico, A.; Liu, G.; Yaniv, A.; Gazit, A.; Aaronson, S.A. Novel Mechanism of Wnt Signalling Inhibition Mediated by

Dickkopf-1 Interaction with LRP6/Arrow. *Nat Cell Biol* **2001**, *3*, 683–686, doi:10.1038/35083081.

62. Mao, B.; Wu, W.; Li, Y.; Hoppe, D.; Stannek, P.; Glinka, A.; Niehrs, C. LDL-Receptor-Related Protein 6 Is a Receptor for Dickkopf Proteins. *Nature* **2001**, *411*, 321–325, doi:10.1038/35077108.

63. Semënov, M.V.; Tamai, K.; Brott, B.K.; Kühl, M.; Sokol, S.; He, X. Head Inducer Dickkopf-1 Is a Ligand for Wnt Coreceptor LRP6. *Curr Biol* **2001**, *11*, 951–961, doi:10.1016/s0960-9822(01)00290-1.

64. Tutter, A.V.; Fryer, C.J.; Jones, K.A. Chromatin-Specific Regulation of LEF-1–β-Catenin Transcription Activation and Inhibition in Vitro. *Genes Dev.* **2001**, *15*, 3342–3354, doi:10.1101/gad.946501.

65. Amit, S.; Hatzubai, A.; Birman, Y.; Andersen, J.S.; Ben-Shushan, E.; Mann, M.; Ben-Neriah, Y.; Alkalay, I. Axin-Mediated CKI Phosphorylation of β-Catenin at Ser 45: A Molecular Switch for the Wnt Pathway. *Genes Dev.* **2002**, *16*, 1066–1076, doi:10.1101/gad.230302.

66. Liu, C.; Li, Y.; Semenov, M.; Han, C.; Baeg, G.H.; Tan, Y.; Zhang, Z.; Lin, X.; He, X. Control of Beta-Catenin Phosphorylation/Degradation by a Dual-Kinase Mechanism. *Cell* **2002**, *108*, 837–847, doi:10.1016/s0092-8674(02)00685-2.

67. Jho, E.; Zhang, T.; Domon, C.; Joo, C.-K.; Freund, J.-N.; Costantini, F. Wnt/β-Catenin/Tcf Signaling Induces the Transcription of Axin2, a Negative Regulator of the Signaling Pathway. *Molecular and Cellular Biology* **2002**, *22*, 1172–1183, doi:10.1128/MCB.22.4.1172-1183.2002.

68. Korswagen, H.C.; Coudreuse, D.Y.M.; Betist, M.C.; van de Water, S.; Zivkovic, D.; Clevers, H.C. The Axin-like Protein PRY-1 Is a Negative Regulator of a Canonical Wnt Pathway in C. Elegans. *Genes Dev* **2002**, *16*, 1291–1302, doi:10.1101/gad.981802.

69. Oishi, I.; Suzuki, H.; Onishi, N.; Takada, R.; Kani, S.; Ohkawara, B.; Koshida, I.; Suzuki, K.; Yamada, G.; Schwabe, G.C.; et al. The Receptor Tyrosine Kinase Ror2 Is Involved in Non-Canonical Wnt5a/JNK Signalling Pathway. *Genes Cells* **2003**, *8*, 645–654, doi:10.1046/j.1365-2443.2003.00662.x.

70. Yoshikawa, S.; McKinnon, R.D.; Kokel, M.; Thomas, J.B. Wnt-Mediated Axon Guidance via the Drosophila Derailed Receptor. *Nature* **2003**, *422*, 583–588, doi:10.1038/nature01522.

71. Cliffe, A.; Hamada, F.; Bienz, M. A Role of Dishevelled in Relocating Axin to the Plasma Membrane during Wingless Signaling. *Current Biology* **2003**, *13*, 960–966, doi:10.1016/S0960-9822(03)00370-1.

72. Lee, E.; Salic, A.; Krüger, R.; Heinrich, R.; Kirschner, M.W. The Roles of APC and Axin Derived from Experimental and Theoretical Analysis of the Wnt Pathway. *PLoS Biol* **2003**, *1*, E10, doi:10.1371/journal.pbio.0000010.

73. Ha, N.-C.; Tonozuka, T.; Stamos, J.L.; Choi, H.-J.; Weis, W.I. Mechanism of Phosphorylation-Dependent Binding of APC to β-Catenin and Its Role in β-Catenin Degradation. *Molecular Cell* **2004**, *15*, 511–521, doi:10.1016/j.molcel.2004.08.010.

74. Kuhnert, F.; Davis, C.R.; Wang, H.-T.; Chu, P.; Lee, M.; Yuan, J.; Nusse, R.; Kuo, C.J. Essential Requirement for Wnt Signaling in Proliferation of Adult Small Intestine and Colon Revealed by Adenoviral Expression of Dickkopf-1. *Proc Natl Acad Sci U S A* **2004**, *101*, 266–271, doi:10.1073/pnas.2536800100.

75. Gaspar, C.; Fodde, R. APC Dosage Effects in Tumorigenesis and Stem Cell Differentiation. *Int. J. Dev. Biol.* **2004**, *48*, 377–386, doi:10.1387/ijdb.041807cg.

76. Lammi, L.; Arte, S.; Somer, M.; Järvinen, H.; Lahermo, P.; Thesleff, I.; Pirinen, S.; Nieminen, P. Mutations in AXIN2 Cause Familial Tooth Agenesis and Predispose to Colorectal Cancer. *The American Journal of Human Genetics* **2004**, *74*, 1043–1050, doi:10.1086/386293.

77. Tamai, K.; Zeng, X.; Liu, C.; Zhang, X.; Harada, Y.; Chang, Z.; He, X. A Mechanism for Wnt Coreceptor Activation. *Mol Cell* **2004**, *13*, 149–156, doi:10.1016/s1097-2765(03)00484-2.

78. McMillan, M.; Kahn, M. Investigating Wnt Signaling: A Chemogenomic Safari. *Drug Discov Today* **2005**, *10*, 1467–1474, doi:10.1016/S1359-6446(05)03613-5.

79. Zeng, X.; Tamai, K.; Doble, B.; Li, S.; Huang, H.; Habas, R.; Okamura, H.; Woodgett, J.; He, X. A Dual-Kinase

Mechanism for Wnt Co-Receptor Phosphorylation and Activation. *Nature* **2005**, *438*, 873–877, doi:10.1038/nature04185.

80. Park, J.Y.; Park, W.S.; Nam, S.W.; Kim, S.Y.; Lee, S.H.; Yoo, N.J.; Lee, J.Y.; Park, C.K. Mutations of Beta-Catenin and AXIN I Genes Are a Late Event in Human Hepatocellular Carcinogenesis. *Liver Int* **2005**, *25*, 70–76, doi:10.1111/j.1478-3231.2004.0995.x.

81. Guder, C.; Pinho, S.; Nacak, T.G.; Schmidt, H.A.; Hobmayer, B.; Niehrs, C.; Holstein, T.W. An Ancient Wnt-Dickkopf Antagonism in *Hydra*. *Development* **2006**, *133*, 901–911, doi:10.1242/dev.02265.

82. Bänziger, C.; Soldini, D.; Schütt, C.; Zipperlen, P.; Hausmann, G.; Basler, K. Wntless, a Conserved Membrane Protein Dedicated to the Secretion of Wnt Proteins from Signaling Cells. *Cell* **2006**, *125*, 509–522, doi:10.1016/j.cell.2006.02.049.

83. Ching, W.; Nusse, R. A Dedicated Wnt Secretion Factor. *Cell* **2006**, *125*, 432–433, doi:10.1016/j.cell.2006.04.018.

84. Bartscherer, K.; Pelte, N.; Ingelfinger, D.; Boutros, M. Secretion of Wnt Ligands Requires Evi, a Conserved Transmembrane Protein. *Cell* **2006**, *125*, 523–533, doi:10.1016/j.cell.2006.04.009.

85. Shackleton, M.; Vaillant, F.; Simpson, K.J.; Stingl, J.; Smyth, G.K.; Asselin-Labat, M.-L.; Wu, L.; Lindeman, G.J.; Visvader, J.E. Generation of a Functional Mammary Gland from a Single Stem Cell. *Nature* **2006**, *439*, 84–88, doi:10.1038/nature04372.

86. Schwarz-Romond, T.; Metcalfe, C.; Bienz, M. Dynamic Recruitment of Axin by Dishevelled Protein Assemblies. *Journal of Cell Science* **2007**, *120*, 2402–2412, doi:10.1242/jcs.002956.

87. Sansom, O.J.; Meniel, V.S.; Muncan, V.; Phesse, T.J.; Wilkins, J.A.; Reed, K.R.; Vass, J.K.; Athineos, D.; Clevers, H.; Clarke, A.R. Myc Deletion Rescues Apc Deficiency in the Small Intestine. *Nature* **2007**, *446*, 676–679, doi:10.1038/nature05674.

88. Su, Y.; Fu, C.; Ishikawa, S.; Stella, A.; Kojima, M.; Shitoh, K.; Schreiber, E.M.; Day, B.W.; Liu, B. APC Is Essential for Targeting Phosphorylated β-Catenin to the SCFβ-TrCP Ubiquitin Ligase. *Molecular Cell* **2008**, *32*, 652–661, doi:10.1016/j.molcel.2008.10.023.

89. Säfholm, A.; Tuomela, J.; Rosenkvist, J.; Dejmek, J.; Härkönen, P.; Andersson, T. The Wnt-5a–Derived Hexapeptide Foxy-5 Inhibits Breast Cancer Metastasis *In Vivo* by Targeting Cell Motility. *Clinical Cancer Research* **2008**, *14*, 6556–6563, doi:10.1158/1078-0432.CCR-08-0711.

90. MacDonald, B.T.; Tamai, K.; He, X. Wnt/Beta-Catenin Signaling: Components, Mechanisms, and Diseases. *Dev Cell* **2009**, *17*, 9–26, doi:10.1016/j.devcel.2009.06.016.

91. Pollard, P.; Deheragoda, M.; Segditsas, S.; Lewis, A.; Rowan, A.; Howarth, K.; Willis, L.; Nye, E.; McCart, A.; Mandir, N.; et al. The Apc 1322T Mouse Develops Severe Polyposis Associated with Submaximal Nuclear Beta-Catenin Expression. *Gastroenterology* **2009**, *136*, 2204-2213.e1-13, doi:10.1053/j.gastro.2009.02.058.

92. Gaspar, C.; Franken, P.; Molenaar, L.; Breukel, C.; Van Der Valk, M.; Smits, R.; Fodde, R. A Targeted Constitutive Mutation in the Apc Tumor Suppressor Gene Underlies Mammary But Not Intestinal Tumorigenesis. *PLoS Genet* **2009**, *5*, e1000547, doi:10.1371/journal.pgen.1000547.

93. Huang, S.-M.A.; Mishina, Y.M.; Liu, S.; Cheung, A.; Stegmeier, F.; Michaud, G.A.; Charlat, O.; Wiellette, E.; Zhang, Y.; Wiessner, S.; et al. Tankyrase Inhibition Stabilizes Axin and Antagonizes Wnt Signalling. *Nature* **2009**, *461*, 614–620, doi:10.1038/nature08356.

94. Heath, D.J.; Chantry, A.D.; Buckle, C.H.; Coulton, L.; Shaughnessy, J.D.; Evans, H.R.; Snowden, J.A.; Stover, D.R.; Vanderkerken, K.; Croucher, P.I. Inhibiting Dickkopf-1 (Dkk1) Removes Suppression of Bone Formation and Prevents the Development of Osteolytic Bone Disease in Multiple Myeloma. *J Bone Miner Res* **2009**, *24*, 425–436, doi:10.1359/jbmr.081104.

95. Fulciniti, M.; Tassone, P.; Hideshima, T.; Vallet, S.; Nanjappa, P.; Ettenberg, S.A.; Shen, Z.; Patel, N.; Tai, Y.; Chauhan, D.; et al. Anti-DKK1 mAb (BHQ880) as a Potential Therapeutic Agent for Multiple Myeloma. *Blood* **2009**, *114*, 371–379, doi:10.1182/blood-2008-11-191577.

96. Zeng, Y.A.; Nusse, R. Wnt Proteins Are Self-Renewal Factors for Mammary Stem Cells and Promote Their Long-Term Expansion in Culture. *Cell Stem Cell* **2010**, *6*, 568–577, doi:10.1016/j.stem.2010.03.020.

97. Lewis, A.; Segditsas, S.; Deheragoda, M.; Pollard, P.; Jeffery, R.; Nye, E.; Lockstone, H.; Davis, H.; Clark, S.; Stamp, G.; et al. Severe Polyposis in Apc ^{1322T} Mice Is Associated with Submaximal Wnt Signalling and Increased Expression of the Stem Cell Marker *Lgr5*. *Gut* **2010**, *59*, 1680–1686, doi:10.1136/gut.2009.193680.

98. Chen, W.; Chen, M.; Barak, L.S. Development of Small Molecules Targeting the Wnt Pathway for the Treatment of Colon Cancer: A High-Throughput Screening Approach. *American Journal of Physiology-Gastrointestinal and Liver Physiology* **2010**, *299*, G293–G300, doi:10.1152/ajpgi.00005.2010.

99. Saraswati, S.; Alfaro, M.P.; Thorne, C.A.; Atkinson, J.; Lee, E.; Young, P.P. Pyrvinium, a Potent Small Molecule Wnt Inhibitor, Promotes Wound Repair and Post-MI Cardiac Remodeling. *PLoS ONE* **2010**, *5*, e15521, doi:10.1371/journal.pone.0015521.

100. Thorne, C.A.; Hanson, A.J.; Schneider, J.; Tahinci, E.; Orton, D.; Cselenyi, C.S.; Jernigan, K.K.; Meyers, K.C.; Hang, B.I.; Waterson, A.G.; et al. Small-Molecule Inhibition of Wnt Signaling through Activation of Casein Kinase 1α. *Nat Chem Biol* **2010**, *6*, 829–836, doi:10.1038/nchembio.453.

101. Cha, J.Y.; Jung, J.-E.; Lee, K.-H.; Briaud, I.; Tenzin, F.; Jung, H.K.; Pyon, Y.; Lee, D.; Chung, J.U.; Lee, J.H.; et al. Anti-Tumor Activity of Novel Small Molecule Wnt Signaling Inhibitor, CWP232291, In Multiple Myeloma. *Blood* **2010**, *116*, 3038–3038, doi:10.1182/blood.V116.21.3038.3038.

102. Chen, S.; Bubeck, D.; MacDonald, B.T.; Liang, W.-X.; Mao, J.-H.; Malinauskas, T.; Llorca, O.; Aricescu, A.R.; Siebold, C.; He, X.; et al. Structural and Functional Studies of LRP6 Ectodomain Reveal a Platform for Wnt Signaling. *Developmental Cell* **2011**, *21*, 848–861, doi:10.1016/j.devcel.2011.09.007.

103. Sato, T.; van Es, J.H.; Snippert, H.J.; Stange, D.E.; Vries, R.G.; van den Born, M.; Barker, N.; Shroyer, N.F.; van de Wetering, M.; Clevers, H. Paneth Cells Constitute the Niche for Lgr5 Stem Cells in Intestinal Crypts. *Nature* **2011**, *469*, 415–418, doi:10.1038/nature09637.

104. Roberts, D.M.; Pronobis, M.I.; Poulton, J.S.; Waldmann, J.D.; Stephenson, E.M.; Hanna, S.; Peifer, M. Deconstructing the SScatenin Destruction Complex: Mechanistic Roles for the Tumor Suppressor APC in Regulating Wnt Signaling. *MBoC* **2011**, *22*, 1845–1863, doi:10.1091/mbc.e10-11-0871.

105. Shah, D.A.; Kwon, S.-J.; Bale, S.S.; Banerjee, A.; Dordick, J.S.; Kane, R.S. Regulation of Stem Cell Signaling by Nanoparticle-Mediated Intracellular Protein Delivery. *Biomaterials* **2011**, *32*, 3210–3219, doi:10.1016/j.biomaterials.2010.11.077.

106. Janda, C.Y.; Waghray, D.; Levin, A.M.; Thomas, C.; Garcia, K.C. Structural Basis of Wnt Recognition by Frizzled. *Science* **2012**, *337*, 59–64, doi:10.1126/science.1222879.

107. Tauriello, D.V.F.; Jordens, I.; Kirchner, K.; Slootstra, J.W.; Kruitwagen, T.; Bouwman, B.A.M.; Noutsou, M.; Rüdiger, S.G.D.; Schwamborn, K.; Schambony, A.; et al. Wnt/β-Catenin Signaling Requires Interaction of the Dishevelled DEP Domain and C Terminus with a Discontinuous Motif in Frizzled. *Proc Natl Acad Sci U S A* **2012**, *109*, E812-820, doi:10.1073/pnas.1114802109.

108. Koo, B.-K.; Spit, M.; Jordens, I.; Low, T.Y.; Stange, D.E.; van de Wetering, M.; van Es, J.H.; Mohammed, S.; Heck, A.J.R.; Maurice, M.M.; et al. Tumour Suppressor RNF43 Is a Stem-Cell E3 Ligase That Induces Endocytosis of Wnt Receptors. *Nature* **2012**, *488*, 665–669, doi:10.1038/nature11308.

109. Seshagiri, S.; Stawiski, E.W.; Durinck, S.; Modrusan, Z.; Storm, E.E.; Conboy, C.B.; Chaudhuri, S.; Guan, Y.; Janakiraman, V.; Jaiswal, B.S.; et al. Recurrent R-Spondin Fusions in Colon Cancer. *Nature* **2012**, *488*, 660–664, doi:10.1038/nature11282.

110. Feng, G.J.; Cotta, W.; Wei, X.Q.; Poetz, O.; Evans, R.; Jardé, T.; Reed, K.; Meniel, V.; Williams, G.T.; Clarke, A.R.; et al. Conditional Disruption of Axin1 Leads to Development of Liver Tumors in Mice. *Gastroenterology* **2012**, *143*, 1650–165[1]9, doi:10.1053/j.gastro.2012.08.047.

111. Chen, P.-H.; Chen, X.; Lin, Z.; Fang, D.; He, X. The Structural Basis of R-Spondin Recognition by LGR5 and RNF43. *Genes Dev.* **2013**, *27*, 1345–1350, doi:10.1101/gad.219915.113.

112. Yang, L.-H.; Xu, H.-T.; Li, Q.-C.; Jiang, G.-Y.; Zhang, X.-P.; Zhao, H.-Y.; Xu, K.; Wang, E.-H. Abnormal Hypermethylation and Clinicopathological Significance of Axin Gene in Lung Cancer. *Tumor Biol.* **2013**, *34*, 749–757, doi:10.1007/s13277-012-0604-z.

113. Voloshanenko, O.; Erdmann, G.; Dubash, T.D.; Augustin, I.; Metzig, M.; Moffa, G.; Hundsrucker, C.; Kerr, G.; Sandmann, T.; Anchang, B.; et al. Wnt Secretion Is Required to Maintain High Levels of Wnt Activity in Colon Cancer Cells. *Nat Commun* **2013**, *4*, 2610, doi:10.1038/ncomms3610.

114. Leedham, S.J.; Rodenas-Cuadrado, P.; Howarth, K.; Lewis, A.; Mallappa, S.; Segditsas, S.; Davis, H.; Jeffery, R.; Rodriguez-Justo, M.; Keshav, S.; et al. A Basal Gradient of Wnt and Stem-Cell Number Influences Regional Tumour Distribution in Human and Mouse Intestinal Tracts. *Gut* **2013**, *62*, 83–93, doi:10.1136/gutjnl-2011-301601.

115. Jiang, X.; Hao, H.-X.; Growney, J.D.; Woolfenden, S.; Bottiglio, C.; Ng, N.; Lu, B.; Hsieh, M.H.; Bagdasarian, L.; Meyer, R.; et al. Inactivating Mutations of RNF43 Confer Wnt Dependency in Pancreatic Ductal Adenocarcinoma. *Proc Natl Acad Sci U S A* **2013**, *110*, 12649–12654, doi:10.1073/pnas.1307218110.

116. Liu, J.; Pan, S.; Hsieh, M.H.; Ng, N.; Sun, F.; Wang, T.; Kasibhatla, S.; Schuller, A.G.; Li, A.G.; Cheng, D.; et al. Targeting Wnt-Driven Cancer through the Inhibition of Porcupine by LGK974. *Proc Natl Acad Sci U S A* **2013**, *110*, 20224–20229, doi:10.1073/pnas.1314239110.

117. Smith, D.C.; Rosen, L.S.; Chugh, R.; Goldman, J.W.; Xu, L.; Kapoun, A.; Brachmann, R.K.; Dupont, J.; Stagg, R.J.; Tolcher, A.W.; et al. First-in-Human Evaluation of the Human Monoclonal Antibody Vantictumab (OMP-18R5; Anti-Frizzled) Targeting the WNT Pathway in a Phase I Study for Patients with Advanced Solid Tumors. *JCO* **2013**, *31*, 2540–2540, doi:10.1200/jco.2013.31.15_suppl.2540.

118. Ma, P.; Yang, X.; Kong, Q.; Li, C.; Yang, S.; Li, Y.; Mao, B. The Ubiquitin Ligase RNF220 Enhances Canonical Wnt Signaling through USP7-Mediated Deubiquitination of β-Catenin. *Molecular and Cellular Biology* **2014**, *34*, 4355–4366, doi:10.1128/MCB.00731-14.

119. Giannakis, M.; Hodis, E.; Jasmine Mu, X.; Yamauchi, M.; Rosenbluh, J.; Cibulskis, K.; Saksena, G.; Lawrence, M.S.; Qian, Z.R.; Nishihara, R.; et al. RNF43 Is Frequently Mutated in Colorectal and Endometrial Cancers. *Nat Genet* **2014**, *46*, 1264–1266, doi:10.1038/ng.3127.

120. Gao, Y.; Song, C.; Hui, L.; Li, C.; Wang, J.; Tian, Y.; Han, X.; Chen, Y.; Tian, D.-L.; Qiu, X.; et al. Overexpression of RNF146 in Non-Small Cell Lung Cancer Enhances Proliferation and Invasion of Tumors through the Wnt/β-Catenin Signaling Pathway. *PLoS ONE* **2014**, *9*, e85377, doi:10.1371/journal.pone.0085377.

121. Rivera, B.; Perea, J.; Sánchez, E.; Villapún, M.; Sánchez-Tomé, E.; Mercadillo, F.; Robledo, M.; Benítez, J.; Urioste, M. A Novel AXIN2 Germline Variant Associated with Attenuated FAP without Signs of Oligondontia or Ectodermal Dysplasia. *Eur J Hum Genet* **2014**, *22*, 423–426, doi:10.1038/ejhg.2013.146.

122. Jiang, X.; Charlat, O.; Zamponi, R.; Yang, Y.; Cong, F. Dishevelled Promotes Wnt Receptor Degradation through Recruitment of ZNRF3/RNF43 E3 Ubiquitin Ligases. *Molecular Cell* **2015**, *58*, 522–533, doi:10.1016/j.molcel.2015.03.015.

123. Dow, L.E.; O'Rourke, K.P.; Simon, J.; Tschaharganeh, D.F.; van Es, J.H.; Clevers, H.; Lowe, S.W. Apc Restoration Promotes Cellular Differentiation and Reestablishes Crypt Homeostasis in Colorectal Cancer. *Cell* **2015**, *161*, 1539–1552, doi:10.1016/j.cell.2015.05.033.

124. McGonigle, S.; Chen, Z.; Wu, J.; Chang, P.; Kolber-Simonds, D.; Ackermann, K.; Twine, N.C.; Shie, J.-L.; Miu, J.T.; Huang, K.-C.; et al. E7449: A Dual Inhibitor of PARP1/2 and Tankyrase1/2 Inhibits Growth of DNA Repair Deficient Tumors and Antagonizes Wnt Signaling. *Oncotarget* **2015**, *6*, 41307–41323, doi:10.18632/oncotarget.5846.

125. Koo, B.-K.; van Es, J.H.; van den Born, M.; Clevers, H. Porcupine Inhibitor Suppresses Paracrine Wnt-Driven Growth of Rnf43;Znrf3-Mutant Neoplasia. *Proc Natl Acad Sci U S A* **2015**, *112*, 7548–7550, doi:10.1073/pnas.1508113112.

126. Mariotti, L.; Templeton, C.M.; Ranes, M.; Paracuellos, P.; Cronin, N.; Beuron, F.; Morris, E.; Guettler, S. Tankyrase Requires SAM Domain-Dependent Polymerization to Support Wnt-β-Catenin Signaling. *Molecular Cell* **2016**, *63*, 498–513, doi:10.1016/j.molcel.2016.06.019.

127. Croy, H.E.; Fuller, C.N.; Giannotti, J.; Robinson, P.; Foley, A.V.A.; Yamulla, R.J.; Cosgriff, S.; Greaves, B.D.; Von Kleeck, R.A.; An, H.H.; et al. The Poly(ADP-Ribose) Polymerase Enzyme Tankyrase Antagonizes Activity of the β-

Catenin Destruction Complex through ADP-Ribosylation of Axin and APC2. *Journal of Biological Chemistry* **2016**, *291*, 12747–12760, doi:10.1074/jbc.M115.705442.

128. Sekine, S.; Yamashita, S.; Tanabe, T.; Hashimoto, T.; Yoshida, H.; Taniguchi, H.; Kojima, M.; Shinmura, K.; Saito, Y.; Hiraoka, N.; et al. Frequent *PTPRK-RSPO3* Fusions and *RNF43* Mutations in Colorectal Traditional Serrated Adenoma: *RSPO3* Fusions and *RNF43* Mutations in Colorectal TSA. *J. Pathol.* **2016**, *239*, 133–138, doi:10.1002/path.4709.

129. Rosales-Reynoso, M.A.; Arredondo-Valdez, A.R.; Wence-Chávez, L.I.; Barros-Núñez, P.; Gallegos-Arreola, M.P.; Flores-Martínez, S.E.; Sánchez-Corona, J. *AXIN2* Polymorphisms and Their Association with Colorectal Cancer in Mexican Patients. *Genetic Testing and Molecular Biomarkers* **2016**, *20*, 438–444, doi:10.1089/gtmb.2016.0026.

130. Madan, B.; Ke, Z.; Harmston, N.; Ho, S.Y.; Frois, A.O.; Alam, J.; Jeyaraj, D.A.; Pendharkar, V.; Ghosh, K.; Virshup, I.H.; et al. Wnt Addiction of Genetically Defined Cancers Reversed by PORCN Inhibition. *Oncogene* **2016**, *35*, 2197–2207, doi:10.1038/onc.2015.280.

131. Han, T.; Schatoff, E.M.; Murphy, C.; Zafra, M.P.; Wilkinson, J.E.; Elemento, O.; Dow, L.E. R-Spondin Chromosome Rearrangements Drive Wnt-Dependent Tumour Initiation and Maintenance in the Intestine. *Nat Commun* **2017**, *8*, 15945, doi:10.1038/ncomms15945.

132. Daly, C.S.; Shaw, P.; Ordonez, L.D.; Williams, G.T.; Quist, J.; Grigoriadis, A.; Van Es, J.H.; Clevers, H.; Clarke, A.R.; Reed, K.R. Functional Redundancy between Apc and Apc2 Regulates Tissue Homeostasis and Prevents Tumorigenesis in Murine Mammary Epithelium. *Oncogene* **2017**, *36*, 1793–1803, doi:10.1038/onc.2016.342.

133. Picco, G.; Petti, C.; Centonze, A.; Torchiaro, E.; Crisafulli, G.; Novara, L.; Acquaviva, A.; Bardelli, A.; Medico, E. Loss of AXIN1 Drives Acquired Resistance to WNT Pathway Blockade in Colorectal Cancer Cells Carrying RSPO 3 Fusions. *EMBO Mol Med* **2017**, *9*, 293–303, doi:10.15252/emmm.201606773.

134. Bahl, C.; Sharma, S.; Singh, N.; Behera, D. Association Study between Genetic Variations in *Axin2* Gene and Lung Cancer Risk in North Indian Population: A Multiple Interaction Analysis. *Tumour Biol.* **2017**, *39*, 101042831769553, doi:10.1177/1010428317695533.

135. Jimeno, A.; Gordon, M.; Chugh, R.; Messersmith, W.; Mendelson, D.; Dupont, J.; Stagg, R.; Kapoun, A.M.; Xu, L.; Uttamsingh, S.; et al. A First-in-Human Phase I Study of the Anticancer Stem Cell Agent Ipafricept (OMP-54F28), a Decoy Receptor for Wnt Ligands, in Patients with Advanced Solid Tumors. *Clinical Cancer Research* **2017**, *23*, 7490–7497, doi:10.1158/1078-0432.CCR-17-2157.

136. Tanaka, N.; Mashima, T.; Mizutani, A.; Sato, A.; Aoyama, A.; Gong, B.; Yoshida, H.; Muramatsu, Y.; Nakata, K.; Matsuura, M.; et al. *APC* Mutations as a Potential Biomarker for Sensitivity to Tankyrase Inhibitors in Colorectal Cancer. *Molecular Cancer Therapeutics* **2017**, *16*, 752–762, doi:10.1158/1535-7163.MCT-16-0578.

137. Bhamra, I.; Adams, N.; Armer, R.; Bingham, M.; McKeever, H.; Phillips, C.; Thompson, B.; Woodcock, S. Novel Porcupine (PORCN) Inhibitor RXC004: Evaluation in Models of RNF43 Loss of Function Cancers. *JCO* **2017**, *35*, e14094–e14094, doi:10.1200/JCO.2017.35.15_suppl.e14094.

138. Lebensohn, A.M.; Rohatgi, R. R-Spondins Can Potentiate WNT Signaling without LGRs. *Elife* **2018**, *7*, e33126, doi:10.7554/eLife.33126.

139. Park, S.; Cui, J.; Yu, W.; Wu, L.; Carmon, K.S.; Liu, Q.J. Differential Activities and Mechanisms of the Four R-Spondins in Potentiating Wnt/β-Catenin Signaling. *Journal of Biological Chemistry* **2018**, *293*, 9759–9769, doi:10.1074/jbc.RA118.002743.

140. Schaefer, K.N.; Bonello, T.T.; Zhang, S.; Williams, C.E.; Roberts, D.M.; McKay, D.J.; Peifer, M. Supramolecular Assembly of the Beta-Catenin Destruction Complex and the Effect of Wnt Signaling on Its Localization, Molecular Size, and Activity in Vivo. *PLoS Genet* **2018**, *14*, e1007339, doi:10.1371/journal.pgen.1007339.

141. He, Y.; Sun, L.-Y.; Wang, J.; Gong, R.; Shao, Q.; Zhang, Z.-C.; Ye, Z.-L.; Wang, H.-Y.; Xu, R.-H.; Shao, J.-Y. Hypermethylation of APC2 Is a Predictive Epigenetic Biomarker for Chinese Colorectal Cancer. *Disease Markers* **2018**, *2018*, 1–7, doi:10.1155/2018/8619462.

142. Shen, J.; Yu, Z.; Li, N. The E3 Ubiquitin Ligase RNF146 Promotes Colorectal Cancer by Activating the Wnt/β-Catenin Pathway via Ubiquitination of Axin1. *Biochemical and Biophysical Research Communications* **2018**, *503*, 991–997, doi:10.1016/j.bbrc.2018.06.107.

143. Eto, T.; Miyake, K.; Nosho, K.; Ohmuraya, M.; Imamura, Y.; Arima, K.; Kanno, S.; Fu, L.; Kiyozumi, Y.; Izumi, D.; et al. Impact of Loss-of-function Mutations at the *RNF43* Locus on Colorectal Cancer Development and Progression. *The Journal of Pathology* **2018**, *245*, 445–455, doi:10.1002/path.5098.

144. Quintana, I.; Mejías-Luque, R.; Terradas, M.; Navarro, M.; Piñol, V.; Mur, P.; Belhadj, S.; Grau, E.; Darder, E.; Solanes, A.; et al. Evidence Suggests That Germline RNF43 Mutations Are a Rare Cause of Serrated Polyposis. *Gut* **2018**, *67*, 2230–2232, doi:10.1136/gutjnl-2017-315733.

145. Giraudet, A.-L.; Cassier, P.A.; Iwao-Fukukawa, C.; Garin, G.; Badel, J.-N.; Kryza, D.; Chabaud, S.; Gilles-Afchain, L.; Clapisson, G.; Desuzinges, C.; et al. A First-in-Human Study Investigating Biodistribution, Safety and Recommended Dose of a New Radiolabeled MAb Targeting FZD10 in Metastatic Synovial Sarcoma Patients. *BMC Cancer* **2018**, *18*, 646, doi:10.1186/s12885-018-4544-x.

146. Inglis, D.J.; Licari, J.; Georgiou, K.R.; Wittwer, N.L.; Hamilton, R.W.; Beaumont, D.M.; Scherer, M.A.; Lavranos, T.C. Abstract 3910: Characterization of BNC101 a Human Specific Monoclonal Antibody Targeting the GPCR LGR5: First-in-Human Evidence of Target Engagement. *Cancer Research* **2018**, *78*, 3910–3910, doi:10.1158/1538-7445.AM2018-3910.

147. Nomura, M.; Rainusso, N.C.; Han, R.; Larson, J.; Shuck, R.L.; Kurenbekova, L.; Yustein, J.T. Abstract 3186: Tegavivint Suppresses Progression and Metastasis of Osteosarcoma via Blockade of Wnt Signaling/ALDH1 Axis: Preclinical Study of a Novel Wnt/β-Catenin Pathway Inhibitor. *Cancer Research* **2018**, *78*, 3186–3186, doi:10.1158/1538-7445.AM2018-3186.

148. Basham, K.J.; Rodriguez, S.; Turcu, A.F.; Lerario, A.M.; Logan, C.Y.; Rysztak, M.R.; Gomez-Sanchez, C.E.; Breault, D.T.; Koo, B.-K.; Clevers, H.; et al. A ZNRF3-Dependent Wnt/β-Catenin Signaling Gradient Is Required for Adrenal Homeostasis. *Genes Dev.* **2019**, *33*, 209–220, doi:10.1101/gad.317412.118.

149. Ji, L.; Lu, B.; Zamponi, R.; Charlat, O.; Aversa, R.; Yang, Z.; Sigoillot, F.; Zhu, X.; Hu, T.; Reece-Hoyes, J.S.; et al. USP7 Inhibits Wnt/β-Catenin Signaling through Promoting Stabilization of Axin. *Nat Commun* **2019**, *10*, 4184, doi:10.1038/s41467-019-12143-3.

150. Neumeyer, V.; Grandl, M.; Dietl, A.; Brutau-Abia, A.; Allgäuer, M.; Kalali, B.; Zhang, Y.; Pan, K.-F.; Steiger, K.; Vieth, M.; et al. Loss of Endogenous RNF43 Function Enhances Proliferation and Tumour Growth of Intestinal and Gastric Cells. *Carcinogenesis* **2019**, *40*, 551–559, doi:10.1093/carcin/bgy152.

151. Otero, L.; Lacunza, E.; Vasquez, V.; Arbelaez, V.; Cardier, F.; González, F. Variations in AXIN2 Predict Risk and Prognosis of Colorectal Cancer. *BDJ Open* **2019**, *5*, 13, doi:10.1038/s41405-019-0022-z.

152. Datta, S.; Choudhury, D.; Das, A.; Mukherjee, D.D.; Dasgupta, M.; Bandopadhyay, S.; Chakrabarti, G. Autophagy Inhibition with Chloroquine Reverts Paclitaxel Resistance and Attenuates Metastatic Potential in Human Nonsmall Lung Adenocarcinoma A549 Cells via ROS Mediated Modulation of β-Catenin Pathway. *Apoptosis* **2019**, *24*, 414–433, doi:10.1007/s10495-019-01526-y.

153. Dubey, R.; van Kerkhof, P.; Jordens, I.; Malinauskas, T.; Pusapati, G.V.; McKenna, J.K.; Li, D.; Carette, J.E.; Ho, M.; Siebold, C.; et al. R-Spondins Engage Heparan Sulfate Proteoglycans to Potentiate WNT Signaling. *Elife* **2020**, *9*, e54469, doi:10.7554/eLife.54469.

154. Spit, M.; Fenderico, N.; Jordens, I.; Radaszkiewicz, T.; Lindeboom, R.G.; Bugter, J.M.; Cristobal, A.; Ootes, L.; Van Osch, M.; Janssen, E.; et al. RNF 43 Truncations Trap CK 1 to Drive Niche-independent Self-renewal in Cancer. *The EMBO Journal* **2020**, *39*, e103932, doi:10.15252/embj.2019103932.

155. Li, S.; Lavrijsen, M.; Bakker, A.; Magierowski, M.; Magierowska, K.; Liu, P.; Wang, W.; Peppelenbosch, M.P.; Smits, R. Commonly Observed RNF43 Mutations Retain Functionality in Attenuating Wnt/β-Catenin Signaling and Unlikely Confer Wnt-Dependency onto Colorectal Cancers. *Oncogene* **2020**, *39*, 3458–3472, doi:10.1038/s41388-020-1232-5.

156. Nie, S.; Wang, Z.; Moscoso-Castro, M.; D'Souza, P.; Lei, C.; Xu, J.; Gu, J. Biology Drives the Discovery of Bispecific Antibodies as Innovative Therapeutics. *Antibody Therapeutics* **2020**, *3*, 18–62, doi:10.1093/abt/tbaa003.

157. Wall, J.A.; Klempner, S.J.; Arend, R.C. The Anti-DKK1 Antibody DKN-01 as an Immunomodulatory Combination Partner for the Treatment of Cancer. *Expert Opinion on Investigational Drugs* **2020**, *29*, 639–644, doi:10.1080/13543784.2020.1769065.

158. Zhang, M.; Haughey, M.; Wang, N.-Y.; Blease, K.; Kapoun, A.M.; Couto, S.; Belka, I.; Hoey, T.; Groza, M.; Hartke, J.; et al. Targeting the Wnt Signaling Pathway through R-Spondin 3 Identifies an Anti-Fibrosis Treatment Strategy for Multiple Organs. *PLoS ONE* **2020**, *15*, e0229445, doi:10.1371/journal.pone.0229445.

159. Tam, B.Y.; Chiu, K.; Chung, H.; Bossard, C.; Nguyen, J.D.; Creger, E.; Eastman, B.W.; Mak, C.C.; Ibanez, M.; Ghias, A.; et al. The CLK Inhibitor SM08502 Induces Anti-Tumor Activity and Reduces Wnt Pathway Gene Expression in Gastrointestinal Cancer Models. *Cancer Letters* **2020**, *473*, 186–197, doi:10.1016/j.canlet.2019.09.009.

160. Im, S.-A.; Lee, S.; Lee, K.W.; Lee, Y.; Sohn, J.; Kim, J.H.; Im, Y.-H.; Park, K.H.; Oh, D.-Y.; Kim, M.H.; et al. A Phase I Dose-Escalation and Expansion Study of JPI-547, a Dual Inhibitor of PARP/Tankyrase in Patients with Advanced Solid Tumors. *JCO* **2021**, *39*, 3113–3113, doi:10.1200/JCO.2021.39.15_suppl.3113.

161. Yamada, K.; Hori, Y.; Inoue, S.; Yamamoto, Y.; Iso, K.; Kamiyama, H.; Yamaguchi, A.; Kimura, T.; Uesugi, M.; Ito, J.; et al. E7386, a Selective Inhibitor of the Interaction between β-Catenin and CBP, Exerts Antitumor Activity in Tumor Models with Activated Canonical Wnt Signaling. *Cancer Research* **2021**, *81*, 1052–1062, doi:10.1158/0008-5472.CAN-20-0782.

162. Élez, E.; Lenz, H.-J.; De Jonge, M.; Yaeger, R.; Doi, T.; Pronk, L.; Teufel, M.; Marzin, K.; Tabernero, J. Abstract CT514: A Phase I, Open-Label, Dose-Escalation Study Investigating a Low-Density Lipoprotein Receptor-Related Protein (LRP) 5/6 Inhibitor, BI 905677, in Patients with Advanced Solid Tumors. *Cancer Research* **2022**, *82*, CT514–CT514, doi:10.1158/1538-7445.AM2022-CT514.

163. Tsutsumi, N.; Hwang, S.; Waghray, D.; Hansen, S.; Jude, K.M.; Wang, N.; Miao, Y.; Glassman, C.R.; Caveney, N.A.; Janda, C.Y.; et al. Structure of the Wnt-Frizzled-LRP6 Initiation Complex Reveals the Basis for Coreceptor Discrimination. *Proc Natl Acad Sci U S A* **2023**, *120*, e2218238120, doi:10.1073/pnas.2218238120.

164. Pascual-Carreras, E.; Marín-Barba, M.; Castillo-Lara, S.; Coronel-Córdoba, P.; Magri, M.S.; Wheeler, G.N.; Gómez-Skarmeta, J.L.; Abril, J.F.; Saló, E.; Adell, T. Wnt/β-Catenin Signalling Is Required for Pole-Specific Chromatin Remodeling during Planarian Regeneration. *Nat Commun* **2023**, *14*, 298, doi:10.1038/s41467-023-35937-y.

165. Yang, Q.; Qin, T.; An, T.; Wu, H.; Xu, G.; Xiang, J.; Lei, K.; Zhang, S.; Xia, J.; Su, G.; et al. Novel PORCN Inhibitor WHN-88 Targets Wnt/β-Catenin Pathway and Prevents the Growth of Wnt-Driven Cancers. *European Journal of Pharmacology* **2023**, *945*, 175628, doi:10.1016/j.ejphar.2023.175628.

166. Wu, X.; Zhong, Y.; Zhang, H.; Li, M. MiR-5590-3p Inhibits the Proliferation and Invasion of Ovarian Cancer Cells through Mediating the Wnt/β-Catenin Signaling Pathway by Targeting TNIK. *Histol Histopathol* **2024**, *39*, 345–355, doi:10.14670/HH-18-636.

167. Rajabi, A.; Nejati, M.; Homayoonfal, M.; Arj, A.; Razavi, Z.S.; Ostadian, A.; Mohammadzadeh, B.; Vosough, M.; Karimi, M.; Rahimian, N.; et al. Doxorubicin-Loaded Zymosan Nanoparticles: Synergistic Cytotoxicity and Modulation of Apoptosis and Wnt/β-Catenin Signaling Pathway in C26 Colorectal Cancer Cells. *International Journal of Biological Macromolecules* **2024**, *260*, 128949, doi:10.1016/j.ijbiomac.2023.128949.

168. Gutova, M.; Hibbard, J.C.; Ma, E.; Natri, H.M.; Adhikarla, V.; Chimge, N.-O.; Qiu, R.; Nguyen, C.; Melendez, E.; Aguilar, B.; et al. Targeting Wnt Signaling for Improved Glioma Immunotherapy. *Front Immunol* **2024**, *15*, 1342625, doi:10.3389/fimmu.2024.1342625.

169. Mortezaee, K. WNT/β-Catenin Regulatory Roles on PD-(L)1 and Immunotherapy Responses. *Clin Exp Med* **2024**, *24*, 15, doi:10.1007/s10238-023-01274-z.
